# Child Mortality Estimation: Accelerated Progress in Reducing Global Child Mortality, 1990–2010

**DOI:** 10.1371/journal.pmed.1001303

**Published:** 2012-08-28

**Authors:** Kenneth Hill, Danzhen You, Mie Inoue, Mikkel Z. Oestergaard, Technical Advisory Group of the United Nations Inter-agency Group for Child Mortality Estimation, Kenneth Hill, Leontine Alkema, Simon Cousens, Trevor Croft, Michel Guillot, Jon Pedersen, Neff Walker, John Wilmoth, Gareth Jones

**Affiliations:** Harvard University; National University of Singapore; London School of Hygiene and Tropical Medicine; Measure DHS, ICF Macro; University of Pennsylvania; Fafo; Johns Hopkins University; University of California, Berkeley; Consultant; 1 Harvard Center for Population and Development Studies, Harvard School of Public Health, Cambridge, Massachusetts, United States of America; 2 United Nations Children's Fund, New York, New York, United States of America; 3 Department of Health Statistics and Information Systems, World Health Organization, Geneva, Switzerland; Umeå Centre for Global Health Research, Umeå University, Sweden

## Abstract

Monitoring development indicators has become a central interest of international agencies and countries for tracking progress towards the Millennium Development Goals. In this review, which also provides an introduction to a collection of articles, we describe the methodology used by the United Nations Inter-agency Group for Child Mortality Estimation to track country-specific changes in the key indicator for Millennium Development Goal 4 (MDG 4), the decline of the under-five mortality rate (the probability of dying between birth and age five, also denoted in the literature as U5MR and _5_ *q*_0_). We review how relevant data from civil registration, sample registration, population censuses, and household surveys are compiled and assessed for United Nations member states, and how time series regression models are fitted to all points of acceptable quality to establish the trends in U5MR from which infant and neonatal mortality rates are generally derived. The application of this methodology indicates that, between 1990 and 2010, the global U5MR fell from 88 to 57 deaths per 1,000 live births, and the annual number of under-five deaths fell from 12.0 to 7.6 million. Although the annual rate of reduction in the U5MR accelerated from 1.9% for the period 1990–2000 to 2.5% for the period 2000–2010, it remains well below the 4.4% annual rate of reduction required to achieve the MDG 4 goal of a two-thirds reduction in U5MR from its 1990 value by 2015. Thus, despite progress in reducing child mortality worldwide, and an encouraging increase in the pace of decline over the last two decades, MDG 4 will not be met without greatly increasing efforts to reduce child deaths.

## Abstract 2

Kenneth Hill and colleagues provide an introductory overview of how the latest United Nations Inter-agency Group for Child Mortality Estimation estimates were produced, summarizes the key findings of these estimates and describe current methodology and recent methodological innovations.

## Introduction

Evidence-based estimation of child mortality is a cornerstone for tracking progress towards Millennium Development Goal 4 (MDG 4), which calls for a two-thirds reduction in the under-five mortality rate (the probability of dying between birth and age five, also denoted in the literature as U5MR and _5_ *q*_0_) between 1990 and 2015, and for planning national and global health strategies, policies, and interventions on child health. With only a few years to the Millennium Development Goal (MDG) target year of 2015, and with increasing attention being paid to evidence-based interventions to reduce child mortality, the demand for frequent, accurate, and transparent monitoring of U5MRs is increasing [1],[2].

To this end, the United Nations Inter-agency Group for Child Mortality Estimation (UN IGME) was formed in 2004. Four United Nations agencies—the United Nations Children's Fund (UNICEF), the World Health Organization (WHO), the United Nations Population Division, and the World Bank—collaborate in UN IGME to produce annual updates, typically released in September, of neonatal, infant, and under-five mortality levels and trends for all United Nations member states—195 countries or entities at the time of the most recent estimation. Each annual update incorporates both new data and improvements in estimation methods, thereby both providing estimates for more recent periods and revising historical trends.

An independent Technical Advisory Group of the UN IGME (TAG), composed of independent experts in demography and biostatistics, provides UN IGME with guidance on strategies and methods for data quality assessment and estimation methods. As part of this process, the TAG meets twice yearly to identify priorities for new methodological work, to review new research relevant to child mortality estimation (CME), and to make specific recommendations to UN IGME on methods.

In this article, we provide an overview of how the latest UN IGME estimates were produced, and summarize the key findings of these estimates [2]. Our article provides an introduction to the 2012 *PLOS Medicine* Collection “Child Mortality Estimation Methods,” a collection of articles that describe both the current methodology used by UN IGME and recent methodological innovations introduced on the recommendation of the TAG (see Table 1 for a list of the papers in the collection and brief descriptions of their topics). Together this collection provides a full picture of the development of UN IGME estimation methods.

**Table 1:** Articles in the *PLOS Medicine* Collection “Child Mortality Estimation Methods.”

Author(s)	Title	Topic(s)
Hill K, You D, Inoue M, Oestergaard MZ	Child mortality estimation: accelerated progress in reducing global child mortality, 1990–2010 (this article)	Overview of UN IGME methodology and key results
Pedersen J, Liu J	Child mortality estimation: appropriate time periods for child mortality estimates from full birth histories [4]	Statistical basis for optimizing choice of time periods for which FBH estimates are calculated
Sawyer CC	Child mortality estimation: estimating sex differences in childhood mortality since the 1970s [16]	Estimating and modeling sex differences in child mortality
Walker N, Hill K, Zhao F	Child mortality estimation: methods used to adjust for bias due to AIDS in estimating trends in under-five mortality [8]	Identification and quantification of, and adjustment for, selection bias in direct child mortality estimates
Silva R	Child mortality estimation: consistency of under-five mortality rate estimates using full birth histories and summary birth histories [9]	Identification and quantification of differences between direct and indirect estimates of child mortality from FBHs
Alkema L, You D	Child mortality estimation: a comparison of UN IGME and IHME estimates of levels and trends in under-five mortality rates and deaths [19]	A comparison and decomposition of differences between UN IGME child mortality estimates and those from the Institute for Health Metrics and Evaluation
Guillot M, Gerland P, Pelletier F, Saabneh A	Child mortality estimation: a global overview of infant and child mortality age patterns in light of new empirical data [10]	An assessment of how well existing mortality models capture real age patterns of mortality in childhood

## How UN IGME Estimates Child Mortality

The fundamental strategy of the UN IGME estimation process is to compile all nationally representative data relevant to child mortality, adjust these data for biases if needed, and then use statistical models to derive smoothed time series for three indicators: the neonatal mortality rate (NMR, probability of dying within the first month of life), the infant mortality rate (IMR, probability of dying before first birthday), and the U5MR (probability of dying before fifth birthday). For IMR and U5MR, a country-specific local regression model is fitted to observations for one of the two indicators, and a model life table is then used to derive the other. UN IGME has developed a separate multilevel statistical model to derive NMR trends and levels based on estimated U5MR [3]. The estimated mortality rate time series are used to calculate numbers of neonatal, infant, and under-five deaths. Finally, a country consultation process is undertaken to maximize identification of all relevant data and to allow countries to review and provide feedback on estimates.

### Data Identification and the CME Info Database

The four agencies in UN IGME share responsibility for populating and updating CME Info (http://www.childmortality.org), the database launched by UN IGME in 2008 to compile and share underlying data, to publish latest estimates on child mortality, and to serve as a platform for collaboration with national partners in harmonizing and disseminating child mortality data and estimates. Wide use of CME Info has increased the transparency of the UN IGME estimation process by allowing users to access the data and methodology used for CME in individual countries.

Data from several nationally representative sources are used by UN IGME (Box 1; Table S1). CME Info does not include information from sub-national studies such as from nonrepresentative demographic surveillance sites, from routine health sector statistics, or from survey data on survival of recent births.

**Box 1. Data Sources Used by UN IGME**

**Vital Statistics**

Civil registration systems collect administrative records of births and deaths prospectively and continuously, which makes this the preferred source of data. However, many developing countries do not have well-functioning civil registration systems.

**Full Birth Histories from Nationally Representative Surveys**

A FBH is a record of all the live births a woman has had, and is collected in surveys such as those conducted by DHS. Typically, information is collected on the date of birth of each child, whether the child is still alive, and, if the child has died, the age at death. If all women in a country who had given birth in some time period before the survey were interviewed, and all reporting was accurate, such information would be equivalent to complete civil registration for the time period. In practice, such information is collected from a sample of surviving women, and the information collected is subject to certain errors due to the retrospective data collection. However, in terms of analysis, the data are treated similarly to vital statistics, and occurrence/exposure rates for specified time periods are calculated for narrow age intervals of the children. The use of FBHs in developing countries was pioneered by the World Fertility Survey (1975–1984). The largest source of FBHs is the DHS project, which has conducted over 200 such surveys since 1986.

A FBH includes all the information needed to tabulate summary birth histories (see below), which can then be analyzed separately. Because the estimates derived directly and indirectly from FBHs may be affected differently by various biases in the data, in the 2011 estimates presented in [2] and this paper UN IGME included both direct and indirect estimates from FBHs when fitting trends.

**Summary Birth Histories from Nationally Representative Surveys**

A summary birth history is a record of the number of live births a woman has had and of the number of children still surviving at the time of the survey (or, equivalently, the number of children who have died). This type of data is collected by censuses, DHS surveys, and Multiple Indicator Cluster Surveys. No information is collected about individual children. Estimates of child mortality are derived from proportions dead of children ever born, typically calculated by five-year age groups of the mother: the age of the mother is a proxy for the duration and pattern of exposure to risk of the children. Models or other representations of age patterns of fertility and mortality have been used to develop methods for converting proportions dead into probabilities of dying by exact ages of childhood and to provide estimates of the time reference of such estimates [14],[20],[21]. Since the estimates depend on models of fertility and mortality patterns by age, the accuracy of the estimates depends on the appropriateness of the models for the population being studied. As a result, such estimates are often described as “indirect.”

Both UNICEF and WHO regularly seek out new or overlooked national data sources to update CME Info. UNICEF conducts an annual exercise through its offices in over 150 countries around the world to gather recent information for all indicators regularly reported on by UNICEF, including neonatal, infant, and under-five mortality rates. Similarly, WHO compiles regular reports from about 110 countries on deaths by age and cause from civil registration systems. The United Nations Population Division and the World Bank also compile data through their own channels.

Finally, UNICEF and other agencies of the UN IGME also compile child mortality data through regional workshops. Since 2008, about 100 countries have participated in these workshops, which provide technical assistance on demographic techniques and modeling methods and collect child mortality data that otherwise might be overlooked.

### Recalculation and Adjustment of Data

The sources of data described in Box 1 are often affected by known biases. Where this occurs, UN IGME recalculates or adjusts the data to allow for these biases. More information on these processes is given in other papers in this collection, but here we briefly consider three specific examples of recalculation and adjustment that were applied during the calculation of the latest child mortality estimates.

Full birth history (FBH) surveys (see Box 1) represent the largest source of data on child mortality for low- and middle-income countries, but they involve complex data collection and extensive interviewer training. Samples therefore tend to be quite small, typically 5,000–20,000 households. With a carefully designed sample, such numbers are adequate to produce national estimates, but do not permit extensive disaggregation. A paper in this collection by Pedersen and Liu shows that survey reports typically give estimates of child mortality measures for five-year periods before the survey, but UN IGME recently recalculated child mortality estimates from such data from the Demographic and Health Surveys (DHS) program for periods defined in calendar years [4]. On the basis of sampling precision, estimates for single calendar years can often be used for periods shortly before the survey, with the interval width gradually increasing further in the past. The criterion used to determine the interval width is the coefficient of variation (a measure of sampling uncertainty) of the U5MR estimates, specifically, adjusting period length to keep the coefficient of variation below 10% [4]. Importantly, this variable period approach addresses “birth transference,” an issue often encountered in FBH surveys whereby births early in a recent window for which extra information is collected are shifted backwards out of the window [5]. Child mortality estimates from FBHs are typically calculated for periods up to 25 years before the survey date, even though such estimates are increasingly affected by selection bias because earlier recorded births increasingly represent those to mothers young at the time of the birth who survived to the survey.

In another example, UN IGME adjusts the data used to estimate child mortality in eastern European countries because of concerns about the low levels of early neonatal death recorded in the civil registration systems of these countries compared to western European countries as a result of differences in the definition of live births [6]. In a regression analysis [7] of the ratio of early neonatal (under seven days) deaths to total neonatal deaths, UN IGME found that this ratio was significantly below the western European average of 0.77 for ten eastern European countries (Belarus, Bulgaria, Czech Republic, Estonia, Hungary, Latvia, Lithuania, Romania, Russian Federation, and Slovakia) and for Spain and Greece, where the ratio is less than 0.70. Overall, eastern European countries, Spain, and Greece fall into two strata based on the adjustment to the U5MR required to match their average ratio of early to total neonatal deaths to the western European average. Consequently, UN IGME applies a 10% upward adjustment to the U5MR for Belarus, Hungary, and Lithuania, and a 20% upward adjustment for the other countries, including Spain and Greece. Importantly, Estonia adopted the WHO definition of a live birth in 1992, and the civil registration data from Estonia show no sign of incompleteness after this adoption, so the adjustment of 20% is made to data from Estonia only prior to 1992. The remaining countries show no time trends in the incompleteness of early neonatal death data.

Finally, UN IGME also recalculates FBH child mortality estimates for countries with generalized HIV epidemics (defined for the purposes of CME as an HIV prevalence at any point in time since 1980 exceeding 5%). The assumption underlying the estimation of child mortality (whether directly or indirectly) from reports of mothers is that there is no correlation between the mortality risks of mothers and their children. HIV clearly breaks this assumption: both mothers and their children have higher risk, leading to a downward selection bias in child mortality estimates. As described by Walker and colleagues in a paper in this collection [8], in the latest UN IGME child mortality estimates, this bias is estimated by projecting both children and mothers forward from birth to a given survey date, separating mothers into HIV-negative and HIV-positive streams, and births into HIV-negative children born to HIV-negative mothers, HIV-negative children born to HIV-positive mothers, and HIV-positive children born to HIV-positive mothers; bias is estimated on the basis of child deaths that would not be reported because the mother has died.

### Inclusion and Exclusion of Observations

In calculating child mortality estimates, UN IGME also has to consider which observations should be included in its calculations and which should be excluded. An almost universal feature of indirect data from one type of data source, summary birth histories (see Box 1), is that estimates based on reports of women aged 15–19 years and to a lesser extent 20–24 years are higher than would be expected on the basis of the pattern of estimates for women in age groups from 25–29 years through 45–49 years. The high estimates based on reports of women in these younger age groups are largely the result of selection bias: first, a high proportion of the children ever born by these women are high-risk first births; and second, the women who start childbearing early are likely to come from socially and economically disadvantaged population groups with higher than average mortality risks. The UN IGME curve fitting approach therefore excludes indirect estimates based on reports of women aged 15–19 or 20–24 years.

FBHs provide the necessary data to make both direct (life table) and indirect estimates of childhood mortality. Historically, UN IGME has included both the direct and indirect estimates in its curve fitting approach. However, as explained in detail in another paper in this collection by Silva, a recent analysis suggests that indirect estimates from FBH data are systematically higher than the corresponding direct estimates [9]; thus, UN IGME is planning to only include direct estimates from FBH surveys in its analysis.

In some cases, entire surveys appear to have been adversely affected by non-sampling errors, and all the observations from such surveys are consequently excluded from the fitting process. As a rule of thumb, any survey in which all the observations lie below (or in rare cases above) all the observations from other surveys for corresponding time points is excluded from analysis. For a very small number of countries, the UN IGME partners consider the estimates from several surveys to lack face validity given what is known about the country, in which case data from those surveys are also excluded. Such countries have generally experienced a lengthy period of civil unrest or war, with adverse effects both on child mortality and on high-quality data collection.

### Deriving Smoothed Time Series of Infant Mortality and Under-Five Mortality

As a result of both sampling and non-sampling errors, estimates of child mortality vary from observation to observation within and between data series. Some form of smoothing is therefore required. A fitted smoothing function also allows for predicting for years without observations, for example, backwards in time from the earliest observation (backcasting) and forward in time from the most recent observation (forecasting).

The indicator most commonly used in the smoothing process is U5MR. Use of this indicator has two main advantages for countries lacking complete civil registration systems. First, direct estimates of the IMR from FBHs are often distorted by a widely observed tendency to report an excessive number of child deaths as occurring at “12 months” or “1 year.” Since some of these deaths almost certainly occurred before 12 months, the IMR is biased downwards, and the probability of dying between age 1 and age 5 (_4_ *q*_1_, the child mortality rate) is biased upwards. There is no comparable error around age 5, at which age deaths are rare, so U5MR is not biased. Second, the sensitivity of indirect estimates of U5MR from summary birth histories to an incorrect choice of underlying age pattern of child mortality is much smaller than that of indirect estimates of IMR (indeed, the proportion dead of children ever born by women aged 30–34 years is a remarkably good indicator of U5MR some six years before the survey under almost all patterns of fertility and mortality). Thus, for data from both FBHs and summary birth histories, U5MR is estimated more robustly than is IMR. Another article in this collection by Guillot and colleagues examines what we know about age patterns of child mortality in low- and middle-income countries, and investigates how well existing model life tables capture such patterns [10].

In countries with complete civil registration, the time series of estimates of IMR, calculated as infant deaths in a year divided by live births in that year, is often more complete than the time series of estimates of U5MR. Also, the IMR series makes no reliance on population age distributions, which may not be known with accuracy on a year-to-year basis. For such countries, the IMR and U5MR are smoothed independently.

In instances in which the smoothed time series is of U5MR, the IMR is by default obtained from U5MR using one of a number of families of model life tables [11]–[13]. The appropriate model is selected on the basis of observed relationships between the IMR and child mortality rate [14].

Importantly, the method used to derive smoothed time series of infant mortality and under-five mortality depends on whether the country under consideration is affected by an HIV/AIDS epidemic. For countries without a substantial generalized HIV/AIDS epidemic, UN IGME uses a loess (locally weighted least squares) smoother to obtain a “best estimate” trend line from diverse observations. The loess smoother fits a set of local regressions of the mortality indicator against the reference date of the estimates. A separate regression is fitted centered on each point of the data series, with neighboring points close to the index year being given more weight, and points further away progressively less weight. UN IGME uses a log-linear model of mortality change over time on the grounds that rates of change are likely to be more stable than absolute changes. The amount of smoothing applied to the data can be varied by changing the “band width,” α, which determines the range or window of points included in each local regression. A small α uses a small proportion of the available points in each regression, whereas a large α will potentially use all available points, though differentially weighted. The basic equation is:
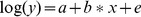
(1)where *y* is the mortality indicator (U5MR or IMR), *x* is calendar time, *e* is an error term (normally distributed), and *a* and *b* are the intercept and slope of the regression line, respectively.

**Figure 1:**
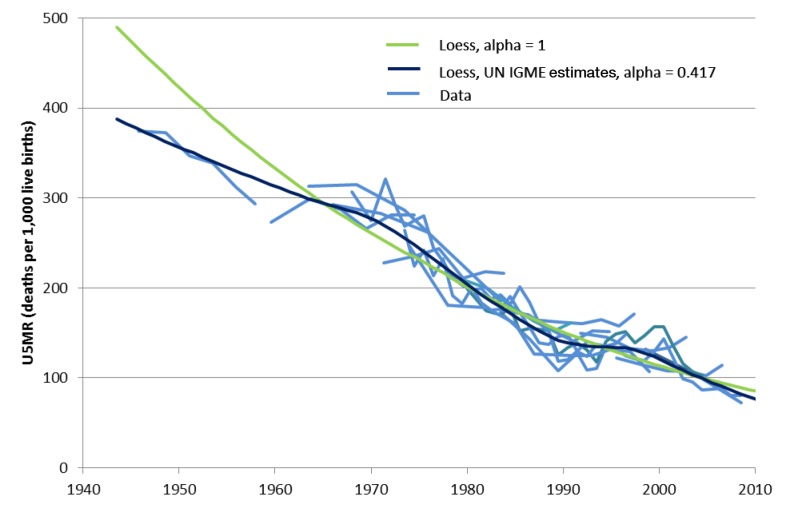
Illustration of the loess fitting procedure. Senegal is a country that has experienced substantial fluctuations in the rate of change of under-five mortality over the last 40 years. Two loess fitted trend lines are shown, one with α = 1, and the other the UN IGME 2011 trend line where α was defined using the standard α calculation. Generally, the greater the α value used in the loess fitting procedure, the more longer-term trends in the data influence the final trend line.

The weighting function *w* is tuned by the parameter α as follows. Let *d* be the absolute difference between the year of interest (*x*_0_) and year *x*: *d* = ∥*x*_0_−*x*∥. (a) For α<1, use a subset of 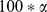 percent of observations closest to *x*_0_, with *w* = {1−[*d*_α_/max(*d*_α_)]^3^}^3^, where *d*_α_ is the absolute difference for each observation. (b) For α≥1, use all observations, and 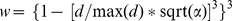. Figure 1 illustrates how α and the weighting function together determine the degree of smoothing.

On the basis of a review of the goodness of fit and plausibility of time trends, UN IGME sets α on the basis of the number of data series available for a given country, following the rule that α = 5/*n*, where *n* is the number of data series (e.g., the number of DHS surveys and other surveys available). Since a small α allows greater flexibility in the final curve, this has the effect of allowing more flexibility in the fitted curve in countries with a greater number of observations. For vital statistics data, UN IGME considers five years of data as equivalent to one survey. If only vital registration data are used in the regression for a country, α is generally set as 12.5/(number of data points), but for small countries with populations below 800,000, α is set as 25/(number of data points) to counter the larger random variation in data from these countries.

Countries with generalized HIV/AIDS epidemics pose two special problems that require a different approach to deriving smoothed time series for CME. First, estimates of child mortality based on reports of women about the survival of their children (whether in the form of full or summary birth histories) will be affected in a mature epidemic by selection bias. To deal with this problem, UN IGME adjusts direct estimates of U5MR for this bias [6]. Second, a population substantially affected by HIV/AIDS can experience sudden changes in child mortality. As an epidemic spreads, child mortality is likely to rise steeply, but as effective prevention of mother-to-child transmission is introduced, child mortality can fall dramatically within a year or so. No smoothing method can capture these sudden shifts, so UN IGME has adopted an alternative smoothing approach for such countries. The full description of this approach is provided in Text S1, but, briefly, once estimates of U5MR have been adjusted for selection bias, Joint United Nations Programme on HIV/AIDS (UNAIDS) estimates of under-five deaths due to AIDS are subtracted from total child deaths to obtain non-AIDS U5MR. The standard loess smoothing approach described above is applied to the non-AIDS U5MR series before adding the UNAIDS estimates of under-five deaths due to AIDS back to the series to estimate overall U5MR. Since the UNAIDS estimates take into account prevention of mother-to-child transmission, the sharp changes expected in U5MR can thus be captured (to the extent that the UNAIDS estimates are themselves correct).

### Generating Estimates of Neonatal Mortality

To estimate national time series of NMR and neonatal deaths, UN IGME has developed a multilevel statistical model [3]. Briefly, the sources of data for NMR are the same as for U5MR and IMR. For modeling purposes, a database is constructed consisting of observed pairs of NMR and U5MR. For a given year, NMR and U5MR are included in the database when data for both indicators are available. To ensure consistency with smoothed U5MR estimates, observed U5MR and NMR data points are rescaled for all years to match the smoothed U5MR estimates. For the 12 European countries with identified lower neonatal deaths registered in civil registration (see “Recalculation and Adjustment of Data” above), UN IGME adjusts neonatal mortality data going into the multilevel model by assuming that underreporting occurred solely in the neonatal age group. The multilevel model is:
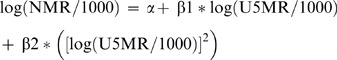
(2)with random effects parameters for level regression parameters at country level. Beyond taking into account the level of U5MR and regional patterns, no explicit adjustment is made for the NMR in countries with generalized HIV epidemics.

For countries with high-quality civil registration data for neonatal deaths, defined as (a) 100% complete registration for adult deaths and only civil registration data used for CME; (b) population greater than 800,000; and (c) with at least three civil registration data points for all the calendar windows 1990–1994, 1995–1999, 2000–2004, and 2005 onwards, UN IGME uses the same basic equation, but with random effects parameters for both level and trend regression parameters at country level [3].

### Other Aspects of Child Mortality Estimation

To calculate the absolute number of deaths among infants and children under five in a given year and country, UN IGME uses an abridged life table approach (see Text S2). To obtain the number of neonatal deaths, estimated NMRs are applied to numbers of live births from *World Population Prospects: The 2010 Revision* [15].

UN IGME has not in the past provided estimates of child mortality indicators by sex. However, another article in this collection by Sawyer examines male-to-female ratios of infant, child (ages one to four years), and under-five mortality by overall level of mortality and by world region [16]. In future updates of the UN IGME estimates, these ratios will be used to provide sex-specific estimates of such indicators.

Last but not least, a joint WHO/UNICEF country consultation is routinely undertaken to give the Ministry of Health in each country the opportunity to review all data inputs and the draft estimates for its country. The objectives of the country consultation are to identify relevant data not included in CME Info and to allow countries to review and provide feedback on estimates; it is not a country clearance process. In August 2011, during the preparation of the latest estimates [2], 92 out of 195 countries showed interest and received the preliminary estimates. Sixty-six countries provided comments or data, and as a consequence, estimates were revised for 21 countries using new data for 20 countries.

## Key Findings from the Latest UN IGME Child Mortality Estimates

Table 2 shows the latest (2011) UN IGME estimates for NMR, IMR, and U5MR for all the MDG regions for the years 1990, 2000, and 2010 [2]. Full annual time series from as early as 1960 to 2010 for each country are available via CME Info. Here we focus on results for the period 1990–2010.

**Table 2:** Estimates of under-five, infant, and neonatal mortality rates, and of acceleration in progress by MDG region [2].

Measure	Region	Year			Annual Rate of Reduction (ARR)			Acceleration
		1990	2000	2010	1990–2000 (Percent)	2000–2010 (Percent)	1990–2010 (Percent)	
**U5MR**	**Developed regions**	14.7	9.8	6.8	4.1	3.7	3.9	−9.8
	**Developing regions**	96.9	79.8	62.7	1.9	2.4	2.2	26.3
	Northern Africa	82.1	46.9	26.6	5.6	5.7	5.6	1.8
	Sub-Saharan Africa	173.9	154.3	121.0	1.2	2.4	1.8	100.0
	Latin America and the Caribbean	53.9	34.5	23.4	4.5	3.9	4.2	−13.3
	Caucasus and central Asia	77.4	62.3	45.3	2.2	3.2	2.7	45.5
	Eastern Asia	47.6	32.9	18.3	3.7	5.9	4.8	59.5
	Southern Asia	117.4	87.2	65.5	3.0	2.9	2.9	−3.3
	Southeastern Asia	71.5	48.4	32.2	3.9	4.1	4.0	5.1
	Western Asia	66.6	45.2	32.2	3.9	3.4	3.6	−12.8
	Oceania	74.6	63.1	52.2	1.7	1.9	1.8	11.8
	**World**	87.6	72.8	56.7	1.9	2.5	2.2	31.6
**IMR**	**Developed regions**	12.1	8.1	5.7	4.0	3.5	3.8	−12.5
	**Developing regions**	67.1	55.7	44.3	1.9	2.3	2.1	21.1
	Northern Africa	62.0	38.2	22.6	4.8	5.2	5.0	8.3
	Sub-Saharan Africa	104.8	94.1	76.2	1.1	2.1	1.6	90.9
	Latin America and the Caribbean	42.6	28.5	18.1	4.0	4.5	4.3	12.5
	Caucasus and central Asia	62.8	51.8	38.6	1.9	2.9	2.4	52.6
	Eastern Asia	37.6	27.1	15.7	3.3	5.5	4.4	66.7
	Southern Asia	84.0	64.7	50.7	2.6	2.4	2.5	−7.7
	Southeastern Asia	49.2	35.7	25.3	3.2	3.4	3.3	6.2
	Western Asia	52.3	35.2	25.4	4.0	3.3	3.6	−17.5
	Oceania	55.1	47.8	40.5	1.4	1.7	1.5	21.4
	**World**	60.9	50.9	40.2	1.8	2.4	2.1	33.3
**NMR**	**Developed regions**	7.0	5.0	3.7	3.4	3.0	3.2	−11.8
	**Developing regions**	35.6	30.7	25.1	1.5	2.0	1.7	33.3
	Northern Africa	29.1	19.9	13.0	3.8	4.3	4.0	13.2
	Sub-Saharan Africa	42.9	40.5	35.0	0.6	1.5	1.0	150.0
	Latin America and the Caribbean	22.7	16.2	10.8	3.4	4.1	3.7	20.6
	Caucasus and central Asia	30.2	25.8	20.7	1.6	2.2	1.9	37.5
	Eastern Asia	23.1	17.5	10.8	2.8	4.8	3.8	71.4
	Southern Asia	47.7	39.7	32.4	1.8	2.0	1.9	11.1
	Southeastern Asia	27.6	20.8	15.4	2.8	3.0	2.9	7.1
	Western Asia	28.1	21.3	16.3	2.8	2.7	2.7	−3.6
	Oceania	26.2	23.4	20.6	1.1	1.3	1.2	18.2
	**World**	32.5	28.1	22.8	1.5	2.1	1.8	40.0

Acceleration is calculated as the percent change in annual rate of reduction (ARR) from 1990–2000 to 2000–2010. See [22] for listings of countries by MDG region.

### Mortality Rates

**Figure 2:**
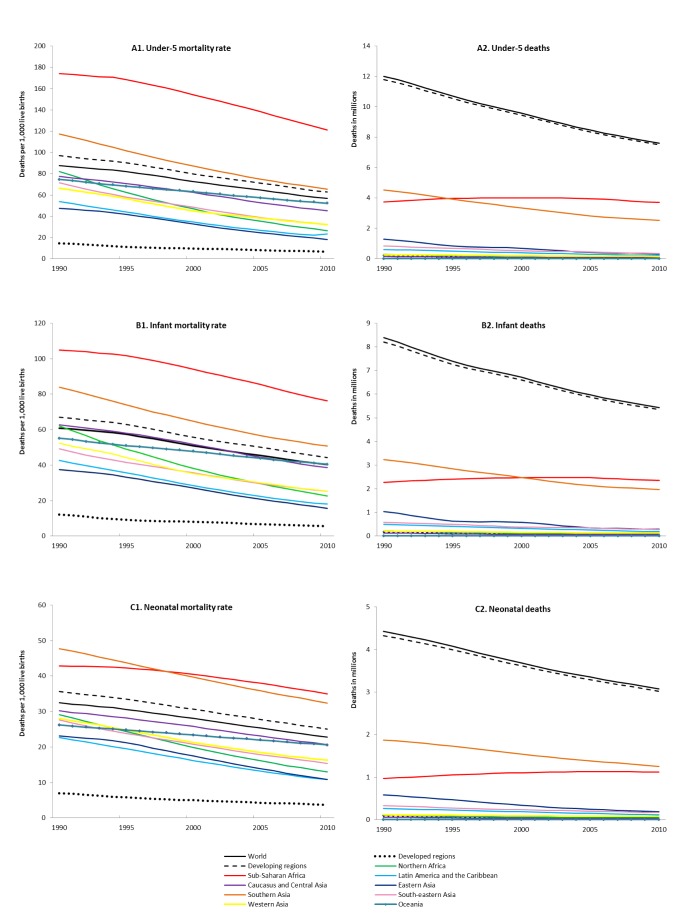
Global and regional under-five, infant, and neonatal mortality rates and deaths, 1990–2000. (A) U5MR and under-five deaths; (B) IMR and infant deaths; (C) NMR and neonatal deaths.

The global rates in all age components of under-five mortality have declined continuously over the last 20 years (Figure 2). The U5MR has declined by 35% from 87.6 deaths per 1,000 live births in 1990 to 56.7 in 2010, with an annual rate of reduction (ARR) of 2.2%; the IMR has fallen by 34% (an ARR of 2.1%), and the NMR has declined by 30% (an ARR of 1.8%).

All regions of the world have seen reductions in the U5MR (Figure 2). Five of nine developing regions show reductions of more than 50% between 1990 and 2010. Northern Africa has already achieved MDG 4, with a 68% reduction in the U5MR, and eastern Asia is close to doing so, with a 62% reduction; sub-Saharan Africa and Oceania have only achieved reductions of approximately 30% in U5MR, less than half that required to reach MDG 4. All regions have made less progress in reducing the NMR over the period 1990–2010 than in reducing the U5MR and IMR.

The highest rates of mortality in children under age five continue to occur in sub-Saharan Africa, where, in 2010, one in every eight children (121 per 1,000 live births) died before their fifth birthday. This level is nearly double the average in developing regions overall (62.7 per 1,000) and nearly 18 times the average for developed regions (6.8 per 1,000).

Southern Asia had the second highest U5MR in 2010—65.5 deaths per 1,000 live births, or about one in every 15 children. Oceania and Caucasus and central Asia are the only other regions with a U5MR over 40 deaths per 1,000 live births. Eastern Asia has the lowest U5MR among the developing regions (Table 2).

The global ARR in U5MR has accelerated from 1.9% a year over the period 1990–2000 to 2.5% a year over the period 2000–2010 (Table 2). The MDG regions with the greatest acceleration in the ARR of U5MR are eastern Asia and sub-Saharan Africa. Eastern Asia's ARR increased from 3.7% to 5.9% annually, while sub-Saharan Africa—combating the HIV/AIDS pandemic that has affected countries in the region more than elsewhere in the world—doubled its ARR from 1.2% a year over the period 1990–2000 to 2.4% a year over the period 2000–2010. By reaching lower mortality levels, western Asia, Latin America and the Caribbean, and the developed regions saw a slower ARR after 2000.

**Figure 3:**
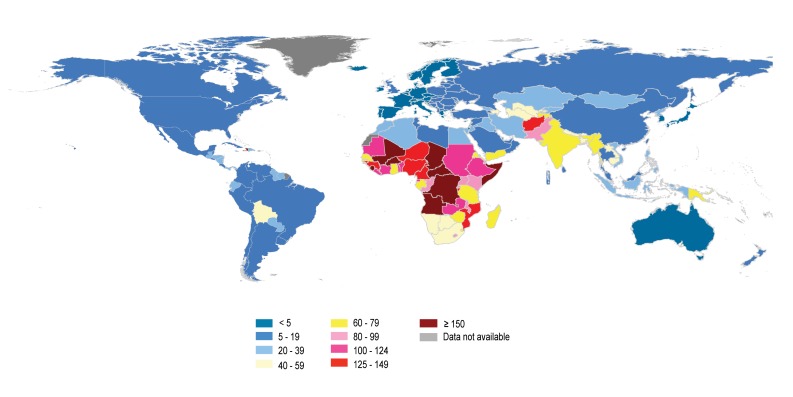
Under-five mortality rate by country for 2010. Data for Sudan refer to the country as it was constituted in 2010, before South Sudan seceded on 9 July 2011.

Figure 3 shows the U5MR in 2010 by country. Somalia, Mali, Burkina Faso, Sierra Leone, Chad, Democratic Republic of the Congo, Haiti, Angola, and Central African Republic have the highest U5MRs in the world—more than 150 deaths per 1,000 live births. In the developed regions, most countries have reached a level of U5MR below 10 per 1,000—only seven countries (Republic of Moldova, Albania, Romania, Ukraine, Bulgaria, the former Yugoslav Republic of Macedonia, and the Russian Federation) had a U5MR higher than 10 per 1,000.

### Number of Deaths

Substantial progress has been made over the last 20 years towards reducing the total number of deaths of children under age five, but the burden of child deaths is still unacceptably high. The estimated number of under-five deaths worldwide fell by 37% from 12.0 million in 1990 to 7.6 million in 2010 (Figure 2), but nearly 21,000 children are still dying each day. Over the same period, the annual number of infant deaths fell by 35% (8.4 million to 5.4 million), and the number of neonatal deaths fell by 31% (4.4 million to 3.1 million). Reflecting the slower decline in neonatal mortality relative to that of older ages, the proportion of under-five deaths that occur within the first month of life has increased globally from about 37% in 1990 to just over 40% in 2010, an increase observed in all MDG regions.

The distribution of the number of under-five deaths among regions over time reveals an increasing concentration of global under-five deaths in sub-Saharan Africa, reflecting slower declines in U5MR and continuing high fertility in this region compared to other regions. In 1990, sub-Saharan Africa accounted for 31% of global under-five deaths; by 2010, it accounted for 49% of these deaths. In contrast, southern Asia's share of global under-five deaths has fallen from around 38% in 1990 to 33% in 2010. Moreover, the number of deaths in southern Asia has almost halved, from 4.5 million to 2.5 million—by far the largest absolute reduction among all regions. Despite this decline, the region's share of global under-five deaths still remains the second highest, and sub-Saharan Africa and southern Asia together accounted for 82% of global under-five deaths in 2010. By contrast, the two regions accounted for less than 50% of the global population under the age of five.

Notably, the number of under-five deaths is concentrated in a few countries with large populations or very high mortality rates combined with high fertility. Seventy percent of the world's under-five deaths in 2010 occurred in only 15 countries, and about half occurred in only five countries: India, Nigeria, Democratic Republic of the Congo, Pakistan, and China. India and Nigeria together accounted for one-third of the total number of under-five deaths worldwide (22% and 11%, respectively). For neonatal deaths in 2010, India accounted for about 30% of deaths, and Nigeria for about 8%.

## Conclusions

Although substantial progress has been made over the last two decades in reducing U5MR, with increases in the rate of progress for many developing regions, these latest estimates of child mortality indicate that the rate of decline in the U5MR is still insufficient to achieve MDG 4 by 2015. Extrapolation of recent trends (specifically the ARR for the period 2000–2010) indicates that developing regions will not reach the MDG target until 2038, and will not reach the current U5MR of 6.8 seen in developed regions until 2103. At the country level, among the 66 countries with high U5MRs (at least 40 deaths per 1,000 live births in 2010), only seven have had an ARR of more than 4.4% (the minimum ARR required to reach MDG 4) between 1990 and 2010. It is notable that the ARR has been higher on average in countries with U5MRs below 40 per 1,000 than in those with U5MRs above 40.

These findings indicate that a more concerted effort is needed to increase further the pace of progress. Countries in sub-Saharan Africa and southern Asia in particular must give high priority to the reduction of child mortality. Progress must involve targeting the major killers of children. Liu et al. [17] estimate that 64% of deaths of children under five in 2010 were due to infectious diseases, with the largest shares being due to pneumonia (18%), diarrhea (11%), and malaria (7%). Effective interventions exist to prevent or treat all three conditions.

**Figure 4:**
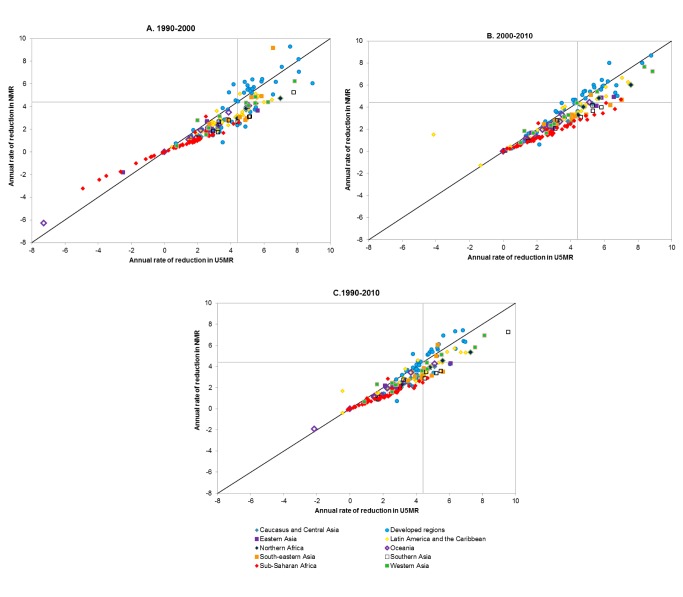
Annual rate of reduction in the under-five mortality rate compared to annual rate of reduction in the neonatal mortality rate over the periods 1990–2000, 2000–2010, and 1990–2010.

Although the acceleration in progress towards reducing the NMR (40% increase in ARR between the periods 1990–2000 and 2000–2010) has been greater than the acceleration in progress towards reducing the U5MR (32% increase) (Table 2), the rate of decline in NMR is slower than the rate of decline in U5MR in most countries (Figure 4). Accompanying the slower decline in NMR, the proportion of under-five deaths occurring in the neonatal period is increasing in every region and in almost all countries. Fully two-fifths of deaths under five occurred in the neonatal period in 2010, the most important individual causes being preterm birth complications (14% of under-five deaths), intrapartum-related events (9%), and sepsis/meningitis (5%) [17]. Systematic action is required by governments and partners to reach women and babies, particularly newborns, with effective care to accelerate the reduction in NMR.

At the global level, the estimates developed by UN IGME presented here are very similar to those published by the Institute for Health Metrics and Evaluation [18], though large differences exist for individual countries. The major reasons for these discrepancies are differences in data exclusion criteria and different approaches to allowing for known bias, as discussed in more detail in another article in this collection by Alkema and You [19]. It is essential, however, that discussions around different estimates and modeling strategies should not detract from the urgent actions required to address child mortality and morbidity.

Major improvements in data availability and advances in analytic methods over the last two decades have greatly expanded our knowledge of child mortality levels and trends in the developing world (Box 1; Table S1). However, the status quo is by no means satisfactory. For almost all of sub-Saharan Africa, estimates of U5MR are derived from household sample surveys and are therefore affected by known biases and sampling errors. Such estimates can never be timely. Thus, whereas the most recent observations used in our estimates for developed countries, which use data from civil registration systems, are typically for 2009, estimates for developing countries that rely on surveys are available for 2009 or later in only 20% of cases. Improved monitoring of child mortality trends requires the development of complete and accurate civil registration systems in developing countries. Moreover, countries that have suffered armed conflict are substantially overrepresented in the countries with the highest levels of U5MR, yet it is in these countries that data collection systems are weakest.

Finally, more analytic work on methodology is needed to improve the estimates of child mortality. Priority areas for such analytic work are as follows: to develop better methods to assess data quality and adjust for known bias, particularly underreporting; to understand mortality patterns due to conflicts, civil unrest, and natural disasters; and to generate appropriate estimates of uncertainty around estimates.

**Key Points**

- Monitoring progress towards Millennium Development Goals (MDGs) is a key interest of development agencies and countries.
- United Nations agencies have established a group—the UN Inter-agency Group for Child Mortality Estimation (UN IGME)—to coordinate the monitoring of progress on child mortality (MDG 4).
- This mechanism involves the compilation, adjustment, and smoothing of all nationally representative and relevant data.
- The methodologies adopted for this purpose are identified, reviewed, and recommended to the UN IGME by an independent Technical Advisory Group (TAG).
- The latest UN IGME estimates indicate that developing countries have made substantial progress in reducing under-five mortality since 1990, and that the pace of decline in under-five mortality has increased over the last decade; however, the decline has not been fast enough at the global level to reach the MDG 4 target of a two-thirds reduction of the 1990 level of under-five mortality by 2015.

## Supporting Information

Table S1 **Details of data and methods for each country.**
(PDF)

Text S1 **Details of loess regression in countries with generalized HIV/AIDS epidemics.**
(PDF)

Text S2 **Calculating numbers of infant and under-five deaths.**
(PDF)

## Abbreviations

ARR: annual rate of reduction
CME: child mortality estimation
DHS: Demographic and Health Surveys
FBH: full birth history
IMR: infant mortality rate
MDG: Millennium Development Goal
MDG 4: Millennium Development Goal 4
NMR: neonatal mortality rate
TAG: Technical Advisory Group of the United Nations Inter-agency Group for Child Mortality Estimation
U5MR: under-five mortality rate
UN IGME: United Nations Inter-agency Group for Child Mortality Estimation
UNAIDS: Joint United Nations Programme on HIV/AIDS
UNICEF: United Nations Children's Fund
WHO: World Health Organization
