# Supplementary material for: Child Mortality Estimation: Accelerated Progress in Reducing Global Child Mortality, 1990–2010
Source: PLoS Med. 2012 Aug 28;9(8):e1001303. doi: 10.1371/journal.pmed.1001303 (PMC3429379; doi:10.1371/journal.pmed.1001303)
Supplement: Table S1 — Details of data and methods for each country. (PDF) [file pmed.1001303.s001.pdf]

Table S1 Details of data and methods for each country used by the UN IGME in 2011

Part A: Details of data for each country

Country Name	U5MR Series	U5MR Included	IMR Series	IMR Included
Afghanistan	Afghanistan Mortality Survey (AMS) 2010_Direct	0	Afghanistan Mortality Survey (AMS) 2010_Direct	0
	Afghanistan Mortality Survey (AMS) 2010_Single year	0	Afghanistan Mortality Survey (AMS) 2010_Single year	0
	Demographic Health Survey 2007_Indirect	1	Demographic Health Survey 2007_Indirect	0
	National Risk and Vulnerability Assessment Survey 2007-08_Indirect	1	National Risk and Vulnerability Assessment Survey 2007-08_Indirect	0
	Multiple Indicator Cluster Survey 2003_Indirect	0	Multiple Indicator Cluster Survey 2003_Indirect	0
	Multiple Indicator Cluster Survey 2003_Direct	0	Multiple Indicator Cluster Survey 2003_Direct	0
	Multiple Indicator Cluster Survey 2000_Indirect	0	Multiple Indicator Cluster Survey 2000_Indirect	0
	Multiple Indicator Cluster Survey 1997_Indirect	1	Multiple Indicator Cluster Survey 1997_Indirect	0
	Census 1979_Direct	1	Census 1979_Direct	0
	National Demographic and Family Guidance Survey 1972_Single year	1	National Demographic and Family Guidance Survey 1973_Single year	0
	National Demographic and Family Guidance Survey 1972_Indirect	1	National Demographic and Family Guidance Survey 1972_Indirect	0
Albania	Demographic and Health Survey 2009_Indirect	1	Demographic and Health Survey 2009_Indirect	0
	Demographic and Health Survey (calendar year) 2009_Direct	1	Demographic and Health Survey (calendar year) 2009_Direct	0
	Multiple Indicator Cluster Survey 2006 _Indirect	1	Multiple Indicator Cluster Survey 2006 _Indirect	0
	Reproductive Health Survey-cdc 2002_Direct	1	Reproductive Health Survey-cdc 2002_Direct	0
	Multiple Indicator Cluster Survey 2000_Indirect	1	Multiple Indicator Cluster Survey 2000_Indirect	0
	WHO Vital Registration Data 2011 version_VR (Single year)	0	WHO Vital Registration Data 2011 version_VR (Single year)	0
Algeria	Multiple Indicator Cluster Survey 2006_Indirect	1	Multiple Indicator Cluster Survey 2006_Indirect	0
	Family Health Survey (PAPFAM) 2002_Direct	1	Family Health Survey (PAPFAM) 2002_Direct	0
	Multiple Indicator Cluster Survey 2000_Indirect	1	Multiple Indicator Cluster Survey 2000_Indirect	0
	Enquete nationale sur les objectifs 1995_Direct	1	Enquete nationale sur les objectifs 1995_Direct	0
	Enquete Algerienne sur la Sante de la Mere/Enfant 1992_Direct	1	Enquete Algerienne sur la Sante de la Mere/Enfant 1992_Direct	0
	Enquete Demographique 1970_Direct	1	Enquete Demographique 1970_Direct	0
	Enquete Fecondite 1970_Indirect	1	Enquete Fecondite 1970_Indirect	0
	Life Tables_Single year	1	Enquete Fecondite 1970_Single year	0
	WHO Vital Registration Data 2009 version_VR (Single year)	0	Vital Registraion from Demographie Algerienne 2007 and 2008_Single year	0
	Vital Registraion from Demographie Algerienne 2007, 2008 and 2009_VR	0	Life Tables_Single year	0
	(Single year)	0	Vital Registration_Single year	0
	WHO Vital Registration Data 2011 version_VR (Single year)		WHO Vital Registration Data 2009 version_VR (Single year)	0
			Vital Registraion from Demographie Algerienne 2007, 2008 and 2009_VR (Single	0
			year)	0
			WHO Vital Registration Data 2011 version_VR (Single year)	
Andorra	WHO Vital Registration Data 2011 version neighbouring_VR (Single year)	1	WHO Vital Registration Data 2011 version neighbouring_VR (Single year)	1

Angola	Household Incomes and Expenditures Survey IBEP 2008-2009_Indirect<br>Malaria Indicator Survey (MIS), 2006-07_Indirect<br>Multiple Indicator Cluster Survey 2001_Indirect<br>Multiple Indicator Cluster Survey 1996_Indirect	1<br>1<br>1<br>1	Household Incomes and Expenditures Survey IBEP 2008-2009_Indirect<br>Malaria Indicator Survey (MIS), 2006-07_Indirect<br>Multiple Indicator Cluster Survey 2001_Indirect<br>Multiple Indicator Cluster Survey 1996_Indirect	0<br>0<br>0<br>0
Antigua & Barbuda	WHO Vital Registration Data 2011 version_VR (Single year)	1	WHO Vital Registration Data 2011 version_VR (Single year)	1
Argentina	Census 2001_Indirect<br>Census 1991_Indirect<br>Census 1980_Indirect<br>Census 1970_Indirect<br>TABLAS DE VIDA NACIONALES ENERO-2009_Single year<br>WHO Good Vital Registration Data 2011 version_VR (Single year)	0<br>0<br>0<br>0<br>0<br>0	Census 2001_Indirect<br>Census 1991_Indirect<br>Census 1980_Indirect<br>Census 1970_Indirect<br>Estadisticas Vitales 2009_VR (Single year)<br>TABLAS DE VIDA NACIONALES ENERO-2009_Single year<br>WHO Good Vital Registration Data 2011 version_VR (Single year)	0<br>0<br>0<br>0<br>1<br>0<br>1
Armenia	Demographic and Health Survey (preliminary) 2010_Direct<br>Demographic and Health Survey 2005_Indirect<br>Demographic and Health Survey 2005_Direct<br>Demographic and Health Survey 2000_Indirect<br>Demographic and Health Survey 2000_Direct<br>Census 1989_Indirect<br>WHO Vital Registration Data 2011 version_VR (Single year)	1<br>1<br>1<br>1<br>1<br>0<br>0	Demographic and Health Survey (preliminary) 2010_Direct<br>Demographic and Health Survey 2005_Indirect<br>Demographic and Health Survey 2005_Direct<br>Demographic and Health Survey 2000_Indirect<br>Demographic and Health Survey 2000_Direct<br>Census 1989_Indirect<br>WHO Vital Registration Data 2011 version_VR (Single year)	0<br>0<br>0<br>0<br>0<br>0<br>0
Australia	WHO Good Vital Registration Data 2011 version_VR (Single year)	1	WHO Good Vital Registration Data 2011 version_VR (Single year)	1
Austria	WHO Good Vital Registration Data 2011 version_VR (Single year)	1	WHO Good Vital Registration Data 2011 version_VR (Single year)	1
Azerbaijan	Demographic and Health Survey 2006_Direct<br>Demographic and Health Survey 2006_Indirect<br>Reproductive Health Survey 2001_Direct<br>Multiple Indicator Cluster Survey 2000_Indirect<br>Census 1989_Indirect<br>WHO Vital Registration Data 2011 version_VR (Single year)	1<br>1<br>1<br>1<br>0<br>0	Demographic and Health Survey 2006_Direct<br>Demographic and Health Survey 2006_Indirect<br>Reproductive Health Survey 2001_Direct<br>Multiple Indicator Cluster Survey 2000_Indirect<br>Census 1989_Indirect<br>WHO Vital Registration Data 2011 version_VR (Single year)	0<br>0<br>0<br>0<br>0<br>0
Bahamas	WHO Vital Registration Data 2011 version_VR (Single year)	1	WHO Vital Registration Data 2011 version_VR (Single year)	1
Bahrain	Census 2001_Indirect<br>Family Health Survey 1995_Direct<br>Census 1991_Indirect<br>Child Health Survey 1989_Direct<br>Census 1981_Indirect<br>Census 1971_Indirect<br>Census 1965_Indirect<br>Child Health Survey 1989_Indirect<br>Family Health Survey 1995 _Indirect<br>WHO Vital Registration Data 2011 version_Single year	1<br>1<br>1<br>1<br>1<br>1<br>0<br>1<br>0<br>1	Census 2001_Indirect<br>Family Health Survey 1995_Direct<br>Census 1991_Indirect<br>Child Health Survey 1989_Direct<br>Census 1981_Indirect<br>Census 1971_Indirect<br>Vital Registration_Single year<br>Census 1965_Indirect<br>Child Health Survey 1989_Indirect<br>Family Health Survey 1995 _Indirect<br>WHO Vital Registration Data 2011 version_Single year	0<br>0<br>0<br>0<br>0<br>0<br>0<br>0<br>0<br>0<br>0

Bangladesh	Multiple Indicator Cluster Survey Preliminary 2009_Single year<br>Multiple Indicator Cluster Survey 2009_Indirect<br>Demographic and Health Survey 2007_Indirect<br>Demographic and Health Survey 2007_Direct<br>Demographic and Health Survey 2004_Direct<br>Demographic and Health Survey 2004_Indirect<br>Maternal Health Services and Maternal Mortality Survey 2001_Direct<br>Demographic and Health Survey 1999_Direct<br>Demographic and Health Survey 1999_Indirect<br>Demographic and Health Survey 1996_Direct<br>Demographic and Health Survey 1996_Indirect<br>Health and Demographic Survey 1994_Indirect<br>Demographic and Health Survey 1993_Direct<br>Demographic and Health Survey 1993_Indirect<br>Fertility Survey 1988-1989_Direct<br>Contraceptive Prevalence Survey 1985-1986_Indirect<br>Contraceptive Prevalence Survey 1983-1984_Indirect<br>Contraceptive Prevalence Survey 1981_Indirect<br>Contraceptive Prevalence Survey 1979-1980_Indirect<br>World Fertility Survey 1975-1976_Indirect<br>World Fertility Survey 1975-1976_Direct<br>Retrospective Fertility and Mortality Survey 1974_Indirect<br>Population Growth Estimation Experiment 1965_Direct<br>Bangladesh Demographic Survey and Vital Registration_Single year<br>National Life Tables_Single year<br>Vital Registration_Single year<br>SVR from Report on Sample Vital Registration System 2008_Single year	0<br>1<br>1<br>1<br>1<br>1<br>1<br>1<br>1<br>1<br>1<br>1<br>1<br>1<br>1<br>1<br>1<br>1<br>0<br>1<br>1<br>1<br>1<br>1<br>1<br>1<br>1	Multiple Indicator Cluster Survey Preliminary 2009_Single year<br>Multiple Indicator Cluster Survey 2009_Indirect<br>Demographic and Health Survey 2007_Indirect<br>Demographic and Health Survey 2007_Direct<br>Demographic and Health Survey 2004_Direct<br>Demographic and Health Survey 2004_Indirect<br>Maternal Health Services and Maternal Mortality Survey 2001_Direct<br>Demographic and Health Survey 1999_Direct<br>Demographic and Health Survey 1999_Indirect<br>Demographic and Health Survey 1996_Direct<br>Demographic and Health Survey 1996_Indirect<br>Health and Demographic Survey 1994_Indirect<br>Demographic and Health Survey 1993_Direct<br>Demographic and Health Survey 1993_Indirect<br>Contraceptive Prevalence Survey 1991_Single year<br>Contraceptive Prevalence Survey 1989_Direct<br>Fertility Survey 1988-1989_Direct<br>Contraceptive Prevalence Survey 1985-1986_Indirect<br>Contraceptive Prevalence Survey 1983-1984_Indirect<br>Contraceptive Prevalence Survey 1981_Indirect<br>Contraceptive Prevalence Survey 1979-1980_Indirect<br>World Fertility Survey 1975-1976_Indirect<br>World Fertility Survey 1975-1976_Direct<br>Retrospective Fertility and Mortality Survey 1974_Indirect<br>Population Growth Estimation Experiment 1965_Direct<br>Bangladesh Demographic Survey and Vital Registration_Single year<br>National Life Tables_Single year<br>Vital Registration_Single year<br>SVR from Report on Sample Vital Registration System 2008_Single year	0<br>0<br>0<br>0<br>0<br>0<br>0<br>0<br>0<br>0<br>0<br>0<br>0<br>0<br>0<br>0<br>0<br>0<br>0<br>0<br>0<br>0<br>0<br>0<br>0<br>0<br>0<br>0<br>0
Barbados	WHO Vital Registration Data 2011 version_VR (Single year)	1	WHO Vital Registration Data 2011 version_VR (Single year)	1
Belarus	Multiple Indicator Cluster Survey 2005_Indirect<br>Census 1999_Indirect<br>Census 1989_Indirect<br>WHO Good Vital Registration Data 2011 version_VR (Single year)<br>Vital Registration Data from Belstat 2011_VR (Single year)<br>WHO Good VR 2011 adjusted version by increasing 10%_VR (Single year)	0<br>0<br>0<br>0<br>0<br>1	Multiple Indicator Cluster Survey 2005_Indirect<br>Census 1999_Indirect<br>Census 1989_Indirect<br>WHO Good Vital Registration Data 2011 version_VR (Single year)<br>Vital Registration Data from Belstat 2011_VR (Single year)<br>WHO Good VR 2011 adjusted version by increasing 10%_VR (Single year)	0<br>0<br>0<br>0<br>0<br>1
Belgium	WHO Good Vital Registration Data 2011 version_VR (Single year)	1	WHO Good Vital Registration Data 2011 version_VR (Single year)	1
Belize	Multiple Indicator Cluster Survey 2006_Indirect<br>Census 2000_Indirect<br>Family Health Survey 1999_Indirect	1<br>1<br>1	Multiple Indicator Cluster Survey 2006_Indirect<br>Census 2000_Indirect<br>Family Health Survey 1999_Indirect	0<br>0<br>0

	Family Health Survey 1991_Indirect	1	Family Health Survey 1991_Indirect	0
	Vital Registration Data from MOH (Abstract of Statistics 2008)_VR (Single	0	US Statistics Division Vital Registration_Single year	0
	year)	0	Vital Registration Data from MOH (Abstract of Statistics 2008)_VR (Single year)	0
	WHO Vital Registration Data 2011 version_VR (Single year)		WHO Vital Registration Data 2011 version_VR (Single year)	0
Benin	Demographic and Health Survey 2006_Direct	1	Demographic and Health Survey 2006_Direct	0
	Demographic and Health Survey 2006_Indirect	1	Demographic and Health Survey 2006_Indirect	0
	Demographic and Health Survey 2001_Direct	1	Demographic and Health Survey 2001_Direct	0
	Demographic and Health Survey 2001_Indirect	1	Demographic and Health Survey 2001_Indirect	0
	Demographic and Health Survey 1996_Direct	1	Demographic and Health Survey 1996_Direct	0
	Demographic and Health Survey 1996_Indirect	1	Demographic and Health Survey 1996_Indirect	0
	Benin Fertility Survey 1982_Indirect	1	Benin Fertility Survey 1982_Indirect	0
	Benin Fertility Survey 1982_Direct	1	Benin Fertility Survey 1982_Direct	0
	Survey 1961_Indirect	1		
	Census 1992_Indirect	0		
Bhutan	Multiple Indicator Cluster Survey 2010_Indirect	1	Multiple Indicator Cluster Survey 2010_Indirect	0
	Census 2005_Indirect	1	Census 2005_Indirect	0
	Census 2005_Single year	1	Census 2005_Single year	0
	National Health Survey 2000_Direct	1	National Health Survey 2000_Direct	0
	National Health Survey 1994_Direct	1	National Health Survey 1994_Direct	0
	Demographic Sample Survey 1984_Indirect	1	Demographic Sample Survey 1984_Indirect	0
	Demographic Sample Survey 1984_Direct	1	Demographic Sample Survey 1984_Direct	0
Bolivia	Demographic and Health Survey 2008_Direct	1	Demographic and Health Survey 2008_Direct	0
	Demographic and Health Survey 2003_Direct	1	Demographic and Health Survey 2003_Direct	0
	Demographic and Health Survey 2003_Indirect	1	Demographic and Health Survey 2003_Indirect	0
	CENSO 2001_Indirect	0	CENSO 2001_Indirect	0
	Demographic and Health Survey 1998_Direct	1	Demographic and Health Survey 1998_Direct	0
	Demographic and Health Survey 1998_Indirect	1	Demographic and Health Survey 1998_Indirect	0
	Demographic and Health Survey 1994_Direct	1	Demographic and Health Survey 1994_Direct	0
	Demographic and Health Survey 1994_Indirect	1	Demographic and Health Survey 1994_Indirect	0
	CENSO 1992_Indirect	0	CENSO 1992_Indirect	0
	Demographic and Health Survey 1989_Direct	1	Demographic and Health Survey 1989_Direct	0
	Demographic and Health Survey 1989_Indirect	1	Demographic and Health Survey 1989_Indirect	0
	ENPV SUR ENERO 1988_Indirect	1	ENPV SUR ENERO 1988_Indirect	0
	EDEN SUR ENERO 1980_Indirect	1	EDEN SUR ENERO 1980_Indirect	0
	CENSO 1976_Indirect	1	CENSO 1976_Indirect	0
	EDEN SUR ENERO 1975_Indirect	1	EDEN SUR ENERO 1975_Indirect	0
Bosnia & Herzegovina	WHO Good Vital Registration Data 2011 version_VR (Single year)	1	WHO Good Vital Registration Data 2011 version_VR (Single year)	1
Botswana	Family Health Survey 2007-2008_Direct	1	Family Health Survey 2007-2008_Direct	0
	Demographic Survey 2006_Indirect	1	Demographic Survey 2006_Indirect	0

	Census 2000_Indirect	1	Census 2000_Indirect	0
	Multiple Indicator Cluster Survey 2000_Indirect	1	Multiple Indicator Cluster Survey 2000_Indirect	0
	Family Health Survey 1996_Direct	0	Family Health Survey 1996_Direct	0
	Census 1991_Indirect	1	Census 1991_Indirect	0
	Family Health Survey 1988_Indirect	1	Family Health Survey 1988_Indirect	0
	Family Health Survey 1988_Direct	0	Family Health Survey 1988_Direct	0
	Demographic and Health Survey 1988_Direct	1	Demographic and Health Survey 1988_Direct	0
	Family Health Survey 1984_Indirect	1	Family Health Survey 1984_Indirect	0
	Census 1981_Indirect	1	Census 1981_Indirect	0
	Census 1971_Indirect	1	Census 1971_Indirect	0
			Vital Registration_Single year	0
Brazil	Pesquisa Nacional por Amostra de Domicilios PNAD 2009_Indirect	1	Pesquisa Nacional por Amostra de Domicilios PNAD 2009_Indirect	0
	Pesquisa Nacional por Amostra de Domicilios PNAD 2008_Indirect	1	Pesquisa Nacional por Amostra de Domicilios PNAD 2008_Indirect	0
	PNAD Oeste ENERO 2007_Indirect	1	PNAD Oeste ENERO 2007_Indirect	0
	National HH Survey 2006_Indirect	1	National HH Survey 2006_Indirect	0
	PNAD Oeste ENERO 2005_Indirect	1	PNAD Oeste ENERO 2005_Indirect	0
	CENSO 2000_Indirect	1	CENSO 2000_Indirect	0
	Demographic and Health Survey 1996_Indirect	1	Demographic and Health Survey 1996_Indirect	0
	Demographic and Health Survey 1996_Direct	1	Demographic and Health Survey 1996_Direct	0
	Census 1991_Indirect	1	Census 1991_Indirect	0
	PNAD ENERO 1986_Direct	1	PNAD ENERO 1986_Direct	0
	National Health Survey 1986_Indirect	1	National Health Survey 1986_Indirect	0
	Demographic and Health Survey 1986_Indirect	1	Demographic and Health Survey 1986_Indirect	0
	National Health Survey 1984_ Indirect	1	Demographic and Health Survey 1986_Direct	0
	Census 1980_Indirect	1	National Health Survey 1984_ Indirect	0
	National Health Survey 1978_Indirect	1	Census 1980_Indirect	0
	National Health Survey 1977_Indirect	1	National Health Survey 1978_Indirect	0
	National Health Survey 1976_Indirect	1	National Health Survey 1977_Indirect	0
	National Health Survey 1973_Indirect	1	National Health Survey 1976_Indirect	0
	National Health Survey 1972_Indirect	1	National Health Survey 1973_Indirect	0
	Census 1970_Indirect	1	National Health Survey 1972_Indirect	0
	Vital Registration Data from Information System on Mortality of Brazil Enero	0	Census 1970_Indirect	0
	2009_VR (Single year)		Estadisticas Vitales Anuarios Estadisticos ENERO 2009_VR (Single year)	0
	WHO Vital Registration Data 2011 version_VR (Single year)	0	Vital Registration Data from Information System on Mortality of Brazil Enero	0
			2009_VR (Single year)	
			WHO Vital Registration Data 2011 version_VR (Single year)	0
Brunei	WHO Good Vital Registration Data 2011 version_VR (Single year)	1	WHO Good Vital Registration Data 2011 version_VR (Single year)	1
Bulgaria	WHO Good Vital Registration Data 2011 version_VR (Single year)	0	Vital Registration Data from National Center for Health Information_Single year	0
	WHO Good VR 2011 adjusted version by increasing 20%_VR (Single year)	1	WHO Good Vital Registration Data 2011 version_VR (Single year)	0
			WHO Good VR 2011 adjusted version by increasing 20%_VR (Single year)	1

Burkina Faso	Census 2006_Indirect	0	Multiple Indicator Cluster Survey 2006_Indirect	1
	Multiple Indicator Cluster Survey 2006_Indirect	1	RECENSEMENT GENERAL DE LA POPULATION ET DE LHABITATION DE (RGPH)	0
	RECENSEMENT GENERAL DE LA POPULATION ET DE LHABITATION DE (RGPH)	0	2006 _Single year	
	2006 _Single year		RECENSEMENT GENERAL DE LA POPULATION ET DE LHABITATION DE (RGPH)	0
	RECENSEMENT GENERAL DE LA POPULATION ET DE LHABITATION DE (RGPH)	0	2006_Indirect	
	2006_Indirect		Demographic and Health Survey 2003_Direct	1
	Demographic and Health Survey 2003_Direct	1	Demographic and Health Survey 2003_Indirect	1
	Demographic and Health Survey 2003_Indirect	1	Demographic and Health Survey 1999_Direct	1
	Demographic and Health Survey 1999_Direct	1	Demographic and Health Survey 1999_Indirect	1
	Demographic and Health Survey 1999_Indirect	1	Demographic and Health Survey 1992_Direct	1
	Demographic and Health Survey 1992_Direct	1	Demographic and Health Survey 1992_Indirect	1
	Demographic and Health Survey 1992_Indirect	1	Census 1985_Indirect	1
	Census 1985_Indirect	1	Post-Enumeration Survey 1976_Indirect	1
	Post-Enumeration Survey 1976_Indirect	1	Survey 1960-61_Indirect	1
	Survey 1960-61_Indirect	1	Survey 1991_Indirect	1
	Survey 1991_Indirect	1		
Burundi	Demographic Health Survey (Preliminary) 2010_Direct	1	Demographic Health Survey (Preliminary) 2010_Direct	0
	Multiple Indicator Cluster Survey 2005_Indirect	1	Multiple Indicator Cluster Survey 2005_Indirect	0
	Multiple Indicator Cluster Survey 2000_Indirect	1	Multiple Indicator Cluster Survey 2000_Indirect	0
	Census 1990_Indirect	1	Census 1990_Indirect	0
	Demographic and Health Survey 1987_Indirect	1	Demographic and Health Survey 1987_Indirect	0
	Demographic and Health Survey 1987_Direct	1	Demographic and Health Survey 1987_Direct	0
	Post Census Survey 1979_Indirect	1	Post Census Survey 1979_Indirect	0
	Demographic Survey 1970_Indirect	1	Demographic Survey 1970_Indirect	0
	Demographic Survey 1970_ Direct	1	Demographic Survey 1970_ Direct	0
Cambodia	Demographic and Health Survey (Preliminary) 2010_Direct	1	Demographic and Health Survey (Preliminary) 2010_Direct	0
	Demographic and Health Survey 2005_Direct	1	Census 2008_Indirect	0
	Demographic and Health Survey 2005_Indirect	1	Demographic and Health Survey 2005_Direct	0
	Demographic and Health Survey 2000_Direct	1	Demographic and Health Survey 2005_Indirect	0
	Demographic and Health Survey 2000_Indirect	1	Demographic and Health Survey 2000_Direct	0
	National Health Survey 1998_Indirect	1	Demographic and Health Survey 2000_Indirect	0
	National Health Survey 1998_ Direct	0	National Health Survey 1998_Indirect	0
	Census 1998_Indirect	1	National Health Survey 1998_ Direct	0
			Census 1998_Indirect	0
Cameroon	Demographic and Health Survey 2004_Indirect	1	Demographic and Health Survey 2004_Indirect	0
	Demographic and Health Survey 2004_Direct	1	Demographic and Health Survey 2004_Direct	0
	Multiple Indicator Cluster Survey 2000_Indirect	1	Multiple Indicator Cluster Survey 2000_Indirect	0
	Demographic and Health Survey 1998_Indirect	1	Demographic and Health Survey 1998_Indirect	0
	Demographic and Health Survey 1998_Direct	1	Demographic and Health Survey 1998_Direct	0
	Demographic and Health Survey 1991_Indirect	1	Demographic and Health Survey 1991_Indirect	0

	Demographic and Health Survey 1991_Direct	1	Demographic and Health Survey 1991_Direct	0
	World Fertility Survey 1978_Indirect	1	World Fertility Survey 1978_Indirect	0
	World Fertility Survey 1978_Direct	1	World Fertility Survey 1978_Direct	0
Canada	WHO Good Vital Registration Data 2011 version_VR (Single year)	1	WHO Good Vital Registration Data 2011 version_VR (Single year)	1
Cape Verde	Demographic and Health Survey 2005_Indirect	1	Demographic and Health Survey 2005_Indirect	0
	Demographic and Health Survey 2005_Direct	1	Demographic and Health Survey 2005_Direct	0
	Census 2000_Direct	1	Census 2000_Direct	0
	Reproductive Health Survey 1998_Indirect	1	Reproductive Health Survey 1998_Indirect	0
	Reproductive Health Survey 1998_ Direct	1	Reproductive Health Survey 1998_ Direct	0
	Census 1980_Direct	1	Census 1980_Direct	0
	Census 1970_Direct	1	Census 1970_Direct	0
	Vital Registration Data from Relatorio Estatistico 2007 2009 Ministry of	0	Vital Registration Data from Relatorio Estatistico 2007 2009 Ministry of	0
	Health_Single year		Health_Single year	0
	UNPD Vital Registration Data 2010 version_Single Year	0	UNPD Vital Registration Data 2010 version_Single Year	0
	Census 1960_Indirect	0	Census 1960_Indirect	0
	WHO Vital Registration Data 2011 version_VR (Single year)	0	WHO Vital Registration Data 2011 version_VR (Single year)	0
Central African Republic	Multiple Indicator Cluster Survey 2006_Indirect	1	Multiple Indicator Cluster Survey 2006_Indirect	0
	Multiple Indicator Cluster Survey 2000_Indirect	1	Multiple Indicator Cluster Survey 2000_Indirect	0
	Demographic and Health Survey 1994_Indirect	1	Demographic and Health Survey 1994_Indirect	0
	Demographic and Health Survey 1994_Direct	1	Demographic and Health Survey 1994_ Direct	0
	Census 1988_Indirect	1	Census 1988_Indirect	0
	Census 1975_Indirect	1	Census 1975_Indirect	0
Chad	Multiple Indicator Cluster Survey 2010_Indirect	1	Multiple Indicator Cluster Survey 2010_Indirect	0
	Demographic and Health Survey 2004_Indirect	1	Demographic and Health Survey 2004_Indirect	0
	Demographic and Health Survey 2004_Direct	1	Demographic and Health Survey 2004_Direct	0
	Multiple Indicator Cluster Survey 2000_Indirect	1	Multiple Indicator Cluster Survey 2000_Indirect	0
	Demographic and Health Survey 1997_Indirect	1	Demographic and Health Survey 1997_Indirect	0
	Demographic and Health Survey 1997_Direct	1	Demographic and Health Survey 1997_Direct	0
	Census 1993_Indirect	0	Census 1993_Indirect	0
Chile	CENSO 2002_Indirect	0	CENSO 2002_Indirect	0
	CENSO 1992_Indirect	0	CENSO 1992_Indirect	0
	CENSO 1982_Indirect	0	CENSO 1982_Indirect	0
	CENSO 1970_Indirect	0	CENSO 1970_Indirect	0
	TABLAS DE VIDA NACIONALES ENERO 2009_Single year	0	TABLAS DE VIDA NACIONALES ENERO 2009_Single year	0
	Vital Registration from Ministerio de Salud. Departamento de Estadisticas de	0	Vital Registration from Ministerio de Salud. Departamento de Estadisticas de	0
	Salud.Anexo Salud1_VR (Single year)		Salud.Anexo Salud1_VR (Single year)	
	WHO Vital Registration Data 2011 version_VR (Single year)	1	WHO Vital Registration Data 2011 version_VR (Single year)	1
China	Population 1% Sample Survey 2005_Direct	0	Population 1% Sample Survey 2005_Direct	0
	Population 1% Sample Survey 2005_Indirect	0	Population 1% Sample Survey 2005_Indirect	0
	Census 2000_Indirect	0	Census 2000_Indirect	0

	Population Sample Survey 1995 _Indirect	0	Population Sample Survey 1995 _Indirect	0
	Fertility Sampling Survey 1992_Direct	1	Fertility Sampling Survey 1992_Direct	0
	Census 1990_Indirect	0	Census 1990_Indirect	0
	National Survey on Fertility and Birth Control 1988_Single year	1	National Survey on Fertility and Birth Control 1988_Single year	0
	Population Sample Survey 1987_Indirect	1	Population Sample Survey 1987_Indirect	0
	Census 1982_Indirect	1	Census 1982_Indirect	0
	Population Sample Survey 1982_Indirect	1	Population Sample Survey 1982_Indirect	0
	Maternal and Child Mortality Surveillance System 2008 Version_Single year	1	Maternal and Child Mortality Surveillance System 2008 Version_Single year	0
	Adjusted Census Deaths_Single year	1	Adjusted Census Deaths_Single year	0
	Maternal and Child Mortality Surveillance System_Single year	0	Maternal and Child Mortality Surveillance System_Single year	0
	National Life Tables_Single year	0	National Life Tables_Single year	0
Colombia	Demographic and Health Survey 2010_Direct	1	Demographic and Health Survey 2010_Direct	1
	Demographic and Health Survey 2010_Indirect	1	Demographic and Health Survey 2010_Indirect	1
	Demographic and Health Survey 2005_Direct	1	Demographic and Health Survey 2005_Direct	1
	Demographic and Health Survey 2005_Indirect	1	Demographic and Health Survey 2005_Indirect	1
	Demographic and Health Survey 2000_Direct	1	Demographic and Health Survey 2000_Direct	1
	Demographic and Health Survey 2000_Indirect	1	Demographic and Health Survey 2000_Indirect	1
	Demographic and Health Survey 1995_Direct	1	Demographic and Health Survey 1995_Direct	0
	Demographic and Health Survey 1995_Indirect	1	Demographic and Health Survey 1995_Indirect	1
	Demographic and Health Survey 1990_Direct	1	Demographic and Health Survey 1990_Direct	1
	Demographic and Health Survey 1990_Indirect	1	Demographic and Health Survey 1990_Indirect	1
	Demographic and Health Survey 1986_Direct	1	Demographic and Health Survey 1986_Direct	1
	Demographic and Health Survey 1986_Indirect	1	Demographic and Health Survey 1986_Indirect	1
	Census 1985_Indirect	1	Census 1985_Indirect	1
	HOG 1980_Indirect	1	HOG 1980_Indirect	1
	HOG 1978_Indirect	1	HOG 1978_Indirect	1
	Contraceptive Prevalence Survey 1978_Single year	1	Contraceptive Prevalence Survey 1978_Single year	1
	World Fertility Survey 1976_Direct	1	World Fertility Survey 1976_Direct	1
	World Fertility Survey 1976_Indirect	1	World Fertility Survey 1976_Indirect	1
	Census 1973_Indirect	1	Census 1973_Indirect	1
	Vital Registration (Mexico March 2009)_Single year	0	Vital Registration (Mexico March 2009)_Single year	0
	WHO Vital Registration Data 2011 version_VR (Single year)	0	WHO Vital Registration Data 2011 version_VR (Single year)	0
Comoros	Census 2003_Single year	1	Census 2003_Single year	0
	Multiple Indicator Cluster Survey 2000_Indirect	0	Multiple Indicator Cluster Survey 2000_Indirect	0
	Demographic and Health Survey 1996_Indirect	1	Demographic and Health Survey 1996_Indirect	0
	Demographic and Health Survey 1996_Direct	1	Demographic and Health Survey 1996_Direct	0
	Census 1991_Single year	1	Census 1991_Single year	0
	Census 1980_Single year	1	Census 1980_Single year	0
	Census 1980_Indirect	0	Census 1980_Indirect	0
Congo	Demographic and Health Survey 2005_Indirect	1	Demographic and Health Survey 2005_Indirect	0

	Demographic and Health Survey 2005_Direct	1	Demographic and Health Survey 2005_Direct	0
	Census 1974_Indirect	1	Census 1974_Indirect	0
Congo DR	Multiple Indicator Cluster Survey 2010_Indirect	1	Multiple Indicator Cluster Survey 2010_Indirect	0
	Demographic and Health Survey 2007_Indirect	1	Demographic and Health Survey 2007_Indirect	0
	Demographic and Health Survey 2007_Direct	1	Demographic and Health Survey 2007_Direct	0
	Multiple Indicator Cluster Survey 2001_Indirect	1	Multiple Indicator Cluster Survey 2001_Indirect	0
	Multiple Indicator Cluster Survey 1995_Indirect	1	Multiple Indicator Cluster Survey 1995_Indirect	0
	Census 1984_Indirect	1	Census 1984_Indirect	0
Cook Islands	Census 2001_Indirect	1	Census 2001_Indirect	0
	Census 1996_Indirect	1	Census 1996_Indirect	0
	Census 1981_Indirect	1	Census 1981_Indirect	0
	Census 1976_Indirect	0	Census 1976_Indirect	0
	Census 1966_Indirect	1	Census 1966_Indirect	0
	WHO Vital Registration Data 2011 version_VR (Single year)	0	Vital Registration from Statistics Office_VR (Single year)	0
	WHO Vital Registration Data 2011 version Moving Average_VR (Single year)	1	WHO Vital Registration Data 2011 version_VR (Single year)	0
			WHO Vital Registration Data 2011 version Moving Average_VR (Single year)	0
Costa Rica	Census 2000_Indirect	0	Census 2000_Indirect	0
	Encuesta de Fecundidad y Salud 1986_Indirect	0	Encuesta de Fecundidad y Salud 1986_Indirect	0
	Census 1984_Indirect	0	Encuesta de Fecundidad y Salud 1986_Direct	0
	Contraceptive Prevalence Survey 1981_Indirect	0	Census 1984_Indirect	0
	Contraceptive Prevalence Survey 1978_Indirect	0	Contraceptive Prevalence Survey 1981_Direct	0
	National Fertility Survey 1976_Direct	0	Contraceptive Prevalence Survey 1981_Indirect	0
	National Fertility Survey 1976_Indirect	0	Contraceptive Prevalence Survey 1978_Indirect	0
	Censo 1973_Indirect	0	National Fertility Survey 1976_Direct	0
	Vital Registration (Mexico March 26, Roberto)_VR (Single year)	0	National Fertility Survey 1976_Indirect	0
	TABLAS DE VIDA NACIONALES ENERO 2009_Single year	0	Censo 1973_Indirect	0
	WHO Vital Registration Data 2011 version_Single year	1	Vital Registration ENERO 2009	0
			Estadisticas Vitales INFANT Enviada por INEC en ENERO 2009_Single year	0
			Vital Registration (Mexico March 26, Roberto)_VR (Single year)	0
			TABLAS DE VIDA NACIONALES ENERO 2009_Single year	0
			WHO Vital Registration Data 2011 version_Single year	1
Cote d Ivoire	AIDS Indicator Survey 2005_Indirect	1	AIDS Indicator Survey 2005_Indirect	0
	AIDS Indicator Survey 2005_Direct	0	AIDS Indicator Survey 2005_Direct	0
	Demographic and Health Survey 2005_Direct	1	Demographic and Health Survey 2005_Direct	0
	Demographic and Health Survey 1998_Indirect	1	Demographic and Health Survey 1998_Indirect	0
	Demographic and Health Survey 1998_Direct	1	Demographic and Health Survey 1998_Direct	0
	Demographic and Health Survey 1994_Indirect	1	Demographic and Health Survey 1994_Indirect	0
	Demographic and Health Survey 1994_Direct	1	Demographic and Health Survey 1994_Direct	0
	World Fertility Survey 1980_Indirect	1	World Fertility Survey 1980_Indirect	0
	World Fertility Survey 1980_Direct	1	World Fertility Survey 1980_Direct	0

	Demographic Survey Repeated Passages 1978_Indirect<br>Demographic Survey Repeated Passages 1978_Direct<br>Census 1988_Indirect<br>Survey 1957-58_Indirect	1<br>1<br>0<br>0	Demographic Survey Repeated Passages 1978_Indirect<br>Demographic Survey Repeated Passages 1978_Direct<br>Census 1988_Indirect<br>Survey 1957-58_Indirect	0<br>0<br>0<br>0
Croatia	WHO Good Vital Registration Data 2011 version_VR (Single year)	1	WHO Good Vital Registration Data 2011 version_VR (Single year)	1
Cuba	National Fertility Survey 1987_Indirect<br>Census 1981_Indirect<br>National Demographic Survey 1979_Indirect<br>National Population Survey on Income and Expenditures 1974_Indirect<br>National Life Tables_Single year<br>TABLAS DE VIDA NACIONALES ENERO 2009_Single year<br>WHO Good Vital Registration Data 2011 version_VR (Single year)	0<br>0<br>0<br>0<br>0<br>0<br>1	National Fertility Survey 1987_Indirect<br>Census 1981_Indirect<br>National Demographic Survey 1979_Indirect<br>National Population Survey on Income and Expenditures 1974_Indirect<br>National Life Tables_Single year<br>TABLAS DE VIDA NACIONALES ENERO 2009_Single year<br>WHO Good Vital Registration Data 2011 version_VR (Single year)	0<br>0<br>0<br>0<br>0<br>0<br>1
Cyprus	WHO Good Vital Registration Data 2011 version_VR (Single year)	1	WHO Good Vital Registration Data 2011 version_VR (Single year)	1
Czech Republic	WHO Good Vital Registration Data 2011 version_VR (Single year)<br>WHO Good VR 2011 adjusted version by increasing 20%_VR (Single year)	0<br>1	WHO Good Vital Registration Data 2011 version_VR (Single year)<br>WHO Good VR 2011 adjusted version by increasing 20%_VR (Single year)	0<br>1
Denmark	WHO Good Vital Registration Data 2011 version_VR (Single year)	1	WHO Good Vital Registration Data 2011 version_VR (Single year)	1
Djibouti	Multiple Indicator Cluster Survey 2006_Indirect<br>Family Health Survey (PAPFAM) 2002_Direct<br>Demographic Survey 1991_Indirect	1<br>1<br>1	Multiple Indicator Cluster Survey 2006_Indirect<br>Family Health Survey (PAPFAM) 2002_Direct<br>Demographic Survey 1991_Indirect	0<br>0<br>0
Dominica	WHO Good Vital Registration Data 2011 version_VR (Single year)	1	WHO Good Vital Registration Data 2011 version_VR (Single year)	1
Dominican Republic	Demographic and Health Survey 2007_Indirect<br>Demographic and Health Survey 2007_Direct<br>Multiple Indicator Cluster Survey 2006_Direct<br>Demographic and Health Survey 2002_Indirect<br>Demographic and Health Survey 2002_Direct<br>Censo 2002_Indirect<br>Demographic and Health Survey 1999_Direct<br>Demographic and Health Survey 1996_Indirect<br>Demographic and Health Survey 1996_Direct<br>Demographic and Health Survey 1991_Indirect<br>Demographic and Health Survey 1991_Direct<br>Demographic and Health Survey 1986_Indirect<br>Demographic and Health Survey 1986_Direct<br>Contraceptive Prevalence Survey 1983_Indirect<br>Census 1981_Single year<br>World Fertility Survey 1980_Direct<br>World Fertility Survey 1980_Indirect<br>World Fertility Survey 1975_Direct<br>World Fertility Survey 1975_Indirect<br>Census 1970_Single year	1<br>1<br>1<br>1<br>1<br>0<br>0<br>1<br>1<br>1<br>1<br>1<br>1<br>1<br>1<br>1<br>1<br>1<br>1<br>1	Demographic and Health Survey 2007_Indirect<br>Demographic and Health Survey 2007_Direct<br>Encuesta de Hogar Mitch 2006_Indirect<br>Multiple Indicator Cluster Survey 2006_Direct<br>Demographic and Health Survey 2002_Indirect<br>Demographic and Health Survey 2002_Direct<br>Censo 2002_Indirect<br>Demographic and Health Survey 1999_Direct<br>Demographic and Health Survey 1996_Indirect<br>Demographic and Health Survey 1996_Direct<br>Demographic and Health Survey 1991_Indirect<br>Demographic and Health Survey 1991_Direct<br>Demographic and Health Survey 1986_Indirect<br>Demographic and Health Survey 1986_Direct<br>Contraceptive Prevalence Survey 1983_Indirect<br>Census 1981_Single year<br>World Fertility Survey 1980_Direct<br>World Fertility Survey 1980_Indirect<br>World Fertility Survey 1975_Direct<br>World Fertility Survey 1975_Indirect	0<br>0<br>0<br>0<br>0<br>0<br>0<br>0<br>0<br>0<br>0<br>0<br>0<br>0<br>0<br>0<br>0<br>0<br>0<br>0

	WHO Vital Registration Data 2011 version_VR (Single year)	0	Census 1970_Single year	0
			Est. Vitales Anuarios y datos enviados por la ONE Enero 2009_Single year	0
			WHO Vital Registration Data 2011 version_VR (Single year)	0
Ecuador	ENDEMAIN 2004_Direct	1	ENDEMAIN 2004_Direct	1
	CENSO 2001_Indirect	1	CENSO 2001_Indirect	1
	ENDEMAIN 1999_Direct	1	ENDEMAIN 1999_Direct	1
	ENDEMAIN 1994_Direct	1	ENDEMAIN 1994_Direct	1
	Census 1990_Indirect	1	Census 1990_Indirect	1
	Encuesta Demografica y de Salud Materna e Infantil 1989_Direct	1	Encuesta Demografica y de Salud Materna e Infantil 1989_Direct	1
	Encuesta Demografica y de Salud Materna e Infantil 1989_Indirect	1	Encuesta Demografica y de Salud Materna e Infantil 1989_Indirect	1
	Encuesta Demografica y de Salud Familiar 1987_Direct	1	Encuesta Demografica y de Salud Familiar 1987_Direct	1
	Encuesta Demografica y de Salud Familiar 1987_Indirect	1	Encuesta Demografica y de Salud Familiar 1987_Indirect	1
	ESMID 1982_Indirect	1	ESMID 1982_Indirect	1
	CENSO 1982_Indirect	1	CENSO 1982_Indirect	1
	Encuesta Nacional de Fecundidad 1979_Indirect	1	Encuesta Nacional de Fecundidad 1979_Indirect	1
	Encuesta Nacional de Fecundidad 1979_Direct	1	Encuesta Nacional de Fecundidad 1979_Direct	1
	CENSO 1974_Indirect	1	CENSO 1974_Indirect	1
	WHO Vital Registration Data 2011 version_VR (Single year)	0	Estadisticas_VR (Single year)	0
			WHO Vital Registration Data 2011 version_VR (Single year)	0
Egypt	Demographic and Health Survey 2008_Direct	1	Demographic and Health Survey 2008_Direct	0
	Demographic and Health Survey 2005_Direct	1	Demographic and Health Survey 2005_Direct	0
	Demographic and Health Survey 2005_Indirect	1	Demographic and Health Survey 2005_Indirect	0
	Demographic and Health Survey 2003_Direct	1	Demographic and Health Survey 2003_Direct	0
	Demographic and Health Survey 2003_Indirect	1	Demographic and Health Survey 2003_Indirect	0
	Demographic and Health Survey 2000_Direct	1	Demographic and Health Survey 2000_Direct	0
	Demographic and Health Survey 2000_Indirect	1	Demographic and Health Survey 2000_Indirect	0
	Demographic and Health Survey 1998_Direct	1	Demographic and Health Survey 1998_Direct	0
	Demographic and Health Survey 1997_Direct	1	Demographic and Health Survey 1997_Direct	0
	Demographic and Health Survey 1995_Direct	1	Demographic and Health Survey 1995_Direct	0
	Demographic and Health Survey 1995_Indirect	1	Demographic and Health Survey 1995_Indirect	0
	Demographic and Health Survey 1992_Direct	1	Demographic and Health Survey 1992_Direct	0
	Demographic and Health Survey 1992_Indirect	1	Demographic and Health Survey 1992_Indirect	0
	Pan Arab Project for Child Development (PAPCHILD) 1991_Indirect	1	Pan Arab Project for Child Development (PAPCHILD) 1991_Indirect	0
	Pan Arab Project for Child Development (PAPCHILD) 1991_Direct	1	Pan Arab Project for Child Development (PAPCHILD) 1991_Direct	0
	Demographic and Health Survey 1988_Direct	1	Demographic and Health Survey 1988_Direct	0
	Demographic and Health Survey 1988_Indirect	1	Demographic and Health Survey 1988_Indirect	0
	Census 1986_Indirect	0	Census 1986_Indirect	0
	Contraceptive Prevalence Survey 1984_Indirect	1	Contraceptive Prevalence Survey 1984_Indirect	0
	World Fertility Survey 1980_Indirect	1	World Fertility Survey 1980_Indirect	0
	World Fertility Survey 1980_Direct	1	World Fertility Survey 1980_Direct	0

	Census 1976_Indirect<br>WHO Vital Registration Data 2011 version_VR (Single year)	0<br>0	Census 1976_Indirect<br>Vital Registration_Single year<br>WHO Vital Registration Data 2011 version_VR (Single year)	0<br>0<br>0
El Salvador	National Family Health Survey (FESAL) 2008_Direct<br>Censo 2007_Indirect<br>FSL 2002_Direct<br>ENS 1998_Direct<br>ENS 1998_Indirect<br>Encuesta de Hogares de Propositos Multiples 1993_Indirect<br>ENS 1993_Direct<br>ENS 1993_Indirect<br>Encuesta de Hogares de Propositos Multiples 1992_Indirect<br>EHS 1992_Indirect<br>Census 1992_Indirect<br>ENS 1988_Direct<br>Encuesta Nacional de Salud Familiar (FESAL) 1985_Direct<br>Encuesta Nacional de Salud Familiar (FESAL) 1985_Indirect<br>Encuesta Nacional de Fecundidad (ENF) 1973_Indirect<br>Censo 1971_Indirect<br>WHO Vital Registration Data 2011 version_VR (Single year)	1<br>1<br>1<br>1<br>1<br>1<br>1<br>1<br>1<br>1<br>1<br>1<br>1<br>1<br>1<br>1<br>0	National Family Health Survey (FESAL) 2008_Direct<br>Censo 2007_Indirect<br>FSL 2002_Direct<br>ENS 1998_Direct<br>ENS 1998_Indirect<br>Encuesta de Hogares de Propositos Multiples 1993_Indirect<br>ENS 1993_Direct<br>ENS 1993_Indirect<br>Encuesta de Hogares de Propositos Multiples 1992_Indirect<br>EHS 1992_Indirect<br>Census 1992_Indirect<br>ENS 1988_Direct<br>Encuesta Nacional de Salud Familiar (FESAL) 1985_Direct<br>Encuesta Nacional de Salud Familiar (FESAL) 1985_Indirect<br>Demographic and Health Survey 1985_Direct<br>Encuesta Nacional de Fecundidad (ENF) 1973_Indirect<br>Censo 1971_Indirect<br>Vital Registration_Single year<br>Estadisticas Vitales Anuarios y sitio web 2009_Single year<br>WHO Vital Registration Data 2011 version_VR (Single year)	0<br>0<br>0<br>0<br>0<br>0<br>0<br>0<br>0<br>0<br>0<br>0<br>0<br>0<br>0<br>0<br>0<br>0<br>0<br>0
Equatorial Guinea	Census 2001_Single year<br>Multiple Indicator Cluster Survey 2000_Indirect<br>Census 1994_Single year<br>Census 1983_Single year	1<br>1<br>1<br>1	Census 2001_Single year<br>Multiple Indicator Cluster Survey 2000_Indirect<br>Census 1994_Single year<br>Census 1983_Single year	1<br>1<br>1<br>1
Eritrea	Demographic and Health Survey 2002_Direct<br>Demographic and Health Survey 2002_Indirect<br>Demographic and Health Survey 1995_Direct<br>Demographic and Health Survey 1995_Indirect	1<br>1<br>1<br>1	Demographic and Health Survey 2002_Direct<br>Demographic and Health Survey 2002_Indirect<br>Demographic and Health Survey 1995_Direct<br>Demographic and Health Survey 1995_Indirect	0<br>0<br>0<br>0
Estonia	WHO Good Vital Registration Data 2011 version_VR (Single year)<br>WHO Good VR 2011 adjusted version by increasing 20%_VR (Single year)	0<br>1	WHO Good Vital Registration Data 2011 version_VR (Single year)<br>WHO Good VR 2011 adjusted version by increasing 20%_VR (Single year)	0<br>1
Ethiopia	Census 2007_Indirect<br>Demographic and Health Survey 2005_Direct<br>Demographic and Health Survey 2005_Indirect<br>Demographic and Health Survey 2000_Direct<br>Demographic and Health Survey 2000_Indirect<br>Census 1994_Indirect<br>National Family and Fertility Survey 1990_Direct	1<br>1<br>1<br>1<br>1<br>0<br>1	Census 2007_Indirect<br>Demographic and Health Survey 2005_Direct<br>Demographic and Health Survey 2005_Indirect<br>Demographic and Health Survey 2000_Direct<br>Demographic and Health Survey 2000_Indirect<br>Census 1994_Indirect<br>National Family and Fertility Survey 1990_Direct	1<br>1<br>1<br>1<br>1<br>0<br>1

	National Family and Fertility Survey 1990_Indirect	1	National Family and Fertility Survey 1990_Indirect	1
	Census 1984_Indirect	0	Census 1984_Indirect	0
	Demographic Survey 1981_Indirect	1	Demographic Survey 1981_Indirect	1
Federated States of Micronesia	Census 2000_Indirect	1	Census 2000_Indirect	0
	Census 1994_Indirect	1	Census 1994_Indirect	0
Fiji	Census 1996_Indirect	1	Census 1996_Indirect	0
	Census 1986_Indirect	1	Census 1986_Indirect	0
	World Fertility Survey 1974_Direct	1	World Fertility Survey 1974_Direct	0
	WHO Vital Registration Data 2009 version_Single Year	0	UN Statistic Division Vital Registration_Single year	0
	WHO Vital Registration Data 2011 version_Single Year	1	Vital Registration_Single year	0
			WHO Vital Registration Data 2009 version_Single Year	0
			WHO Vital Registration Data 2011 version_Single Year	0
Finland	WHO Good Vital Registration Data 2011 version_VR (Single year)	1	WHO Good Vital Registration Data 2011 version_VR (Single year)	1
France	WHO Good Vital Registration Data 2011 version_VR (Single year)	1	WHO Good Vital Registration Data 2011 version_VR (Single year)	1
Gabon	Demographic and Health Survey 2001_Direct	1	Demographic and Health Survey 2001_Direct	0
	Demographic and Health Survey 2001_Indirect	1	Demographic and Health Survey 2001_Indirect	0
Gambia	Multiple Cluster Indicator Survey 2006_Indirect	1	Multiple Cluster Indicator Survey 2006_Indirect	0
	Multiple Cluster Indicator Survey 2000_Indirect	1	Multiple Cluster Indicator Survey 2000_Indirect	0
	Census 1993_Indirect	1	Census 1993_Indirect	0
	Census 1983_Indirect	1	Census 1983_Indirect	0
	Census 1973_Indirect	1	Census 1973_Indirect	0
Georgia	Reproductive Health Survey 2011_Direct	0	Reproductive Health Survey 2011_Direct	0
	Multiple Cluster Indicator Survey 2005_Indirect	1	Multiple Cluster Indicator Survey 2005_Indirect	0
	Reproductive Health Survey 2005_Direct	1	Reproductive Health Survey 2005_Direct	0
	Reproductive Health Survey 1999_Direct	1	Reproductive Health Survey 1999_Direct	0
	WHO Vital Registration Data 2011 version_Single year	0	WHO Vital Registration Data 2011 version_Single year	0
	Health facilities Data of the National Center for Disease Control and Public Health 2009_Single year	0	Health facilities Data of the National Center for Disease Control and Public Health 2009_Single year	0
Germany	WHO Good Vital Registration Data 2011 version_VR (Single year)	1	WHO Good Vital Registration Data 2011 version_VR (Single year)	1
Ghana	Demographic and Health Survey 2008_Direct	1	Demographic and Health Survey 2008_Direct	0
	Demographic and Health Survey 2008_Indirect	1	Demographic and Health Survey 2008_Indirect	0
	Maternal Health Survey 2007_Direct	1	Maternal Health Survey 2007_Direct	0
	Multiple Indicator Cluster Survey 2006_Indirect	1	Multiple Indicator Cluster Survey 2006_Indirect	0
	Demographic and Health Survey 2003_Indirect	1	Demographic and Health Survey 2003_Indirect	0
	Demographic and Health Survey 2003_Direct	1	Demographic and Health Survey 2003_Direct	0
	Demographic and Health Survey 1998_Indirect	1	Demographic and Health Survey 1998_Indirect	0
	Demographic and Health Survey 1998_Direct	1	Demographic and Health Survey 1998_Direct	0
	Demographic and Health Survey 1993_Indirect	1	Demographic and Health Survey 1993_Indirect	0
	Demographic and Health Survey 1993_Direct	1	Demographic and Health Survey 1993_Direct	0

	Demographic and Health Survey 1988_Indirect	1	Demographic and Health Survey 1988_Indirect	0
	Demographic and Health Survey 1988_Direct	1	Demographic and Health Survey 1988_Direct	0
	Ghana Fertility Survey 1979_Indirect	0	Ghana Fertility Survey 1979_Indirect	0
	Ghana Fertility Survey 1979_Direct	0	Ghana Fertility Survey 1979_Direct	0
	Census 1971_Indirect	1	Census 1971_Indirect	0
	Census 1948_Indirect	1	Census 1948_Indirect	0
	Census 1960_Indirect	1	Census 1960_Indirect	0
	CCP Registration 1974-1977_Single Year	1	CCP Registration 1974-1977_Single Year	0
	Dual Registration 1968-1969_Single Year	1	Dual Registration 1968-1969_Single Year	0
Greece	WHO Good Vital Registration Data 2011 version_VR (Single year)	0	WHO Good Vital Registration Data 2011 version_VR (Single year)	0
	WHO Good VR 2011 adjusted version by increasing 20%_VR (Single year)	1	WHO Good VR 2011 adjusted version by increasing 20%_VR (Single year)	1
Grenada	WHO Good Vital Registration Data 2011 version_VR (Single year)	1	WHO Good Vital Registration Data 2011 version_VR (Single year)	1
Guatemala	Encuesta Nacional de Salud Materno Infantil (ENSMI) 2008-2009_Direct	1	Encuesta Nacional de Salud Materno Infantil (ENSMI) 2008-2009_Direct	1
	Reproductive Health Survey 2002_Direct	1	Reproductive Health Survey 2002_Direct	1
	Censo 2002_Indirect	1	Censo 2002_Indirect	1
	Demographic and Health Survey 2002_Indirect	1	Demographic and Health Survey 2002_Indirect	1
	Demographic and Health Survey 1999_Direct	1	Demographic and Health Survey 1999_Direct	1
	Demographic and Health Survey 1999_Indirect	1	Demographic and Health Survey 1999_Indirect	1
	Demographic and Health Survey 1995_Direct	1	Demographic and Health Survey 1995_Direct	1
	Demographic and Health Survey 1995_Indirect	1	Demographic and Health Survey 1995_Indirect	1
	Enc. Nacional Sociodemografica 1989 _Indirect	1	Censo 1994_Indirect	1
	Enc. Nacional Sociodemografica 1987 _Indirect	1	Enc. Nacional Sociodemografica 1989 _Indirect	1
	Demographic and Health Survey 1987_Indirect	1	Enc. Nacional Sociodemografica 1987 _Indirect	1
	Demographic and Health Survey 1987_Direct	1	Demographic and Health Survey 1987_Indirect	1
	Censo 1981_Indirect	1	Demographic and Health Survey 1987_Direct	1
	Enc. Nacional de Fecundidad 1978_Indirect	1	Censo 1981_Indirect	1
	Censo 1973_Indirect	1	Enc. Nacional de Fecundidad 1978_Indirect	1
	WHO Vital Registration Data 2011 version_VR (Single year)	0	Censo 1973_Indirect	1
			Vital Registration 2009_Single year	0
			Est.Vitales Anuarios y Enviada por INE 2009_Single year	0
			WHO Vital Registration Data 2011 version_VR (Single year)	0
Guinea	Demographic and Health Survey 2005_Indirect	1	Demographic and Health Survey 2005_Indirect	0
	Demographic and Health Survey 2005_Direct	1	Demographic and Health Survey 2005_Direct	0
	Multiple Indicator Cluster Survey 2003_Indirect	1	Multiple Indicator Cluster Survey 2003_Indirect	0
	Demographic and Health Survey 1999_Indirect	1	Demographic and Health Survey 1999_Indirect	0
	Demographic and Health Survey 1999_Direct	1	Demographic and Health Survey 1999_Direct	0
	Demographic and Health Survey 1992_Indirect	1	Demographic and Health Survey 1992_Indirect	0
	Demographic and Health Survey 1992_Direct	1	Demographic and Health Survey 1992_Direct	0
	Survey 1954-55_Indirect	1	Survey 1954-55_Indirect	0
	Survey HH deaths 1954-55_Single Year	1	Survey HH deaths 1954-55_Single Year	0

	Census 1996_Indirect	0	Census 1996_Indirect	0
Guinea-Bissau	Multiple Indicator Cluster Survey 2010_Indirect	1	Multiple Indicator Cluster Survey 2010_Indirect	1
	Multiple Indicator Cluster Survey 2010_Direct	1	Multiple Indicator Cluster Survey 2010_Direct	1
	Multiple Indicator Cluster Survey 2006_Indirect	1	Multiple Indicator Cluster Survey 2006_Indirect	1
	Multiple Indicator Cluster Survey 2000_Indirect	1	Multiple Indicator Cluster Survey 2000_Indirect	1
	Census 1950_Indirect	0	Census 1950_Indirect	0
Guyana	Demographic and Health Survey Preliminary 2009_Direct	1	Demographic and Health Survey Preliminary 2009_Direct	0
	Multiple Indicator Cluster Survey 2006_Indirect	1	Multiple Indicator Cluster Survey 2006_Indirect	0
	AIDS Indicator Survey 2005_Direct	1	AIDS Indicator Survey 2005_Direct	0
	AIDS Indicator Survey 2005_Indirect	1	AIDS Indicator Survey 2005_Indirect	0
	Census 2002_Indirect	1	Census 2002_Indirect	0
	Multiple Indicator Cluster Survey 2000_Indirect	1	Multiple Indicator Cluster Survey 2000_Indirect	0
	World Fertility Survey 1975_Indirect	1	World Fertility Survey 1975_Indirect	0
	WHO Vital Registration Data 2009 version_VR (Single year)	0	WHO Vital Registration Data 2009 version_VR (Single year)	0
	WHO Vital Registration Data 2011 version_VR (Single year)	0	WHO Vital Registration Data 2011 version_VR (Single year)	0
	WHO Vital Registration Data 2011 version Moving Average_VR (Single year)	0	WHO Vital Registration Data 2011 version Moving Average_VR (Single year)	0
Haiti	Demographic and Health Survey 2005_Direct	1	Demographic and Health Survey 2005_Direct	0
	Demographic and Health Survey 2005_Indirect	1	Demographic and Health Survey 2005_Indirect	0
	Censo 2003_Indirect	0	Censo 2003_Indirect	0
	Demographic and Health Survey 2000_Direct	1	Demographic and Health Survey 2000_Direct	0
	Demographic and Health Survey 2000_Indirect	1	Demographic and Health Survey 2000_Indirect	0
	Demographic and Health Survey 1994_Direct	1	Demographic and Health Survey 1994_Direct	0
	Demographic and Health Survey 1994_Indirect	1	Demographic and Health Survey 1994_Indirect	0
	Enquête Mortalité, Morbidité et Utilisation des Services (EMMUS)	1	Enquête Mortalité, Morbidité et Utilisation des Services (EMMUS) 1987_Direct	0
	1987_Direct	1	Enquête Mortalité, Morbidité et Utilisation des Services (EMMUS)	0
	Enquête Mortalité, Morbidité et Utilisation des Services (EMMUS)	1	1987_Indirect	0
	1987_Indirect	1	Contraceptive Prevalence Survey 1983_Indirect	0
	Contraceptive Prevalence Survey 1983_Indirect	1	Census 1982_Indirect	0
	Census 1982_Indirect	1	World Fertility Survey 1977_Indirect	0
	World Fertility Survey 1977_Indirect	0	World Fertility Survey 1977_Direct	0
	World Fertility Survey 1977_Direct	0	Census 1971_Direct	0
	Census 1971_Direct	0	Demographic Survey Repeated Passages 1971_Direct	0
	WHO Vital Registration Data 2009 version_VR (Single year)		WHO Vital Registration Data 2009 version_VR (Single year)	0
	WHO Vital Registration Data 2011 version_VR (Single year)		WHO Vital Registration Data 2011 version_VR (Single year)	
Honduras	Demographic and Health Survey 2005_Direct	1	Demographic and Health Survey 2005_Direct	0
	Demographic and Health Survey 2005_Indirect	1	Demographic and Health Survey 2005_Indirect	0
	National Survey of Family Health and Epidemiology 2001_Direct	1	National Survey of Family Health and Epidemiology 2001_Direct	0
	Censo 2001_Indirect	1	Censo 2001_Indirect	0
	National Survey of Family Health and Epidemiology 1996_Indirect	1	National Survey of Family Health and Epidemiology 1996_Indirect	0
	National Survey of Family Health and Epidemiology 1996_Direct	1	National Survey of Family Health and Epidemiology 1996_Direct	0

	National Survey of Family Health and Epidemiology 1991_Indirect	1	National Survey of Family Health and Epidemiology 1991_Indirect	0
	National Survey of Family Health and Epidemiology 1991_Direct	1	National Survey of Family Health and Epidemiology 1991_Direct	0
	Census 1988_Indirect	1	Census 1988_Indirect	0
	National Survey of Family Health and Epidemiology 1987_Indirect	1	National Survey of Family Health and Epidemiology 1987_Indirect	0
	National Survey of Family Health and Epidemiology 1987_Direct	1	National Survey of Family Health and Epidemiology 1987_Direct	0
	National Survey of Maternal and Child Health 1984_Indirect	1	National Survey of Maternal and Child Health 1984_Indirect	0
	National Demographic Survey 1983_Indirect	1	National Demographic Survey 1983_Indirect	0
	Census 1974_Indirect	1	Census 1974_Indirect	0
	National Demographic Survey 1972_Indirect	1	National Demographic Survey 1972_Indirect	0
	National Demographic Survey 1972_Direct	1	National Demographic Survey 1972_Direct	0
	WHO Vital Registration Data 2009 version_VR (Single year)	0	Est.Vitales_ Anuarios_Enero 2009_Single year	0
	WHO Vital Registration Data 2011 version_VR (Single year)	0	WHO Vital Registration Data 2009 version_VR (Single year)	0
			WHO Vital Registration Data 2011 version_VR (Single year)	0
Hungary	WHO Good Vital Registration Data 2011 version_VR (Single year)	0	WHO Good Vital Registration Data 2011 version_VR (Single year)	0
	WHO Good VR 2011 adjusted version by increasing 20%_VR (Single year)	1	WHO Good VR 2011 adjusted version by increasing 20%_VR (Single year)	1
Iceland	WHO Good Vital Registration Data 2011 version_VR (Single year)	1	WHO Good Vital Registration Data 2011 version_VR (Single year)	1
India	National Family Health Survey 2005-06_Indirect	1	National Family Health Survey 2005-06_Indirect	0
	National Family Health Survey 2005-06_Direct	0	National Family Health Survey 2005-06_Direct	0
	Demographic and Health Survey 2005_Direct	1	Demographic and Health Survey 2005_Direct	0
	Census 2001_Indirect	0	Census 2001_Indirect	0
	National Family Health Survey 1999_Direct	1	National Family Health Survey 1999_Direct	0
	National Family Health Survey 1999_Indirect	1	National Family Health Survey 1999_Indirect	0
	National Family Health Survey 1992_Direct	1	National Family Health Survey 1992_Direct	0
	National Family Health Survey 1992_Indirect	1	National Family Health Survey 1992_Indirect	0
	Census 1981_Indirect	0	Census 1981_Indirect	0
	National Family Planning Survey 1980_Indirect	1	National Family Planning Survey 1980_Indirect	0
	Survey on Infant and Child Mortality 1979_Indirect	0	Survey on Infant and Child Mortality 1979_Indirect	0
	National Family Planning Survey 1970_Indirect	1	National Family Planning Survey 1970_Indirect	0
	Sample Registration System_VR (Single year)	1	Sample Registration System_VR (Single year)	0
Indonesia	Demographic and Health Survey 2007_Direct	1	Demographic and Health Survey 2007_Direct	0
	Demographic and Health Survey 2007_Indirect	1	Demographic and Health Survey 2007_Indirect	0
	Demographic and Health Survey 2002_Direct	1	Demographic and Health Survey 2002_Direct	0
	Demographic and Health Survey 2002_Indirect	1	Demographic and Health Survey 2002_Indirect	0
	Census 2000_Indirect	1	Census 2000_Indirect	0
	Demographic and Health Survey 1997_Direct	1	Demographic and Health Survey 1997_Direct	0
	Demographic and Health Survey 1997_Indirect	1	Demographic and Health Survey 1997_Indirect	0
	Demographic and Health Survey 1994_Direct	1	Demographic and Health Survey 1994_Direct	0
	Demographic and Health Survey 1994_Indirect	1	Demographic and Health Survey 1994_Indirect	0
	Demographic and Health Survey 1991_Direct	1	Demographic and Health Survey 1991_Direct	0
	Demographic and Health Survey 1991_Indirect	1	Demographic and Health Survey 1991_Indirect	0

	Census 1990_Indirect	1	Census 1990_Indirect	0
	Demographic and Health Survey 1987_Direct	1	Demographic and Health Survey 1987_Direct	0
	Demographic and Health Survey 1987_Indirect	1	Demographic and Health Survey 1987_Indirect	0
	Census 1980_Indirect	1	Census 1980_Indirect	0
	World Fertility Survey 1976_Direct	1	World Fertility Survey 1976_Direct	0
	World Fertility Survey 1976_Indirect	1	World Fertility Survey 1976_Indirect	0
	Census 1971_Indirect	1	Census 1971_Indirect	0
Iran	Demographic and Health Survey 2000_Indirect	1	Demographic and Health Survey 2000_Indirect	0
	Demographic and Health Survey 2000_Direct	1	Demographic and Health Survey 2000_Direct	0
	Demographic Survey 1998_Indirect	1	Demographic Survey 1998_Indirect	0
	Iran National Multiple Indicator Cluster Survey 1998_Single year	1	Iran National Multiple Indicator Cluster Survey 1998_Single year	0
	Census 1996_Indirect	1	Census 1996_Indirect	0
	Iran National Multiple Indicator Cluster Survey 1995_Single year	1	Iran National Multiple Indicator Cluster Survey 1995_Single year	0
	Intercensal Population Survey 1991_Indirect	1	Intercensal Population Survey 1991_Indirect	0
	Infant and Child Mortality Survey 1989_Direct	1	Infant and Child Mortality Survey 1989_Direct	0
	Infant and Child Mortality Survey 1989_Indirect	1	Infant and Child Mortality Survey 1989_Indirect	0
	Census 1986_Indirect	1	Census 1986_Indirect	0
	Iran National Multiple Indicator Cluster Survey 1985_Single year	1	Iran National Multiple Indicator Cluster Survey 1985_Single year	0
	Population Growth Survey 1973_Indirect	1	Population Growth Survey 1973_Indirect	0
	Population Growth Survey 1973_Direct	1	Population Growth Survey 1973_Direct	0
	Iran National Child Mortality Surveillance System_Single year	1	Iran National Child Mortality Surveillance System_Single year	0
	Demographic and Health Survey 2000_Single year	1	Demographic and Health Survey 2000_Single year	0
	Pop-Health-Survey 1974_Single year	1	Pop-Health-Survey 1974_Single year	0
	Census 2006_Indirect	1	Fertility Survey 1977_Indirect	0
			SCIH Survey 1987 _Indirect	0
			MPC Survey 1990_Indirect	0
			Births Survey 1991_Indirect	0
			IPC Survey 1992_Indirect	0
			SCIH Survey 1992_Indirect	0
			SCIH Survey 1993_Indirect	0
			IPC Survey 1994_Indirect	0
			SCIH Survey 1994_Indirect	0
			SCIH Survey 1995_Indirect	0
			SCIH Survey 2001_Indirect	0
			Census 2006_Indirect	0
			IPC Survey 1993_Indirect	0
Iraq	Iraq Family Health Survey 2006_Indirect	1	Iraq Family Health Survey 2006_Indirect	0
	Multiple Indicator Cluster Survey 2006_Indirect	1	Multiple Indicator Cluster Survey 2006_Indirect	0
	Census 1997_Indirect	0	Census 1997_Indirect	0
	Immunization, Diarrhoeal Disease, Maternal and Child Mortality Survey	1	Immunization, Diarrhoeal Disease, Maternal and Child Mortality Survey	0

	1990_Indirect	0	1990_Indirect	0
	Gulf Child Health Survey 1989_Direct	1	Gulf Child Health Survey 1989_Direct	0
	Gulf Child Health Survey 1989_Indirect	1	Gulf Child Health Survey 1989_Indirect	0
	Census 1987_Indirect	1	Census 1987_Indirect	0
	Fertility Survey 1974_Indirect	1	Fertility Survey 1974_Indirect	0
	Demographic Sample Survey and Sample Registration System 1973_Direct	1	Demographic Sample Survey and Sample Registration System 1973_Direct	0
	Multiple Indicator Cluster Survey _Single year	0	Multiple Indicator Cluster Survey _Single year	0
	Infant and Child Mortality and Nutrition Survey_Single year	1	Infant and Child Mortality and Nutrition Survey_Single year	0
	Living Conditions Survey_Single year	0	Living Conditions Survey_Single year	0
	Child and Maternal Mortality Survey_Single year	1	Child and Maternal Mortality Survey_Single year	0
	Census 1957_Indirect		Census 1957_Indirect	
Ireland	WHO Good Vital Registration Data 2011 version_VR (Single year)	1	WHO Good Vital Registration Data 2011 version_VR (Single year)	1
Israel	National Life table 1969-1988_Single year	0	National Life table 1969-1988_Single year	
	WHO Good Vital Registration Data 2011 version_VR (Single year)	1	Vital registration from Vital Statistica and Statistical Abstract 2008_Single year	
			WHO Good Vital Registration Data 2011 version_VR (Single year)	
Italy	WHO Good Vital Registration Data 2011 version_VR (Single year)	1	WHO Good Vital Registration Data 2011 version_VR (Single year)	1
Jamaica	Multiple Indicator Cluster Survey 2006_Indirect	1	Multiple Indicator Cluster Survey 2006_Indirect	0
	Multiple Indicator Cluster Survey 2000_Indirect	0	Multiple Indicator Cluster Survey 2000_Indirect	0
	Contraceptive Prevalence Survey 1989_Direct	0	Contraceptive Prevalence Survey 1989_Direct	0
	Census 1982_Indirect	1	Census 1982_Indirect	0
	Jamaica Fertility Survey 1975_Direct	1	Jamaica Fertility Survey 1975_Direct	0
	Jamaica Fertility Survey 1975_Indirect	1	Jamaica Fertility Survey 1975_Indirect	0
	National Life Tables_Single year	1	National Life Tables_Single year	0
			Vital Registration_Single year	0
Japan	WHO Good Vital Registration Data 2011 version_VR (Single year)	1	WHO Good Vital Registration Data 2011 version_VR (Single year)	1
Jordan	Demographic and Health Survey 2009_Direct	1	Demographic and Health Survey 2009_Direct	1
	Demographic and Health Survey 2009_Indirect	1	Demographic and Health Survey 2009_Indirect	1
	Demographic and Health Survey 2007_Indirect	1	Demographic and Health Survey 2007_Indirect	1
	Demographic and Health Survey 2007_Direct	1	Demographic and Health Survey 2007_Direct	1
	Demographic and Health Survey 2002_Indirect	1	Demographic and Health Survey 2002_Indirect	1
	Demographic and Health Survey 2002_Direct	1	Demographic and Health Survey 2002_Direct	1
	Annual Fertility Survey 1999_Indirect	1	Annual Fertility Survey 1999_Indirect	1
	Demographic and Health Survey 1997_Indirect	1	Demographic and Health Survey 1997_Indirect	1
	Demographic and Health Survey 1997_Direct	1	Demographic and Health Survey 1997_Direct	1
	EPI-CDD and Child Mortality Survey 1990_Indirect	1	EPI-CDD and Child Mortality Survey 1990_Indirect	1
	Demographic and Health Survey 1990_Indirect	1	Demographic and Health Survey 1990_Indirect	1
	Demographic and Health Survey 1990_Direct	1	Demographic and Health Survey 1990_Direct	1
	EPI-CDD and Child Mortality Survey 1988_Indirect	1	EPI-CDD and Child Mortality Survey 1988_Indirect	1
	Jordan Demographic Survey 1981_Indirect	1	Jordan Demographic Survey 1981_Indirect	1
	Census 1979_Indirect	1	Census 1979_Indirect	1

	World Fertility Survey 1976_Direct	1	World Fertility Survey 1976_Direct	1
	World Fertility Survey 1976_Indirect	1	World Fertility Survey 1976_Indirect	1
	Jordan Fertility Survey 1972_Indirect	1	Jordan Fertility Survey 1972_Indirect	1
	WHO Vital Registration Data 2009 version_Single Year	0	WHO Vital Registration Data 2009 version_Single Year	0
	Census 1961_Indirect	1	Census 1961_Indirect	1
	WHO Vital Registration Data 2011 version_VR (Single year)	0	WHO Vital Registration Data 2011 version_VR (Single year)	0
Kazakhstan	Multiple Indicator Cluster Survey 2006_Indirect	1	Multiple Indicator Cluster Survey 2006_Indirect	0
	Census 1999_Indirect	0	Census 1999_Indirect	0
	Demographic and Health Survey 1999_Direct	1	Demographic and Health Survey 1999_Direct	0
	Demographic and Health Survey 1999_Indirect	1	Demographic and Health Survey 1999_Indirect	0
	Lsms 1996_Indirect	1	Lsms 1996_Indirect	0
	Demographic and Health Survey 1995_Direct	1	Demographic and Health Survey 1995_Direct	0
	Demographic and Health Survey 1995_Indirect	1	Demographic and Health Survey 1995_Indirect	0
	Census 1989_Indirect	1	Census 1989_Indirect	0
	WHO Vital Registration_VR (Single year)	0	Transmonee Vital Registration Data 2008 version_VR (Single year)	0
	Transmonee Vital Registration Data 2008 version_VR (Single year)	0	Vital Registration Data from The Agency of Statistics of Kazakhstan_VR (Single year)	0
	Vital Registration Data from The Agency of Statistics of Kazakhstan_VR (Single year)	0	WHO Vital Registration Data 2011 version_VR (Single year)	0
	WHO Vital Registration Data 2011 version_VR (Single year)	1		
Kenya	Demographic and Health Survey 2008_Direct	1	Demographic and Health Survey 2008_Direct	0
	Demographic and Health Survey 2008_Indirect	1	Demographic and Health Survey 2008_Indirect	0
	Demographic and Health Survey 2003_Direct	1	Demographic and Health Survey 2003_Direct	0
	Demographic and Health Survey 2003_Indirect	1	Demographic and Health Survey 2003_Indirect	0
	Multiple Indicator Cluster Survey 2000_Indirect	1	Multiple Indicator Cluster Survey 2000_Indirect	0
	Demographic and Health Survey 1998_Direct	1	Demographic and Health Survey 1998_Direct	0
	Demographic and Health Survey 1998_Indirect	1	Demographic and Health Survey 1998_Indirect	0
	Demographic and Health Survey 1993_Direct	1	Demographic and Health Survey 1993_Direct	0
	Demographic and Health Survey 1992_Direct	1	Demographic and Health Survey 1992_Direct	0
	Demographic and Health Survey 1992_Indirect	0	Demographic and Health Survey 1992_Indirect	0
	Census 1989_Indirect	1	Census 1989_Indirect	0
	Demographic and Health Survey 1988_Direct	1	Demographic and Health Survey 1988_Direct	0
	Demographic and Health Survey 1988_Indirect	1	Demographic and Health Survey 1988_Indirect	0
	Demographic Survey 1983_Indirect	1	Demographic Survey 1983_Indirect	0
	Census 1979_Indirect	1	Census 1979_Indirect	0
	Demographic Survey 1977_Indirect	1	Demographic Survey 1977_Indirect	0
	World Fertility Survey 1977_Direct	1	World Fertility Survey 1977_Direct	0
	World Fertility Survey 1977_Indirect	1	World Fertility Survey 1977_Indirect	0
	Census 1969_Indirect	1	Census 1969_Indirect	0
Kiribati	Census 2005_Indirect	1	Census 2005_Indirect	0
	Census 1995_Indirect	1	Census 1995_Indirect	0

	Census 1990_Indirect	1	Census 1990_Indirect	0
	Census 1978_Indirect	1	Census 1978_Indirect	0
	WHO Vital Registration Data 2009 version_VR (Single year)	0	WHO Vital Registration Data 2009 version_VR (Single year)	0
	WHO Vital Registration Data 2011 version_VR (Single year)	0	WHO Vital Registration Data 2011 version_VR (Single year)	0
	WHO Vital Registration Data 2011 version Moving Average_VR (Single year)	0	WHO Vital Registration Data 2011 version Moving Average_VR (Single year)	0
Korea DPR	Census 2008_Single year	1	Census 2008_Single year	1
	Civil Registration data from Central Bureau of Statistics 2000_Single year	1	Civil Registration data from Central Bureau of Statistics 2000_Single year	1
	Census 1993_Single yea	1	Census 1993_Single yea	1
Korea Rep	Census 1990_Indirect	1	Census 1990_Indirect	0
	Census 1985_Indirect	1	Korea Fertility Survey 1988_Direct	0
	Census 1980_Indirect	1	Census 1985_Indirect	0
	Census 1975_Indirect	1	Census 1980_Indirect	0
	Korea Fertility Survey 1974_Indirect	1	Census 1975_Indirect	0
	Korea Fertility Survey 1974_Direct	1	Korea Fertility Survey 1974_Indirect	0
	Census 1970_Indirect	1	Korea Fertility Survey 1974_Direct	0
	National Life Table_Single year	1	Fertility-Abortion Survey 1971_Single year	0
	WHO Vital Registration Data 2011 version_Single year	1	Census 1970_Indirect	0
			National Life Table_Single year	0
			WHO Vital Registration Data 2011 version_Single year	0
Kuwait	Census 1980_Indirect	1	Census 1980_Indirect	1
	Census 1975_Indirect	1	Census 1975_Indirect	1
	National Life Tables_Single year	1	Vital Registration from Annual Statistical Abstract_Single year	0
	WHO Good Vital Registration Data 2011 version_Single year	1	National Life Tables_Single year	1
			WHO Good Vital Registration Data 2011 version_Single year	1
Kyrgyzstan	Multiple Indicator Cluster Survey 2006_Indirect	1	Multiple Indicator Cluster Survey 2006_Indirect	1
	Demographic and Health Survey 1997_Direct	1	Demographic and Health Survey 1997_Direct	1
	Demographic and Health Survey 1997_Indirect	1	Demographic and Health Survey 1997_Indirect	1
	WHO Vital Registration Data 2011 version_VR (Single year)	1	WHO Vital Registration Data 2011 version_VR (Single year)	1
Lao PDR	Reproductive Health Survey 2005_Direct	0	Reproductive Health Survey 2005_Direct	1
	Reproductive Health Survey 2005_Indirect	0	Reproductive Health Survey 2005_Indirect	1
	Census 2005_Indirect	0	Census 2005_Indirect	1
	Reproductive Health Survey 2000_Direct	0	Reproductive Health Survey 2000_Direct	1
	Census 1995_Indirect	0	Census 1995_Indirect	1
	Fertility and Birth Spacing Survey 1994_Direct	0	Fertility and Birth Spacing Survey 1994_Direct	1
	Social Indicator Survey 1993_Indirect	0	Social Indicator Survey 1993_Indirect	1
			Laos Health Survey 1968_Direct	1
Latvia	WHO Good Vital Registration Data 2011 version_VR (Single year)	0	WHO Good Vital Registration Data 2011 version_VR (Single year)	0
	WHO Good VR 2011 adjusted version by increasing 20%_VR (Single year)	1	WHO Good VR 2011 adjusted version by increasing 20%_VR (Single year)	1
Lebanon	PAPFAM Family Health Survey 2004_Direct	1	PAPFAM Family Health Survey 2004_Direct	0
	Multiple Cluster Indicator Survey 2000_Indirect	1	Multiple Cluster Indicator Survey 2000_Indirect	0

	PAPCHILD Maternal and Child Health Survey 1996_Direct	1	PAPCHILD Maternal and Child Health Survey 1996_Direct	0
	National EPI CDD IMR Survey 1990_Indirect	1	National EPI CDD IMR Survey 1990_Indirect	0
	National Fertility and Family Planning Survey 1971_Indirect	1	National Fertility and Family Planning Survey 1971_Indirect	0
Lesotho	Demographic and Health Survey 2009_Direct	0	Demographic and Health Survey 2009_Direct	0
	Demographic and Health Survey 2009_Indirect	0	Demographic and Health Survey 2009_Indirect	0
	Census 2006_Single yea	0	Census 2006_Single yea	0
	Census 2006_Indirect	0	Census 2006_Indirect	0
	Demographic and Health Survey 2004_Direct	0	Demographic and Health Survey 2004_Direct	0
	Demographic and Health Survey 2004_Indirect	0	Demographic and Health Survey 2004_Indirect	0
	Demographic Survey 2001_Indirect	0	Demographic Survey 2001_Indirect	0
	Multiple Indicator Cluster Survey 2000_Indirect	0	Multiple Indicator Cluster Survey 2000_Indirect	0
	Census 1996_Indirect	0	Census 1996_Indirect	0
	Census 1986_Indirect	0	Census 1986_Indirect	0
	World Fertility Survey 1977_Indirect	0	World Fertility Survey 1977_Indirect	0
	World Fertility Survey 1977_Direct	0	World Fertility Survey 1977_Direct	0
	Census 1976_Indirect	0	Census 1976_Indirect	0
	Lds 1971_Indirect	0	Lds 1971_Indirect	0
	Ces 1968_Indirect	0	Ces 1968_Indirect	0
Liberia	Malaria Indicator Survey 2009_Indirect	1	Malaria Indicator Survey 2009_Indirect	0
	Malaria Indicator Survey 2009_Direct	1	Malaria Indicator Survey 2009_Direct	0
	Population and Housing Census 2008_Indirect	0	Population and Housing Census 2008_Indirect	0
	Demographic and Health Survey 2007_Indirect	1	Demographic and Health Survey 2007_Indirect	0
	Demographic and Health Survey 2007_Direct	1	Demographic and Health Survey 2007_Direct	0
	LDHS 1999-2000_Direct	0	LDHS 1999-2000_Direct	0
	Demographic and Health Survey 1986_Indirect	1	Demographic and Health Survey 1986_Indirect	0
	Demographic and Health Survey 1986_Direct	1	Demographic and Health Survey 1986_Direct	0
	Census 1974_Indirect	0	National Demographic Survey 1978_Single year	0
	Population and Growth Survey 1970-1971_Indirect	1	Census 1974_Indirect	0
	Population and Growth Survey 1970-1971_Direct	1	Population and Growth Survey 1970-1971_Indirect	0
	Population and Growth Survey 1969-1970_Indirect	0	Population and Growth Survey 1970-1971_Direct	0
	Population and Growth Survey 1969-1970_Direct	0	Population and Growth Survey 1969-1970_Indirect	0
			Population and Growth Survey 1969-1970_Direct	0
Libya	Family Health Survey (PAPFAM) 2007_Direct	1	Family Health Survey (PAPFAM) 2007_Direct	0
	Pan Arab Project for Child Development (PAPCHILD) 1995_Direct	1	Pan Arab Project for Child Development (PAPCHILD) 1995_Direct	0
	Census 1973_Indirect	1	Census 1973_Indirect	0
	VR Data from Health and Environment Report 2009_Single year	1	Vital Registration_Single year	0
			VR Data from Health and Environment Report 2009_Single year	0
Liechtenstein	The State of the World's Children (SOWC) 2009 Estimates_Single year	1	The State of the World's Children (SOWC) 2009 Estimates_Single year	1
Lithuania	WHO Good Vital Registration Data 2011 version_VR (Single year)	0	WHO Good Vital Registration Data 2011 version_VR (Single year)	0
	WHO Good VR 2011 adjusted version by increasing 20%_VR (Single year)	1	WHO Good VR 2011 adjusted version by increasing 20%_VR (Single year)	1

Luxembourg	WHO Good Vital Registration Data 2011 version_VR (Single year)	1	WHO Good Vital Registration Data 2011 version_VR (Single year)	1
Macedonia	Multiple Indicator Cluster Survey 2005_Indirect	0	Multiple Indicator Cluster Survey 2005_Indirect	0
	WHO Vital Registration Data 2011 version_VR (Single year)	0	WHO Vital Registration Data 2011 version_VR (Single year)	1
Madagascar	Demographic and Health Survey 2008_Direct	1	Demographic and Health Survey 2008_Direct	0
	Demographic and Health Survey 2008_Indirect	1	Demographic and Health Survey 2008_Indirect	0
	Demographic and Health Survey 2004_Direct	1	Demographic and Health Survey 2004_Direct	0
	Demographic and Health Survey 2004_Indirect	1	Demographic and Health Survey 2004_Indirect	0
	Multiple Indicator Cluster Survey 2000_Indirect	1	Multiple Indicator Cluster Survey 2000_Indirect	0
	Demographic and Health Survey 1997_Direct	1	Demographic and Health Survey 1997_Direct	0
	Demographic and Health Survey 1997_Indirect	1	Demographic and Health Survey 1997_Indirect	0
	Multiple Indicator Cluster Survey 1995_Indirect	1	Multiple Indicator Cluster Survey 1995_Indirect	0
	Demographic and Health Survey 1992_Direct	1	Demographic and Health Survey 1992_Direct	0
	Demographic and Health Survey 1992_Indirect	1	Demographic and Health Survey 1992_Indirect	0
	Madagascar Demographic Survey 1966_Indirect	0	Madagascar Demographic Survey 1966_Indirect	0
Malawi	Demographic and Health Survey (Preliminary) 2010_Direct	1	Demographic and Health Survey (Preliminary) 2010_Direct	0
	Multiple Indicator Cluster Survey 2006_Indirect	1	Multiple Indicator Cluster Survey 2006_Indirect	0
	Multiple Indicator Cluster Survey 2006_Direct	1	Multiple Indicator Cluster Survey 2006_Direct	0
	Demographic and Health Survey 2004_Direct	1	Demographic and Health Survey 2004_Direct	0
	Demographic and Health Survey 2004_Indirect	1	Demographic and Health Survey 2004_Indirect	0
	Demographic and Health Survey 2000_Direct	1	Demographic and Health Survey 2000_Direct	0
	Demographic and Health Survey 2000_Indirect	1	Demographic and Health Survey 2000_Indirect	0
	Demographic and Health Survey 1992_Direct	1	Demographic and Health Survey 1992_Direct	0
	Demographic and Health Survey 1992_Indirect	1	Demographic and Health Survey 1992_Indirect	0
	Census 1987_Indirect	1	Census 1987_Indirect	0
	Ffs 1984_Direct	1	Ffs 1984_Direct	0
	Ffs 1984_Indirect	1	Ffs 1984_Indirect	0
	Mds 1982_Indirect	1	Mds 1982_Indirect	0
	Census 1977_Indirect	1	Census 1977_Indirect	0
	Pcs 1970_Direct	1	Pcs 1970_Direct	0
	Pcs 1970_Indirect	1	Pcs 1970_Indirect	0
Malaysia	Fertility and Family Survey 1974_Direct	1	Fertility and Family Survey 1974_Direct	0
	Census 1970_Indirect	1	Census 1970_Indirect	0
	Vital Registration Data from Dept. of Statistics_Single year	0	Vital Registration Data from Dept. of Statistics_Single year	0
	National Life Table_Single year	1	National Life Table_Single year	0
	WHO Vital Registration Data 2011 version_VR (Single year)	0	Vital Registration UN Statistic Division_Single year	0
	Vital Registration Data from Department of Statistics 2010_VR (Single year)	1	WHO Vital Registration Data 2011 version_VR (Single year)	0
			Vital Registration Data from Department of Statistics 2010_VR (Single year)	0
Maldives	Demographic and Health Survey 2009_Direct	1	Demographic and Health Survey 2009_Direct	1
	Demographic and Health Survey 2009_Indirect	1	Demographic and Health Survey 2009_Indirect	1
	Census 2006_Indirect	1	Census 2006_Indirect	1

	Census 2000_Indirect	1	Census 2000_Indirect	1
	Poverty and Vulnerability Survey 1997_Indirect	1	Poverty and Vulnerability Survey 1997_Indirect	1
	Census 1995_Indirect	1	Census 1995_Indirect	1
	Census 1990_Indirect	1	Census 1990_Indirect	1
	Census 1985_Indirect	1	Census 1985_Indirect	1
	Census 1977_Indirect	1	Census 1977_Indirect	1
	Census 2000_Indirect	0	Census 2000_Indirect	0
	WHO Vital Registration Data 2011 version_VR (Single year)	0	WHO Vital Registration Data 2011 version_VR (Single year)	0
Mali	Demographic and Health Survey 2006_Direct	1	Demographic and Health Survey 2006_Direct	0
	Demographic and Health Survey 2006_Indirect	1	Demographic and Health Survey 2006_Indirect	0
	Demographic and Health Survey 2001_Direct	1	Demographic and Health Survey 2001_Direct	0
	Demographic and Health Survey 2001_Indirect	1	Demographic and Health Survey 2001_Indirect	0
	Demographic and Health Survey 1995_Direct	1	Demographic and Health Survey 1995_Direct	0
	Demographic and Health Survey 1995_Indirect	1	Demographic and Health Survey 1995_Indirect	0
	Demographic and Health Survey 1987_Direct	1	Demographic and Health Survey 1987_Direct	0
	Demographic and Health Survey 1987_Indirect	1	Demographic and Health Survey 1987_Indirect	0
	Census 1976_Indirect	0	Census 1976_Indirect	0
	Census 1998_Indirect	0	Census 1998_Indirect	0
	Census 1987_Indirect	0	Census 1987_Indirect	0
	Survey 1960-61_Indirect	0	Survey 1960-61_Indirect	0
	Survey 1956-58_Indirect	0	Survey 1956-58_Indirect	0
Malta	WHO Good Vital Registration Data 2011 version_VR (Single year)	1	WHO Good Vital Registration Data 2011 version_VR (Single year)	1
Marshall Islands	Demographic and Health Survey 2007_Direct	1	Demographic and Health Survey 2007_Direct	0
	Demographic and Health Survey 2007_Indirect	1	Demographic and Health Survey 2007_Indirect	0
	Census 1999_Indirect	1	Census 1999_Indirect	0
	Census 1988_Indirect	1	Census 1988_Indirect	0
	Census 1973_Indirect	1	Census 1973_Indirect	0
	WHO Vital Registration Data 2011 version_VR (Single year)	0	WHO Vital Registration Data 2011 version_VR (Single year)	0
Mauritania	Multiple Indicator Cluster Survey 2007_Indirect	1	Multiple Indicator Cluster Survey 2007_Indirect	0
	Demographic and Health Survey 2000_Direct	1	Demographic and Health Survey 2000_Direct	0
	Demographic and Health Survey 2000_Indirect	1	Demographic and Health Survey 2000_Indirect	0
	EMIP 2000_Direct	1	EMIP 2000_Direct	0
	EMIP 2000_Indirect	1	EMIP 2000_Indirect	0
	Multiple Indicator Cluster Survey 1995_Indirect	0	Multiple Indicator Cluster Survey 1995_Indirect	0
	MCHS 1990_Direct	1	MCHS 1990_Direct	0
	MCHS 1990_Indirect	1	MCHS 1990_Indirect	0
	World Fertility Survey 1981_Direct	1	World Fertility Survey 1981_Direct	0
	World Fertility Survey 1981_Indirect	1	World Fertility Survey 1981_Indirect	0
	EMIP survey 2003-04_Direct	1	EMIP survey 2003-04_Direct	0
	Fouta-Toro-Survey 1957_Indirect	1	Fouta-Toro-Survey 1957_Indirect	0

	Survey 1964-65_Indirect	0	Survey 1964-65_Indirect	0
	Census 1988_Indirect	1	Census 1988_Indirect	0
	Census 1977_Indirect	1	Census 1977_Indirect	0
	EMIP survey 2003-04_Indirect	1	EMIP survey 2003-04_Indirect	0
Mauritius	WHO Good Vital Registration Data 2011 version_VR (Single year)	1	WHO Good Vital Registration Data 2011 version_VR (Single year)	1
Mexico	Census 2010_Indirect	1	Census 2010_Indirect	1
	ENADID 2007_Single year	0	ENADID 2007_Single year	0
	ENADID 2006_Indirect	1	ENADID 2006_Indirect	1
	Conteo 2005_Indirect	1	ESTADISTICAS_VITALES_NAC_RECONST 2006_Single year	0
	Census 2000_ Indirect	1	Conteo 2005_Indirect	1
	ndd 1992_Indirect	1	Census 2000_ Indirect	1
	ndd 1992_Direct	1	Encuesta Nacional de la Dinamica Demografica ENADI 1997_Direct	1
	Census 1990_ Indirect	1	ndd 1992_Indirect	1
	Demographic and Health Survey 1987_Direct	1	ndd 1992_Direct	1
	Demographic and Health Survey 1987_Indirect	1	Census 1990_ Indirect	1
	Census 1980_ Indirect	1	Demographic and Health Survey 1987_Direct	1
	Contraceptive Prevalence Survey 1979_Indirect	1	Demographic and Health Survey 1987_Indirect	1
	World Fertility Survey 1976 _Indirect	1	Census 1980_ Indirect	1
	World Fertility Survey 1976 _Direct	1	Contraceptive Prevalence Survey 1979_Indirect	1
	WHO Vital Registration Data 2011 version_Single year	1	World Fertility Survey 1976 _Indirect	1
			World Fertility Survey 1976 _Direct	1
			Vital Registration_Single year	0
			adjvr_Single year	0
			Est.Vitales Anuarios y Enviada_Single year	0
			WHO Vital Registration Data 2011 version_Single year	1
Moldova	Demographic and Health Survey 2005_Direct	1	Demographic and Health Survey 2005_Direct	0
	Demographic and Health Survey 2005_Indirect	1	Demographic and Health Survey 2005_Indirect	0
	Census 1989_Indirect	1	Census 1989_Indirect	0
	WHO Vital Registration Data 2011 version_VR (Single year)	0	WHO Vital Registration Data 2011 version_VR (Single year)	0
Monaco	WHO Good Vital Registration Data 2011 version_VR (Single year)	1	WHO Good Vital Registration Data 2011 version_VR (Single year)	1
Mongolia	Reproductive Health Survey 2008_Indirect	1	Reproductive Health Survey 2008_Indirect	0
	Reproductive Health Survey 2008_Direct	0	Reproductive Health Survey 2008_Direct	0
	Multiple Indicator Cluster Survey 2005_Indirect	1	Multiple Indicator Cluster Survey 2005_Indirect	0
	Reproductive Health Survey 2003_Indirect	1	Reproductive Health Survey 2003_Indirect	0
	Reproductive Health Survey 2003_Direct	0	Reproductive Health Survey 2003_Direct	0
	Multiple Indicator Cluster Survey 2000_Indirect	1	Multiple Indicator Cluster Survey 2000_Indirect	0
	Reproductive Health Survey 1998_Indirect	1	Reproductive Health Survey 1998_Indirect	0
	Reproductive Health Survey 1998_Direct	1	Reproductive Health Survey 1998_Direct	0
	Demographic Survey 1996_Direct	1	Demographic Survey 1996_Direct	0
	Demographic Survey 1994_Direct	1	Demographic Survey 1994_Direct	0

	Census 1989_Indirect<br>WHO Vital Registration Data 2009 version_Single Year<br>WHO Vital Registration Data 2011 version_VR (Single year)	0<br>0<br>0	Census 1989_Indirect<br>Census 1989_Direct<br>WHO Vital Registration Data 2009 version_Single Year<br>WHO Vital Registration Data 2011 version_VR (Single year)	0<br>0<br>0<br>0
Montenegro	WHO Good Vital Registration Data 2011 version_VR (Single year)	1	WHO Good Vital Registration Data 2011 version_VR (Single year)	1
Montserrat	WHO Vital Registration Data 2011_VR (Single year)<br>The State of the World's Children (SOWC) 2009 Estimates_Single year	0<br>1	WHO Vital Registration Data 2011_VR (Single year)<br>The State of the World's Children (SOWC) 2009 Estimates_Single year	0<br>1
Morocco	National Demographic Survey with repeated passages 2009-2010_Single<br>year<br>National demographic survey 2009-10 Preliminary_Single year<br>Demographic and Health Survey 2003_Direct<br>Demographic and Health Survey 2003_Indirect<br>PAPG ENSME petit-echantillon 1997_Direct<br>PAPG ENSME grand-echantillon 1996_Direct<br>Demographic and Health Survey 1995_Direct<br>Demographic and Health Survey 1995_Indirect<br>Demographic and Health Survey 1992_Direct<br>Demographic and Health Survey 1992_Indirect<br>Demographic and Health Survey 1987_Direct<br>Demographic and Health Survey 1987_Indirect<br>Contraceptive Prevalence Survey 1983_Indirect<br>Census 1982_Indirect<br>World Fertility Survey 1980_Indirect<br>World Fertility Survey 1980_Direct	1<br>1<br>1<br>1<br>1<br>0<br>1<br>1<br>1<br>1<br>1<br>1<br>1<br>1<br>1<br>1	National Demographic Survey with repeated passages 2009-2010_Single year<br>National demographic survey 2009-10 Preliminary_Single year<br>Demographic and Health Survey 2003_Direct<br>Demographic and Health Survey 2003_Indirect<br>PAPG ENSME petit-echantillon 1997_Direct<br>PAPG ENSME grand-echantillon 1996_Direct<br>Demographic and Health Survey 1995_Direct<br>Demographic and Health Survey 1995_Indirect<br>Demographic and Health Survey 1992_Direct<br>Demographic and Health Survey 1992_Indirect<br>Demographic and Health Survey 1987_Direct<br>Demographic and Health Survey 1987_Indirect<br>Contraceptive Prevalence Survey 1983_Indirect<br>Census 1982_Indirect<br>World Fertility Survey 1980_Indirect<br>World Fertility Survey 1980_Direct	0<br>0<br>0<br>0<br>0<br>0<br>0<br>0<br>0<br>0<br>0<br>0<br>0<br>0<br>0<br>0
Mozambique	Multiple Cluster Indicator Survey 2008_Direct<br>Census 2007_Indirect<br>Demographic and Health Survey 2003_Direct<br>Demographic and Health Survey 2003_Indirect<br>Demographic and Health Survey 1997_Direct<br>Demographic and Health Survey 1997_Indirect<br>Census 1997_Indirect<br>Multiple Indicator Cluster Survey 1995_Indirect<br>Census 1980_Indirect<br>Census 1970_Indirect	1<br>1<br>1<br>1<br>0<br>0<br>1<br>1<br>1<br>0	Multiple Cluster Indicator Survey 2008_Direct<br>Census 2007_Indirect<br>Demographic and Health Survey 2003_Direct<br>Demographic and Health Survey 2003_Indirect<br>Demographic and Health Survey 1997_Direct<br>Demographic and Health Survey 1997_Indirect<br>Census 1997_Indirect<br>Multiple Indicator Cluster Survey 1995_Indirect<br>Census 1980_Indirect<br>Census 1970_Indirect	0<br>0<br>0<br>0<br>0<br>0<br>0<br>0<br>0<br>0
Myanmar	Fertility and Reproductive Health Survey 2007_Direct<br>Fertility and Reproductive Health Survey 2007_Indirect<br>Under-Five Mortality Survey 2002_Direct<br>Fertility and Reproductive Health Survey 2001_Direct<br>Fertility and Reproductive Health Survey 2001_Indirect<br>Fertility and Reproductive Health Survey 1997_Direct	1<br>1<br>0<br>1<br>1<br>1	Fertility and Reproductive Health Survey 2007_Direct<br>Fertility and Reproductive Health Survey 2007_Indirect<br>Under-Five Mortality Survey 2002_Direct<br>Fertility and Reproductive Health Survey 2001_Direct<br>Fertility and Reproductive Health Survey 2001_Indirect<br>Fertility and Reproductive Health Survey 1997_Direct	0<br>0<br>0<br>0<br>0<br>0

	Fertility and Reproductive Health Survey 1997_Indirect	1	Fertility and Reproductive Health Survey 1997_Indirect	0
	Population Change and Fertility Survey 1991_Direct	1	Population Change and Fertility Survey 1991_Direct	0
	Census 1983_Indirect	1	Census 1983_Indirect	0
Namibia	Demographic and Health Survey 2006_Direct	1	Demographic and Health Survey 2006_Direct	0
	Demographic and Health Survey 2006_Indirect	1	Demographic and Health Survey 2006_Indirect	0
	Census 2001_Single year	1	Census 2001_Single year	0
	Demographic and Health Survey 2000_Direct	1	Demographic and Health Survey 2000_Direct	0
	Demographic and Health Survey 2000_Indirect	1	Demographic and Health Survey 2000_Indirect	0
	Demographic and Health Survey 1992_Direct	1	Demographic and Health Survey 1992_Direct	0
	Demographic and Health Survey 1992_Indirect	1	Demographic and Health Survey 1992_Indirect	0
Nauru	Demographic and Health Survey 2007_Direct	1	Demographic and Health Survey 2007_Direct	0
	Vital Registration Data and Census_Single year	1	Vital Registration Data and Census_Single year	0
	WHO Vital Registration Data 2011 version_VR (Single year)	0	Vital Registration Data from Nauru Bureau of Statistics_Single year	0
			WHO Vital Registration Data 2011 version_VR (Single year)	0
Nepal	Demographic and Health Survey Preliminary 2011_Direct	1	Demographic and Health Survey Preliminary 2011_Direct	1
	Demographic and Health Survey 2006_Direct	1	Demographic and Health Survey 2006_Direct	1
	Demographic and Health Survey 2006_Indirect	1	Demographic and Health Survey 2006_Indirect	1
	Demographic and Health Survey 2001_Direct	1	Demographic and Health Survey 2001_Direct	1
	Demographic and Health Survey 2001_Indirect	1	Demographic and Health Survey 2001_Indirect	1
	Demographic and Health Survey 1996_Direct	1	Demographic and Health Survey 1996_Direct	1
	Demographic and Health Survey 1996_Indirect	1	Demographic and Health Survey 1996_Indirect	1
	Fertility and Family Planning Survey 1991_Direct	1	Fertility and Family Planning Survey 1991_Direct	1
	Fertility and Family Planning Survey 1991_Indirect	1	Fertility and Family Planning Survey 1991_Indirect	1
	Census 1991_Indirect	0	Census 1991_Indirect	0
	Fertility and Family Planning Survey 1985_Indirect	0	Fertility and Family Planning Survey 1985_Indirect	0
	Contraceptive Prevalence Survey 1981_Indirect	0	Contraceptive Prevalence Survey 1981_Indirect	0
	Census 1981_Indirect	0	Contraceptive Prevalence Survey 1981_Direct	0
	World Fertility Survey 1976_Direct	1	Census 1981_Indirect	0
	World Fertility Survey 1976_Indirect	1	World Fertility Survey 1976_Direct	1
	Census 1971_Indirect	0	World Fertility Survey 1976_Indirect	1
			Demographic Sample Survey 1974_Direct	0
			Census 1971_Indirect	0
			Fertility and Family Planning Survey 1985-86_Single year	0
Netherlands	WHO Good Vital Registration Data 2011 version_VR (Single year)	1	WHO Good Vital Registration Data 2011 version_VR (Single year)	1
New Zealand	WHO Good Vital Registration Data 2011 version_VR (Single year)	1	WHO Good Vital Registration Data 2011 version_VR (Single year)	1
Nicaragua	Demographic and Health Survey 2006/07_Direct	1	Demographic and Health Survey 2006/07_Direct	0
	Censo 2005_Indirect	1	Censo 2005_Indirect	0
	Demographic and Health Survey 2001_Direct	1	Demographic and Health Survey 2001_Direct	0
	Demographic and Health Survey 2001_Indirect	1	Demographic and Health Survey 2001_Indirect	0
	Demographic and Health Survey 1998_Direct	1	Demographic and Health Survey 1998_Direct	0

	Demographic and Health Survey 1998_Indirect	1	Demographic and Health Survey 1998_Indirect	0
	Censo 1995_Indirect	1	Censo 1995_Indirect	0
	Encuesta Sobre salud familiar Nicaragua 1992-93_Direct	1	Encuesta Sobre salud familiar Nicaragua 1992-93_Direct	0
	Encuesta Sobre salud familiar Nicaragua 1992-93_Indirect	1	Encuesta Sobre salud familiar Nicaragua 1992-93_Indirect	0
	Reproductive Health Survey 1992_Direct	1	Reproductive Health Survey 1992_Direct	0
	Enc.Socio-Demografica Nicaraguense 1985_Indirect	1	Enc.Socio-Demografica Nicaraguense 1985_Indirect	0
	Enc.Demografica Nacional 1978_Indirect	1	Enc.Demografica Nacional 1978_Indirect	0
	Censo 1971_Indirect	1	Censo 1971_Indirect	0
	WHO Vital Registration Data 2011 version_VR (Single year)	0	Est.Vitales Anuarios y Enviada_VR (Single year)	0
			WHO Vital Registration Data 2011 version_VR (Single year)	0
Niger	Child Survival and Mortality Survey 2010_Direct	0	Child Survival and Mortality Survey 2010_Direct	0
	Demographic and Health Survey 2006_Direct	1	Demographic and Health Survey 2006_Direct	0
	Demographic and Health Survey 2006_Indirect	0	Demographic and Health Survey 2006_Indirect	0
	Multiple Indicator Cluster Survey 2000_Indirect	1	Multiple Indicator Cluster Survey 2000_Indirect	0
	Demographic and Health Survey 1998_Direct	1	Demographic and Health Survey 1998_Direct	0
	Demographic and Health Survey 1998_Indirect	1	Demographic and Health Survey 1998_Indirect	0
	Multiple Indicator Cluster Survey 1996_Indirect	1	Multiple Indicator Cluster Survey 1996_Indirect	0
	Multiple Indicator Cluster Survey 1996_Direct	1	Multiple Indicator Cluster Survey 1996_Direct	0
	Demographic and Health Survey 1992_Direct	1	Demographic and Health Survey 1992_Direct	0
	Demographic and Health Survey 1992_Indirect	1	Demographic and Health Survey 1992_Indirect	0
	Survey 1960_Indirect	0	Survey 1960_Indirect	0
	Census 1988_Indirect	0	Census 1988_Indirect	0
	Census 2001_Indirect	0	Census 2001_Indirect	0
Nigeria	Demographic and Health Survey 2008_Direct	1	Demographic and Health Survey 2008_Direct	0
	Demographic and Health Survey 2008_Indirect	1	Demographic and Health Survey 2008_Indirect	0
	Multiple Indicator Cluster Survey 2007_Indirect	0	Multiple Indicator Cluster Survey 2007_Indirect	0
	Demographic and Health Survey 2003_Direct	1	Demographic and Health Survey 2003_Direct	0
	Demographic and Health Survey 2003_Indirect	1	Demographic and Health Survey 2003_Indirect	0
	Multiple Indicator Cluster Survey 1999_Indirect	0	Multiple Indicator Cluster Survey 1999_Indirect	0
	Demographic and Health Survey 1999_Direct	0	Demographic and Health Survey 1999_Direct	0
	Demographic and Health Survey 1999_Indirect	0	Demographic and Health Survey 1999_Indirect	0
	Multiple Indicator Cluster Survey 1995_Indirect	0	Multiple Indicator Cluster Survey 1995_Indirect	0
	Demographic and Health Survey 1990_Direct	1	Demographic and Health Survey 1990_Direct	0
	Demographic and Health Survey 1990_Indirect	1	Demographic and Health Survey 1990_Indirect	0
	World Fertility Survey 1981_Direct	0	Demographic and Health Survey 1986_Direct	0
	World Fertility Survey 1981_Indirect	0	World Fertility Survey 1981_Direct	0
	Malumfashi DSS 1962-1977_Single Year	0	World Fertility Survey 1981_Indirect	0
	Rural Survey 1965-1966_Single Year	1	Malumfashi DSS 1962-1977_Single Year	0
	Survey 1971-1973_Indirect	0	Rural Survey 1965-1966_Single Year	0
			Survey 1971-1973_Indirect	0

Niue	WHO Good Vital Registration Data 2011 version_VR (Single year)	0	WHO Good Vital Registration Data 2011 version_VR (Single year)	0
	WHO Good Vital Registration Data 2011 version Moving Average_VR (Single year)	1	WHO Good Vital Registration Data 2011 version Moving Average_VR (Single year)	0
Norway	WHO Good Vital Registration Data 2011 version_VR (Single year)	1	WHO Good Vital Registration Data 2011 version_VR (Single year)	1
Oman	Census 2003_Single year	0	Census 2003_Single year	1
	Census 2003_Indirect	0	Census 2003_Indirect	1
	Family Health Survey 1995_Direct	0	Family Health Survey 1995_Direct	1
	Family Health Survey 1995_Indirect	0	Family Health Survey 1995_Indirect	1
	Census 1993_Indirect	0	Census 1993_Indirect	1
	Child Health Survey 1988_Indirect	0	Child Health Survey 1988_Indirect	1
	Child Health Survey 1988_Direct	0	Child Health Survey 1988_Direct	1
	Data from Ministry of Health Annual Health Report_Single year	0	Data from Ministry of Health Annual Health Report_Single year	1
	Socio-Demographic Survey in 5 towns 1975_Indirect	0	Vital Registration_Single year	0
	Socio-Demographic Survey 1977-1979_Indirect	0	Socio-Demographic Survey in 5 towns 1975_Indirect	1
	Comprehensive Health Survey for Evaluation and Reproductive Health 2000_Indirect	0	Socio-Demographic Survey 1977-1979_Indirect	1
			Comprehensive Health Survey for Evaluation and Reproductive Health 2000_Indirect	1
OPT	Family Health Survey 2006_Direct	1	Family Health Survey 2006_Direct	1
	Family Health Survey 2006_Indirect	1	Family Health Survey 2006_Indirect	1
	Palestinian Family Health Survey 2006_Direct	1	Palestinian Family Health Survey 2006_Direct	1
	Demographic and Health Survey 2004_Direct	1	Demographic and Health Survey 2004_Direct	1
	Demographic and Health Survey 2004_Indirect	1	Demographic and Health Survey 2004_Indirect	1
	Health Survey 2000_Direct	1	Health Survey 2000_Direct	1
	Health Survey 2000_Indirect	1	Health Survey 2000_Indirect	1
	Census 1997_Indirect	1	Census 1997_Indirect	1
	Demographic Survey 1995_Direct	1	Demographic Survey 1995_Direct	1
	Demographic Survey 1995_Indirect	1	Demographic Survey 1995_Indirect	1
	FALCOT-survey 1992_Indirect	1	FALCOT-survey 1992_Indirect	1
	Health Survey 2000_Indirect	1	Health Survey 2000_Indirect	1
Pakistan	Demographic and Health Survey 2006_Direct	1	Pakistan Social and Living Standards Measurement Survey 2007-08_Direct	0
	Demographic and Health Survey 2006_Indirect	1	Demographic and Health Survey 2006_Direct	0
	Integrated Household Survey 2001_Direct	1	Demographic and Health Survey 2006_Indirect	0
	Integrated Household Survey 2001_Indirect	1	Demographic Survey 2005_Direct	0
	Reproductive Health and Family Planning 2000_Direct	1	Pakistan Social and Living Standards Measurement Survey 2005-06_Direct	0
	Integrated Household Survey 1998_Direct	1	Demographic Survey 2003_Direct	0
	Integrated Household Survey 1998_Indirect	1	Demographic Survey 2001_Direct	0
	Integrated Household Survey 1996_Indirect	1	Integrated Household Survey 2001_Direct	0
	Pakistan Fertility and Family Planning Survey 1996_Direct	1	Integrated Household Survey 2001_Indirect	0
	Pakistan Fertility and Family Planning Survey 1996_Indirect	1	Pakistan Integrated Household Survey 2001-2002_Direct	0
	Living Standards Survey 1991_Direct	1	Demographic Survey 2000_Direct	0

	Living Standards Survey 1991_Indirect	1	Reproductive Health and Family Planning 2000_Direct	0
	Demographic and Health Survey 1990_Direct	1	Integrated Household Survey 1998_Direct	0
	Demographic and Health Survey 1990_Indirect	1	Integrated Household Survey 1998_Indirect	0
	Demographic Survey 1988_Indirect	1	Integrated Household Survey 1996_Indirect	0
	Contraceptive Prevalence Survey 1984_Indirect	1	Integrated Household Survey 1996_Direct	0
	Demographic Survey 1984_Indirect	1	Pakistan Fertility and Family Planning Survey 1996_Direct	0
	Demographic Survey 1984_Direct	1	Pakistan Fertility and Family Planning Survey 1996_Indirect	0
	Census 1981_Indirect	1	Integrated Household Survey 1995_Direct	0
	Labour Force and Migration Survey 1980_Direct	1	Living Standards Survey 1991_Direct	0
	Population Growth Survey II 1976-1978_Direct	1	Living Standards Survey 1991_Indirect	0
	World Fertility Survey 1975_Direct	1	Demographic and Health Survey 1990_Direct	0
			Demographic and Health Survey 1990_Indirect	0
			Demographic Survey 1988_Indirect	0
			Contraceptive Prevalence Survey 1984_Indirect	0
			Demographic Survey 1984_Indirect	0
			Demographic Survey 1984_Direct	0
			Census 1981_Indirect	0
			Labour Force and Migration Survey 1980_Direct	0
			Population Growth Survey II 1976-1978_Direct	0
			World Fertility Survey 1975_Direct	0
			Population Growth Survey I 1968_Direct	0
			Population Growth Estimation Experiment 1962_Direct	0
Palau	Census 2005_Indirect	0	Census 2005_Indirect	0
	Census 2000_Indirect	0	Census 2000_Indirect	0
	Census 1995_Indirect	0	Census 1995_Indirect	0
	WHO Vital Registration Data 2011 version_VR (Single year)	1	WHO Vital Registration Data 2011 version_VR (Single year)	1
Panama	Census 2010_Indirect	1	Census 2010_Indirect	1
	Census 2000_Indirect	1	Census 2000_Indirect	1
	Census 1990_Indirect	1	Census 1990_Indirect	1
	Census 1980_Indirect	1	Census 1980_Indirect	1
	Encuesta Demografica Nacional 1976_Indirect	1	Encuesta Demografica Nacional 1976_Indirect	1
	Encuesta Nacional de Fecundidad 1975_Direct	1	Encuesta Nacional de Fecundidad 1975_Direct	1
	Encuesta Demografica Nacional 1975_Direct	1	Encuesta Demografica Nacional 1975_Direct	1
	Vital Registration_Single year	0	Vital Registration_Single year	0
	Vital Registration & Census_Single year	0	Est.Vitales Anuarios y Enviada_Single year	0
	WHO Vital Registration Data 2009 version_Single year	0	Vital Registration & Census_Single year	0
	TABLAS DE VIDA NACIONALES_Single year	1	WHO Vital Registration Data 2009 version_Single year	0
	WHO Vital Registration Data 2011 version_VR (Single year)	0	TABLAS DE VIDA NACIONALES_Single year	1
			WHO Vital Registration Data 2011 version_VR (Single year)	0
Papua New	Demographic and Health Survey 2006_Direct	1	Demographic and Health Survey 2006_Direct	0

Guinea	Census 2000_Indirect	1	Census 2000_Indirect	0
	Demographic and Health Survey 1996_Direct	1	Demographic and Health Survey 1996_Direct	0
	Demographic and Health Survey 1996_Indirect	1	Demographic and Health Survey 1996_Indirect	0
	Demographic and Health Survey 1991_Indirect	1	Demographic and Health Survey 1991_Indirect	0
	Census 1980_Indirect	0	Census 1980_Indirect	0
	Census 1971_Indirect	1	Census 1971_Indirect	0
	WHO Vital Registration Data 2009 version_Single Year	0	WHO Vital Registration Data 2009 version_Single Year	0
	WHO Vital Registration Data 2011 version_VR (Single year)	0	WHO Vital Registration Data 2011 version_VR (Single year)	0
Paraguay	ENDSSR 2008_Direct	1	ENDSSR 2008_Direct	0
	Reproductive Health Survey 2004_Direct	1	Reproductive Health Survey 2004_Direct	0
	Census 2002_Indirect	1	Census 2002_Indirect	0
	ENDSR 1995_Direct	1	ENDSR 1995_Direct	0
	ENDSR 1995_Indirect	1	ENDSR 1995_Indirect	0
	Census 1992_Indirect	1	Census 1992_Indirect	0
	Demographic and Health Survey 1990_Direct	1	Demographic and Health Survey 1990_Direct	0
	Demographic and Health Survey 1990_Indirect	1	Demographic and Health Survey 1990_Indirect	0
	Census 1982_Indirect	1	Census 1982_Indirect	0
	Encuesta Nacional de Fecundidad 1979_Indirect	1	Encuesta Nacional de Fecundidad 1979_Indirect	0
	Encuesta Nacional de Fecundidad 1979_Direct	1	Encuesta Nacional de Fecundidad 1979_Direct	0
	Encuesta Demografica Nacional 1977_Indirect	1	Encuesta Demografica Nacional 1977_Indirect	0
	Census 1972_Indirect	1	Census 1972_Indirect	0
	WHO Vital Registration Data 2009 version_Single Year	0	Estadisticas Vitales Anuarios Estadisticos _Single year	0
	VR Ministerio de Salud Pulbica y Bienestar Social - Departamento de	0	WHO Vital Registration Data 2009 version_Single Year	0
	Bioestadisiticas 2008 2009_VR (Single year)		VR Ministerio de Salud Pulbica y Bienestar Social - Departamento de	0
	WHO Vital Registration Data 2011 version_VR (Single year)	0	Bioestadisiticas 2008 2009_VR (Single year)	0
			WHO Vital Registration Data 2011 version_VR (Single year)	0
Peru	Encuesta Demografica y de Salud Familiar 2010-ENDES Continua_Direct	1	Encuesta Demografica y de Salud Familiar 2010-ENDES Continua_Direct	0
	Encuesta Demografica y de Salud Familiar 2009_Direct	1	Encuesta Demografica y de Salud Familiar 2009_Direct	0
	Encuesta Demografica y de Salud Familiar ENDES Continua 2007-2008_Direct	1	Encuesta Demografica y de Salud Familiar ENDES Continua 2007-2008_Direct	0
	Census 2007_Indirect	1	Census 2007_Indirect	0
	Demographic and Health Survey 2004-2006_Indirect	1	Demographic and Health Survey 2004-2006_Indirect	0
	Demographic and Health Survey 2004-2006_Direct	1	Demographic and Health Survey 2004-2006_Direct	0
	Demographic and Health Survey 2004_Direct	1	Demographic and Health Survey 2004_Direct	0
	Demographic and Health Survey 2004_Indirect	1	Demographic and Health Survey 2004_Indirect	0
	Demographic and Health Survey 2000_Direct	1	Demographic and Health Survey 2000_Direct	0
	Demographic and Health Survey 2000_Indirect	1	Demographic and Health Survey 2000_Indirect	0
	Demographic and Health Survey 1996_Direct	1	Demographic and Health Survey 1996_Direct	0
	Demographic and Health Survey 1996_Indirect	1	Demographic and Health Survey 1996_Indirect	0
	Census 1993_Indirect	1	Census 1993_Indirect	0
	Demographic and Health Survey 1991_Direct	1	Demographic and Health Survey 1991_Direct	0

	Demographic and Health Survey 1991_Indirect	1	Demographic and Health Survey 1991_Indirect	0
	Demographic and Health Survey 1986_Direct	1	Demographic and Health Survey 1986_Direct	0
	Demographic and Health Survey 1986_Indirect	1	Demographic and Health Survey 1986_Indirect	0
	Census 1981_Indirect	1	Census 1981_Indirect	0
	Contraceptive Prevalence Survey 1981_Indirect	1	Contraceptive Prevalence Survey 1981_Indirect	0
	World Fertility Survey 1977_Direct	1	World Fertility Survey 1977_Direct	0
	World Fertility Survey 1977_Indirect	1	World Fertility Survey 1977_Indirect	0
	Demographic Survey 1976_Indirect	1	Demographic Survey 1976_Indirect	0
	Demographic Survey 1974_Direct	1	Demographic Survey 1974_Direct	0
	Census 1972_Indirect	1	Census 1972_Indirect	0
	Vital Registration (26 March 2009)_Single year	0	Vital Registration (26 March 2009)_Single year	0
	WHO Vital Registration Data 2009 version_Single Year	0	WHO Vital Registration Data 2009 version_Single Year	0
	WHO Vital Registration Data 2011 version_VR (Single year)	0	WHO Vital Registration Data 2011 version_VR (Single year)	0
Philippines	Demographic and Health Survey 2008_Direct	1	Demographic and Health Survey 2008_Direct	0
	Demographic and Health Survey 2008_Indirect	1	Demographic and Health Survey 2008_Indirect	0
	Family Planning Survey 2006_Direct	0	Family Planning Survey 2006_Direct	0
	Family Planning Survey 2006_Indirect	1	Family Planning Survey 2006_Indirect	0
	Demographic and Health Survey 2003_Direct	1	Demographic and Health Survey 2003_Direct	0
	Demographic and Health Survey 2003_Indirect	1	Demographic and Health Survey 2003_Indirect	0
	Demographic and Health Survey 1998_Direct	1	Demographic and Health Survey 1998_Direct	0
	Demographic and Health Survey 1998_Indirect	1	Demographic and Health Survey 1998_Indirect	0
	Demographic and Health Survey 1993_Direct	1	Demographic and Health Survey 1993_Direct	0
	Demographic and Health Survey 1993_Indirect	1	Demographic and Health Survey 1993_Indirect	0
	Demographic Survey 1988_Indirect	1	Demographic Survey 1988_Indirect	0
	Census 1980_Indirect	0	Census 1980_Indirect	0
	World Fertility Survey 1978_Direct	1	World Fertility Survey 1978_Direct	0
	Census 1970_Indirect	0	Census 1970_Indirect	0
	WHO Vital Registration Data 2009 version_VR (Single year)	0	WHO Vital Registration Data 2009 version_VR (Single year)	0
	WHO Vital Registration Data 2011 version_VR (Single year)	0	WHO Vital Registration Data 2011 version_VR (Single year)	0
Poland	WHO Good Vital Registration Data 2011 version_VR (Single year)	1	WHO Good Vital Registration Data 2011 version_VR (Single year)	1
Portugal	WHO Good Vital Registration Data 2011 version_VR (Single year)	1	WHO Good Vital Registration Data 2011 version_VR (Single year)	1
Qatar	GFH 1998_Direct	0	GFH 1998_Direct	0
	GFH 1998_Indirect	0	GFH 1998_Indirect	0
	Child Health Survey 1987_Direct	1	Child Health Survey 1987_Direct	1
	Child Health Survey 1987_Indirect	1	Child Health Survey 1987_Indirect	1
	Census 2004_Indirect	0	Census 2004_Indirect	0
	WHO Good Vital Registration Data 2011 version_Single year	1	WHO Good Vital Registration Data 2011 version_Single year	1
Romania	Reproductive Health Survey-cdc 1999_Direct	0	Reproductive Health Survey-cdc 1999_Direct	0
	WHO Good Vital Registration Data 2011 version_VR (Single year)	0	WHO Good Vital Registration Data 2011 version_VR (Single year)	0
	WHO Good VR 2011 adjusted version by increasing 20%_VR (Single year)	1	WHO Good VR 2011 adjusted version by increasing 20%_VR (Single year)	1

Russian Federation	WHO VR 2011 adjusted version by increasing 20%_Single year	1	WHO VR 2011 adjusted version by increasing 20%_Single year	1
	Census 1989_Indirect	1	Census 1989_Indirect	1
	Trans MONEE Vital Registration_VR (Single year)	0	Trans MONEE Vital Registration_VR (Single year)	0
	WHO Vital Registration 2008 Version_VR (Single year)	0	WHO Vital Registration 2008 Version_VR (Single year)	0
	WHO Good Vital Registration Data 2009 version_VR (Single year)	0	WHO Good Vital Registration Data 2009 version_VR (Single year)	0
	WHO Good Vital Registration Data 2010 version_VR (Single year)	0	WHO Good Vital Registration Data 2010 version_VR (Single year)	0
	WHO Vital Registration Data 2011 version_VR (Single year)	0	WHO Vital Registration Data 2011 version_VR (Single year)	0
Rwanda	Interim Demographic and Health Survey 2008_Direct	0	Interim Demographic and Health Survey 2008_Direct	0
	Demographic and Health Survey 2008_Indirect	1	Demographic and Health Survey 2008_Indirect	0
	Demographic and Health Survey 2008_Direct	1	Demographic and Health Survey 2008_Direct	0
	Demographic and Health Survey 2005_Indirect	1	Demographic and Health Survey 2005_Indirect	0
	Demographic and Health Survey 2005_Direct	1	Demographic and Health Survey 2005_Direct	0
	Demographic and Health Survey 2000_Indirect	1	Demographic and Health Survey 2000_Indirect	0
	Demographic and Health Survey 2000_Direct	1	Demographic and Health Survey 2000_Direct	0
	Socio-demographic Survey 1996_Indirect	1	Socio-demographic Survey 1996_Indirect	0
	Demographic and Health Survey 1992_Indirect	1	Demographic and Health Survey 1992_Indirect	0
	Demographic and Health Survey 1992_Direct	1	Demographic and Health Survey 1992_Direct	0
	National Fertility Survey 1983_Indirect	1	National Fertility Survey 1983_Indirect	0
	National Fertility Survey 1983_Direct	1	National Fertility Survey 1983_Direct	0
	Census 1978_Indirect	1	Census 1978_Indirect	0
	Demographic Survey 1970_Indirect	1	Demographic Survey 1970_Indirect	0
Saint Kitts & Nevis	WHO Good Vital Registration Data 2011 version_VR (Single year)	1	WHO Good Vital Registration Data 2011 version_VR (Single year)	1
Saint Lucia	WHO Good Vital Registration Data 2011 version_VR (Single year)	1	WHO Good Vital Registration Data 2011 version_VR (Single year)	1
Samoa	Demographic and Health Survey 2009_Direct	0	Demographic and Health Survey 2009_Direct	0
	Demographic and Health Survey 2009_Indirect	0	Demographic and Health Survey 2009_Indirect	0
	Population and Housing Census 2006_Indirect	1	Population and Housing Census 2006_Indirect	0
	Population and Housing Census 2001_Indirect	1	Population and Housing Census 2006_Single year	0
	Demographic and Vital Statistics Survey 2000_Indirect	1	Population and Housing Census 2001_Indirect	0
	Demographic and Health Survey 1999_Indirect	1	Population and Housing Census 2001_Single year	0
	Demographic and Health Survey 1999_Single year	1	Demographic and Vital Statistics Survey 2000_Indirect	0
			Demographic and Vital Statistics Survey 2000_ Single year	0
			Demographic and Health Survey 1999_Indirect	0
			Demographic and Health Survey 1999_Single year	0
San Marino	WHO Good Vital Registration Data 2011 version_VR (Single year)	0	WHO Good Vital Registration Data 2011 version_VR (Single year)	0
	WHO Good Vital Registration Data 2011 version Moving Average_VR (Single year)	1	WHO Good Vital Registration Data 2011 version Moving Average_VR (Single year)	0
Sao Tome & Principe	Demographic and Health Survey 2008_Direct	1	Demographic and Health Survey 2008_Direct	0
	Demographic and Health Survey 2008_Indirect	1	Demographic and Health Survey 2008_Indirect	0
	Multiple Indicator Cluster Survey 2006_Indirect	0	Multiple Indicator Cluster Survey 2006_Indirect	0

	Multiple Indicator Cluster Survey 2000_Indirect<br>Census 1991_Indirect<br>Census 1980_Indirect<br>WHO Vital Registration Data 2009 version_Single Year<br>WHO Vital Registration Data 2011 version_VR (Single year)	1<br>1<br>1<br>0<br>0	Multiple Indicator Cluster Survey 2000_Indirect<br>Census 1991_Indirect<br>Census 1980_Indirect<br>WHO Vital Registration Data 2009 version_Single Year<br>WHO Vital Registration Data 2011 version_VR (Single year)	0<br>0<br>0<br>0<br>0
Saudi Arabia	Gulf Family Health Survey 1996_Direct<br>Child Health Survey 1987_Indirect	0<br>0	Census 2004_Indirect<br>Gulf Family Health Survey 1996_Direct<br>Child Health Survey 1987_Indirect<br>Vital Registration from UNSD_Single year	1<br>0<br>1<br>1
Senegal	Demographic and Health Survey 2010-2011 Preliminary_Direct<br>Malaria Indiactor Survey 2008_Direct<br>Malaria Indiactor Survey 2008_Indirect<br>Demographic and Health Survey 2005_Direct<br>Demographic and Health Survey 2005_Indirect<br>Demographic and Health Survey 1999_Direct<br>Demographic and Health Survey 1999_Indirect<br>Demographic and Health Survey 1997_Direct<br>Demographic and Health Survey 1997_Indirect<br>Multiple Indicator Cluster Survey 1996_Indirect<br>Demographic and Health Survey 1992_Direct<br>Demographic and Health Survey 1992_Indirect<br>Demographic and Health Survey 1986_Direct<br>Demographic and Health Survey 1986_Indirect<br>Multiround Survey 1978-1979_Single year<br>World Fertility Survey 1978_Direct<br>World Fertility Survey 1978_Indirect<br>Multiround Survey 1970-1971_Single year<br>Survey 1960-1961_Indirect	1<br>1<br>1<br>1<br>1<br>1<br>1<br>1<br>1<br>1<br>1<br>1<br>1<br>1<br>1<br>1<br>1<br>1<br>1	Demographic and Health Survey 2010-2011 Preliminary_Direct<br>Malaria Indiactor Survey 2008_Direct<br>Malaria Indiactor Survey 2008_Indirect<br>Demographic and Health Survey 2005_Direct<br>Demographic and Health Survey 2005_Indirect<br>Demographic and Health Survey 1999_Direct<br>Demographic and Health Survey 1999_Indirect<br>Demographic and Health Survey 1997_Direct<br>Demographic and Health Survey 1997_Indirect<br>Multiple Indicator Cluster Survey 1996_Indirect<br>Demographic and Health Survey 1992_Direct<br>Demographic and Health Survey 1992_Indirect<br>Demographic and Health Survey 1986_Direct<br>Demographic and Health Survey 1986_Indirect<br>Multiround Survey 1978-1979_Single year<br>World Fertility Survey 1978_Direct<br>World Fertility Survey 1978_Indirect<br>Multiround Survey 1970-1971_Single year<br>Survey 1960-1961_Indirect<br>Survey 1960-1961_Direct	0<br>0<br>0<br>0<br>0<br>0<br>0<br>0<br>0<br>0<br>0<br>0<br>0<br>0<br>0<br>0<br>0<br>0<br>0<br>0
Serbia	WHO Good Vital Registration Data 2011 version_VR (Single year)	1	WHO Good Vital Registration Data 2011 version_VR (Single year)	1
Seychelles	Census 1971_Indirect<br>Census 1960_Indirect<br>WHO Good Vital Registration Data 2011 version_Single year	1<br>1<br>1	Census 1971_Indirect<br>Census 1960_Indirect<br>WHO Good Vital Registration Data 2011 version_Single year	1<br>1<br>1
Sierra Leone	Demographic and Health Survey 2008_Direct<br>Demographic and Health Survey 2008_Indirect<br>Multiple Indicator Cluster Survey 2005_Indirect<br>Census 2004_Indirect<br>Census 2004_ Single year<br>Pilot Census 2003_Indirect<br>Multiple Indicator Cluster Survey 2000_Indirect	0<br>0<br>1<br>1<br>1<br>0<br>1	Demographic and Health Survey 2008_Direct<br>Demographic and Health Survey 2008_Indirect<br>Multiple Indicator Cluster Survey 2005_Indirect<br>Census 2004_Indirect<br>Census 2004_ Single year<br>Pilot Census 2003_Indirect<br>Multiple Indicator Cluster Survey 2000_Indirect	0<br>0<br>0<br>0<br>0<br>0<br>0

	DSMS 1992_Single year<br>DSMS 1992_Indirect<br>Census 1985_Indirect<br>National Survey 1977_Single year<br>Census 1974_Indirect<br>Pilot Census 1973_Indirect<br>National Fertility Survey 1969_Indirect	1<br>0<br>1<br>1<br>1<br>1<br>0	DSMS 1992_Single year<br>DSMS 1992_Indirect<br>Census 1985_Indirect<br>Census 1974_Indirect<br>Pilot Census 1973_Indirect<br>National Fertility Survey 1969_Indirect	0<br>0<br>0<br>0<br>0<br>0
Singapore	WHO Good Vital Registration Data 2011 version_VR (Single year)	1	WHO Good Vital Registration Data 2011 version_VR (Single year)	1
Slovakia	WHO Good VR 2011 adjusted version by increasing 20%_VR (Single year)<br>WHO Good Vital Registration Data 2011 version_VR (Single year)	1<br>0	WHO Good VR 2011 adjusted version by increasing 20%_VR (Single year)<br>WHO Good Vital Registration Data 2011 version_VR (Single year)	1<br>0
Slovenia	WHO Good Vital Registration Data 2011 version_VR (Single year)	1	WHO Good Vital Registration Data 2011 version_VR (Single year)	1
Solomon Islands	Demographic and Health Survey 2007_Direct<br>Demographic and Health Survey 2007_Indirect<br>Census 1999_Indirect<br>Census 1986_Indirect<br>Census 1976_Indirect	1<br>1<br>1<br>1<br>1	Demographic and Health Survey 2007_Direct<br>Demographic and Health Survey 2007_Indirect<br>Census 1999_Indirect<br>Census 1986_Indirect<br>Census 1976_Indirect	0<br>0<br>0<br>0<br>0
Somalia	Multiple Indicator Cluster Survey 2006_Direct<br>Multiple Indicator Cluster Survey 2006_Indirect<br>Multiple Indicator Cluster Survey 1999_Indirect	1<br>1<br>1	Multiple Indicator Cluster Survey 2006_Direct<br>Multiple Indicator Cluster Survey 2006_Indirect<br>Multiple Indicator Cluster Survey 1999_Indirect	0<br>0<br>0
South Africa	Community Survey 2007_Single year<br>Demographic and Health Survey 2004_Direct<br>Census 2001_Indirect<br>Census 2001_Single year<br>Demographic and Health Survey 1998_Direct<br>Demographic and Health Survey 1998_Indirect<br>HSRC 1990_Single year<br>WHO Vital Registration_Single year<br>WHO Vital Registration Data 2009 version_Single Year<br>WHO Vital Registration Data 2011 version_VR (Single year)	1<br>0<br>1<br>1<br>1<br>1<br>1<br>0<br>0<br>0	Demographic and Health Survey 2004_Direct<br>Vital Statistics 2002_Single year<br>Demographic and Health Survey 1998_Direct<br>Demographic and Health Survey 1998_Indirect<br>HSRC 1990_Single year<br>WHO Vital Registration_Single year<br>WHO Vital Registration Data 2009 version_Single Year<br>WHO Vital Registration Data 2011 version_VR (Single year	0<br>0<br>0<br>0<br>0<br>0<br>0<br>0
Spain	WHO Good Vital Registration Data 2011 version_VR (Single year)<br>WHO Good VR 2011 adjusted version by increasing 20%_VR (Single year)	0<br>1	WHO Good Vital Registration Data 2011 version_VR (Single year)<br>WHO Good VR 2011 adjusted version by increasing 20%_VR (Single year)	0<br>1
Sri Lanka	Demographic and Health Survey 2006_Direct<br>Demographic and Health Survey 2006_Indirect<br>Demographic and Health Survey 2001_Indirect<br>Demographic and Health Survey 2000_Direct<br>Demographic and Health Survey 1993_Direct<br>Demographic and Health Survey 1987_Direct<br>Demographic and Health Survey 1987_Indirect<br>World Fertility Survey 1975_Indirect<br>World Fertility Survey 1975_Direct	1<br>1<br>0<br>1<br>1<br>1<br>1<br>1<br>1	Demographic and Health Survey 2006_Direct<br>Demographic and Health Survey 2006_Indirect<br>Demographic and Health Survey 2001_Indirect<br>Demographic and Health Survey 2000_Direct<br>Demographic and Health Survey 1993_Direct<br>Demographic and Health Survey 1987_Direct<br>Demographic and Health Survey 1987_Indirect<br>World Fertility Survey 1975_Indirect<br>World Fertility Survey 1975_Direct	0<br>0<br>0<br>0<br>0<br>0<br>0<br>0<br>0

	Census 1971_Indirect<br>National life table_Single year<br>WHO Vital Registration Data 2009 version_Single Year<br>WHO Vital Registration Data 2011 version_VR (Single year)	1<br>1<br>0<br>0	Census 1971_Indirect<br>Vital Registration UN Statistic Division_Single year<br>National life table_Single year<br>WHO Vital Registration Data 2009 version_Single Year<br>WHO Vital Registration Data 2011 version_VR (Single year)	0<br>0<br>0<br>0<br>0
St Vincent & the Grenadines	WHO Good Vital Registration Data 2011 version_VR (Single year)<br>WHO Good Vital Registration Data 2011 version Moving Average_VR (Single year)	1<br>0	WHO Good Vital Registration Data 2011 version_VR (Single year)<br>WHO Good Vital Registration Data 2011 version Moving Average_VR (Single year)	1<br>0
Sudan	Census 2008_Indirect<br>Multiple Indicator Cluster Survey 2006_Indirect<br>Multiple Indicator Cluster Survey 2006_Direct<br>Pan Arab for Child Development Survey 1993_Direct<br>Census 1993_Indirect<br>Demographic and Health Survey 1989_Direct<br>Demographic and Health Survey 1989_Indirect<br>Census 1983_Indirect<br>Census 1973_Indirect<br>Census 1955-56_Single year	1<br>1<br>1<br>1<br>1<br>1<br>1<br>1<br>1<br>1	Census 2008_Indirect<br>Multiple Indicator Cluster Survey 2006_Indirect<br>Multiple Indicator Cluster Survey 2006_Direct<br>Pan Arab for Child Development Survey 1993_Direct<br>Census 1993_Indirect<br>Demographic and Health Survey 1989_Direct<br>Demographic and Health Survey 1989_Indirect<br>Census 1983_Indirect<br>Census 1973_Indirect<br>Census 1955-56_Single year	0<br>0<br>0<br>0<br>0<br>0<br>0<br>0<br>0<br>0
Suriname	Multiple Indicator Cluster Survey 2006_Indirect<br>Multiple Indicator Cluster Survey 2000_Indirect<br>WHO Vital Registration Data 2011 version_VR (Single year)<br>WHO Vital Registration Data 2011 version Moving Average_VR (Single year)	1<br>1<br>0<br>0	Multiple Indicator Cluster Survey 2006_Indirect<br>Multiple Indicator Cluster Survey 2000_Indirect<br>WHO Vital Registration Data 2011 version_VR (Single year)<br>WHO Vital Registration Data 2011 version Moving Average_VR (Single year)	0<br>0<br>0<br>0
Swaziland	Demographic and Health Survey 2007_Direct<br>Census 2007_Indirect<br>Demographic and Health Survey 2006_Direct<br>Demographic and Health Survey 2006_Indirect<br>Multiple Indicator Cluster Survey 2000_Indirect<br>Census 1997_Indirect<br>Census 1986_Indirect<br>Census 1976_Indirect<br>Census 1966_Indirect	1<br>1<br>0<br>1<br>1<br>1<br>1<br>1<br>1	Demographic and Health Survey 2006_Direct<br>Demographic and Health Survey 2006_Indirect<br>Multiple Indicator Cluster Survey 2000_Indirect<br>Census 1997_Indirect<br>Census 1986_Indirect<br>Census 1976_Indirect<br>Census 1966_Indirect	0<br>0<br>0<br>0<br>0<br>0<br>0
Sweden	WHO Good Vital Registration Data 2011 version_VR (Single year)	1	WHO Good Vital Registration Data 2011 version_VR (Single year)	1
Switzerland	WHO Good Vital Registration Data 2011 version_VR (Single year)	1	WHO Good Vital Registration Data 2011 version_VR (Single year)	1
Syria	Family Health Survey (PAPFAM) 2009_Direct<br>Health survey for causes of child deaths 2007-2008_Single year<br>Multiple Indicator Cluster Survey 2006_Indirect<br>Population and Housing Census 2004_Single year<br>Family Health Survey (PAPFAM) 2001_Direct<br>Multiple Indicator Cluster Survey 2000_Indirect<br>Census 1994_Indirect	1<br>1<br>1<br>1<br>1<br>1<br>1	Family Health Survey (PAPFAM) 2009_Direct<br>Health survey for causes of child deaths 2007-2008_Single year<br>Multiple Indicator Cluster Survey 2006_Indirect<br>Population and Housing Census 2004_Single year<br>Family Health Survey (PAPFAM) 2001_Direct<br>Multiple Indicator Cluster Survey 2000_Indirect<br>Census 1994_Indirect	0<br>0<br>0<br>0<br>0<br>0<br>0

	Family Health Survey (PAPFAM) 1993_Direct	1	Family Health Survey (PAPFAM) 1993_Direct	0
	EPI/CDD and Child Mortality Survey 1990_Indirect	1	EPI/CDD and Child Mortality Survey 1990_Indirect	0
	Census 1981_Indirect	1	Census 1981_Indirect	0
	World Fertility Survey 1978_Direct	1	World Fertility Survey 1978_Direct	0
	World Fertility Survey 1978_Indirect	1	World Fertility Survey 1978_Indirect	0
	Census 1976_Indirect	1	Demographic Survey 1978_Direct	0
	Census 1970_Indirect	1	Census 1976_Indirect	0
			Census 1970_Indirect	0
			Vital Registeration_Single year	0
Tajikistan	Survey on Infant, Child and Maternal Mortality 2010_Indirect	0	Survey on Infant, Child and Maternal Mortality 2010_Indirect	0
	Survey on Infant, Child and Maternal Mortality 2010_Single year	0	Survey on Infant, Child and Maternal Mortality 2010_Single year	0
	Tajikistan Living Standards Survey 2007_Indirect	0	Tajikistan Living Standards Survey 2007_Indirect	0
	Tajikistan Living Standards Survey 2007_Direct	0	Tajikistan Living Standards Survey 2007_Direct	0
	Multiple Indicator Cluster Survey 2005_Indirect	1	Multiple Indicator Cluster Survey 2005_Indirect	0
	Living Standards Survey (tls) 2003_Indirect	0	Living Standards Survey (tls) 2003_Indirect	0
	Living Standards Survey (tls) 2003_Direct	0	Living Standards Survey (tls) 2003_Direct	0
	Demographic Survey 2002_Direct	1	Demographic Survey 2002_Direct	0
	Demographic Survey 2002_Indirect	1	Demographic Survey 2002_Indirect	0
	Multiple Indicator Cluster Survey 2000_Indirect	1	Multiple Indicator Cluster Survey 2000_Indirect	0
	Living Standards Survey (tls) 1999_Indirect	1	Living Standards Survey (tls) 1999_Indirect	0
	Living Standards Survey (tls) 1999_Direct	1	Living Standards Survey (tls) 1999_Direct	0
	Census 1989_Indirect	1	Census 1989_Indirect	0
	WHO Vital Registration Data 2011 version_VR (Single year)	0	UNSD Vital Registration_Single year	0
			WHO Vital Registration Data 2011 version_VR (Single year)	0
Tanzania	Demographic and Health Survey 2010_Direct	1	Demographic and Health Survey 2010_Direct	0
	HIV AIDS and Malaria Indicator Survey 2007-08_Direct	1	HIV AIDS and Malaria Indicator Survey 2007-08_Direct	0
	HIV AIDS and Malaria Indicator Survey 2007-08_Indirect	1	HIV AIDS and Malaria Indicator Survey 2007-08_Indirect	0
	Demographic and Health Survey 2004_Direct	1	Demographic and Health Survey 2004_Direct	0
	Census Preliminary 2002_Single year	1	Census Preliminary 2002_Single year	0
	Census 2002_Indirect	1	Census 2002_Indirect	0
	Demographic and Health Survey 1999_Direct	1	Demographic and Health Survey 1999_Direct	0
	Demographic and Health Survey 1999_Indirect	1	Demographic and Health Survey 1999_Indirect	0
	Demographic and Health Survey 1996_Direct	1	Demographic and Health Survey 1996_Direct	0
	Demographic and Health Survey 1996_Indirect	1	Demographic and Health Survey 1996_Indirect	0
	Demographic and Health Survey 1991_Direct	1	Demographic and Health Survey 1991_Direct	0
	Demographic and Health Survey 1991_Indirect	1	Demographic and Health Survey 1991_Indirect	0
	Census 1988_Indirect	1	Census 1988_Indirect	0
	Census 1978_Indirect	1	Census 1978_Indirect	0
	Census 1967_Indirect	1	Census 1967_Indirect	0

Thailand	Multiple Indicator Cluster Survey 2005_Indirect	1	Multiple Indicator Cluster Survey 2005_Indirect	0
	Survey of Population Change 2005-06_Indirect	1	Survey of Population Change 2005-06_Direct	0
	Survey of Population Change 2005-06_Direct	1	Life Table in 2002_Single year	0
	Life Table in 2002_Single year	1	Census 2000_Indirect	0
	Census 2000_Indirect	1	Life Table 1990-2000 Intercensal Death_Single year	0
	Contraceptive Prevalence Survey 1996_Indirect	1	Survey of Population Change 1995_Direct	0
	Life Table 1990-2000 Intercensal Death_Single year	1	Census 1990_Indirect	0
	Survey of Population Change 1995_Indirect	1	Survey of Population Change 1989_Indirect	0
	Survey of Population Change 1995_Direct	1	Demographic and Health Survey 1987_Direct	0
	Census 1990_Indirect	0	Demographic and Health Survey 1987_Indirect	0
	Survey of Population Change 1989_Indirect	1	Survey of Population Change 1985-86_Indirect	0
	Survey of Population Change 1989_Direct	1	Life Table1980-1990 Intercensal Death_Single year	0
	Demographic and Health Survey 1987_Direct	1	Survey of Population Change 1985-86_Direct	0
	Demographic and Health Survey 1987_Indirect	1	Contraceptive Prevalence Survey 1984_Indirect	0
	Survey of Population Change 1985-86_Indirect	1	Contraceptive Prevalence Survey 1981_Indirect	0
	Life Table1980-1990 Intercensal Death_Single year	1	Census 1980_Indirect	0
	Survey of Population Change 1985-86_Direct	1	National Fertility Survey 1979_Indirect	0
	Contraceptive Prevalence Survey 1984_Indirect	1	World Fertility Survey 1975_Direct	0
	Contraceptive Prevalence Survey 1981_Indirect	1	World Fertility Survey 1975_Indirect	0
	Census 1980_Indirect	1	Survey of Population Change 1974-75_Indirect	0
	National Fertility Survey 1979_Indirect	1	Survey of Population Change 1974-75_Direct	0
	World Fertility Survey 1975_Direct	1	Census 1970_Indirect	0
	World Fertility Survey 1975_Indirect	1	Vital Registraion_Single year	0
	Survey of Population Change 1974-75_Indirect	1	National Life Tables_Single year	0
	Survey of Population Change 1974-75_Direct	1	WHO Vital Registration Data 2009 version_Single Year	0
	Census 1970_Indirect	1	WHO Vital Registration Data 2011 version_VR (Single year)	0
	Vital Registraion_Single year	0		
	National Life Tables_Single year	1		
	WHO Vital Registration Data 2009 version_Single Year	0		
	WHO Vital Registration Data 2011 version_VR (Single year)	0		
Timor Leste	Demographic and Health Survey 2009_Direct	1	Demographic and Health Survey 2009_Direct	0
	Census 2004_Indirect	1	Census 2004_Indirect	0
	Demographic and Health Survey 2003_Direct	1	Demographic and Health Survey 2003_Direct	0
	Multiple Indicator Cluster Survey 2002_Indirect	1	Multiple Indicator Cluster Survey 2002_Indirect	0
	Living Standards Survey 2001_Indirect	1	Living Standards Survey 2001_Indirect	0
	ICS 1995_Indirect	0	ICS 1995_Indirect	0
	Census 1990_Indirect	0	Census 1990_Indirect	0
Togo	Multiple Indicator Cluster Survey 2006_Indirect	1	Multiple Indicator Cluster Survey 2006_Indirect	1
	Demographic and Health Survey 1998_Direct	1	Demographic and Health Survey 1998_Direct	1
	Demographic and Health Survey 1998_Indirect	1	Demographic and Health Survey 1998_Indirect	1

	Demographic and Health Survey 1988_Direct	1	Demographic and Health Survey 1988_Direct	1
	Demographic and Health Survey 1988_Indirect	1	Demographic and Health Survey 1988_Indirect	1
	Demographic Survey 1971_Indirect	1	Demographic Survey 1971_Indirect	1
	Survey 1961_Indirect	1	Survey 1961_Indirect	1
Tonga	Census 2006_Indirect	1	Census 2006_Indirect	0
	Census 1996_Indirect	1	Census 1996_Indirect	0
	Census 1986_Indirect	1	Census 1986_Indirect	0
	Census 1976_Indirect	1	Census 1976_Indirect	0
	WHO Vital Registration Data 2011 version_VR (Single year)	0	WHO Vital Registration Data 2011 version_VR (Single year)	0
Trinidad & Tobago	Multiple Indicator Cluster Survey 2006_Indirect	1	Multiple Indicator Cluster Survey 2006_Indirect	0
	Multiple Indicator Cluster Survey 2000_Indirect	1	Multiple Indicator Cluster Survey 2000_Indirect	0
	Demographic and Health Survey 1987_Direct	1	Demographic and Health Survey 1987_Direct	0
	Demographic and Health Survey 1987_Indirect	1	Demographic and Health Survey 1987_Indirect	0
	World Fertility Survey 1977_Direct	1	World Fertility Survey 1977_Direct	0
	World Fertility Survey 1977_Indirect	1	World Fertility Survey 1977_Indirect	0
	National Life Tables_Single year	0	National Life Tables_Single year	0
	WHO Vital Registration Data 2009 version_Single Year	0	Vital Registration_Single year	0
	WHO Vital Registration Data 2011 version_VR (Single year)	0	WHO Vital Registration Data 2009 version_Single Year	0
			WHO Vital Registration Data 2011 version_VR (Single year)	0
Tunisia	Family Health Survey (PAPFAM) 2001_Direct	1	Family Health Survey (PAPFAM) 2001_Direct	1
	Maternal and Child Health Survey (PAPCHILD) 1994_Direct	1	Maternal and Child Health Survey (PAPCHILD) 1994_Direct	1
	Demographic and Health Survey 1988_Direct	1	Demographic and Health Survey 1988_Direct	1
	Demographic and Health Survey 1988_Indirect	1	Demographic and Health Survey 1988_Indirect	1
	Census 1984_Indirect	1	Census 1984_Indirect	1
	Contraception Prevalence Survey 1983_Indirect	1	Contraception Prevalence Survey 1983_Indirect	1
	World Fertility Survey 1978_Direct	0	World Fertility Survey 1978_Direct	0
	World Fertility Survey 1978_Indirect	0	World Fertility Survey 1978_Indirect	0
	Census 1975_Indirect	1	Census 1975_Indirect	1
	Enquete Nationale Demographique 1968-1969_Sinlge year	1	Enquete Nationale Demographique 1968-1969_Sinlge year	1
	WHO Vital Registration Data 2009 version_Single Year	0	WHO Vital Registration Data 2009 version_Single Year	0
	Vital Registration from Instititue National de la Statistique_Single year	1	Vital Registration from Instititue National de la Statistique_Single year	0
	WHO Vital Registration Data 2011 version_VR (Single year)	0	Vital Registration data from the UN Statistics Division_Single year	0
			WHO Vital Registration Data 2011 version_VR (Single year)	0
Turkey	Demographic and Health Survey 2008_Direct	1	Demographic and Health Survey 2008_Direct	1
	Demographic and Health Survey 2003_Direct	1	Demographic and Health Survey 2003_Direct	1
	Demographic and Health Survey 2003_Indirect	1	Demographic and Health Survey 2003_Indirect	1
	Demographic and Health Survey 1998_Direct	1	Demographic and Health Survey 1998_Direct	1
	Demographic and Health Survey 1998_Indirect	1	Demographic and Health Survey 1998_Indirect	1
	Demographic and Health Survey 1993_Direct	1	Demographic and Health Survey 1993_Direct	1
	Demographic and Health Survey 1993_Indirect	1	Demographic and Health Survey 1993_Indirect	1

	Turkey Population and Health Survey 1988_Indirect	1	Turkey Population and Health Survey 1988_Indirect	1
	Turkey Population and Health Survey 1988_Direct	1	Turkey Population and Health Survey 1988_Direct	1
	Census 1985_Indirect	1	Census 1985_Indirect	1
	Turkey Population and Health Survey 1983_Indirect	1	Turkey Population and Health Survey 1983_Indirect	1
	Census 1980_Indirect	1	Turkey Population and Health Survey 1983_Single year	1
	Turkey Fertility Survey 1978_Indirect	1	Census 1980_Indirect	1
	Census 1975_Indirect	1	Turkey Fertility Survey 1978_Indirect	1
	Census 1970_Indirect	1	Census 1975_Indirect	1
	Turkey Demographic Survey 1967_Indirect	0	Census 1970_Indirect	1
	Turkey Demographic Survey 1967_Direct	1	Haceteppe Survey 1968_Indirect	1
			Turkey Demographic Survey 1967_Indirect	1
			Turkey Demographic Survey 1967_Direct	1
Turkmenistan	Multiple Indicator Cluster Survey 2006_Indirect	1	Multiple Indicator Cluster Survey 2006_Indirect	0
	Demographic and Health Survey 2000_Direct	1	Demographic and Health Survey 2000_Direct	0
	Demographic and Health Survey 2000_Indirect	1	Demographic and Health Survey 2000_Indirect	0
	Trans MONEE Vital Registration_Single year	0	Trans MONEE Vital Registration_Single year	0
	Vital Registration_Single year	0	Vital Registration_Single year	0
	Transmonee Vital Registration Data 2008 version_Single year	0	Transmonee Vital Registration Data 2008 version_Single year	0
	WHO Vital Registration Data 2009 version_Single Year	0	WHO Vital Registration Data 2009 version_Single Year	0
	WHO Vital Registration Data 2011 version_VR (Single year)	0	WHO Vital Registration Data 2011 version_VR (Single year)	0
Turks & Caicos Islands	WHO Vital Registration Data_VR (Single year)_2011	0	WHO Vital Registration Data_VR (Single year)_2011	0
	The State of the World's Children (SOWC) 2009 Estimates_Single year	1	The State of the World's Children (SOWC) 2009 Estimates_Single year	1
Tuvalu	Demographic and Health Survey 2007_Direct	1	Demographic and Health Survey 2007_Direct	0
	Census 1991_Indirect	1	Census 1991_Indirect	0
	WHO Vital Registration Data 2011 version_Single year	1	Vital Registration_Single year	0
	WHO Vital Registration Data 2011 version Moving Average_Single year	0	WHO Vital Registration Data 2011 version_Single year	0
			WHO Vital Registration Data 2011 version Moving Average_Single year	0
Uganda	Malaria Indicator Survey 2009_Direct	1	Malaria Indicator Survey 2009_Direct	0
	Malaria Indicator Survey 2009_Indirect	1	Malaria Indicator Survey 2009_Indirect	0
	Demographic and Health Survey 2009_Direct	0	Demographic and Health Survey 2009_Direct	0
	Demographic and Health Survey 2006_Direct	1	Demographic and Health Survey 2006_Direct	0
	Demographic and Health Survey 2006_Indirect	1	Demographic and Health Survey 2006_Indirect	0
	Census 2002_Direct	1	Census 2002_Direct	0
	Demographic and Health Survey 2000_Direct	1	Demographic and Health Survey 2000_Direct	0
	Demographic and Health Survey 2000_Indirect	1	Demographic and Health Survey 2000_Indirect	0
	Demographic and Health Survey 1995_Direct	1	Demographic and Health Survey 1995_Direct	0
	Demographic and Health Survey 1995_Indirect	1	Demographic and Health Survey 1995_Indirect	0
	Census 1991_Direct	1	Census 1991_Direct	0
	Demographic and Health Survey 1988_Direct	1	Demographic and Health Survey 1988_Direct	0

	Demographic and Health Survey 1988_Indirect<br>Census 1969_Indirect	1<br>1	Demographic and Health Survey 1988_Indirect<br>Census 1969_Indirect	0<br>0
Ukraine	Demographic and Health Survey 2007_Direct<br>Demographic and Health Survey 2007_Indirect<br>Multiple Indicator Cluster Survey 2005_Indirect<br>Census 2001_Indirect<br>Reproductive Health Survey 1999_Direct<br>Census 1989_Indirect<br>Vital Registation Data from Human Mortality Database_Single year<br>Vital Registration Data from State Statistics Committee of Ukraine_Single year<br>WHO Vital Registration Data 2011 version_Single year	1<br>1<br>1<br>1<br>1<br>1<br>1<br>0<br>1	Demographic and Health Survey 2007_Direct<br>Demographic and Health Survey 2007_Indirect<br>Multiple Indicator Cluster Survey 2005_Indirect<br>Census 2001_Indirect<br>Reproductive Health Survey 1999_Direct<br>Census 1989_Indirect<br>Vital Registation Data from Human Mortality Database_Single year<br>Vital Registration Data from State Statistics Committee of Ukraine_Single year<br>WHO Vital Registration Data 2011 version_Single year	0<br>0<br>0<br>0<br>0<br>0<br>0<br>0<br>0
United Arab Emirates	Family Health Survey 1995_Direct<br>Child Health Survey (chs) 1987_Indirect<br>Census 1980_Indirect<br>Census 1975_Indirect<br>Vital Registration Data from Ministry of Planning_Single year<br>Family Health Survey (GFH) 1995_Indirect<br>WHO Vital Registration Data 2011 version_VR (Single year)	1<br>1<br>1<br>1<br>1<br>1<br>0	Family Health Survey 1995_Direct<br>Child Health Survey (chs) 1987_Indirect<br>Census 1980_Indirect<br>Census 1975_Indirect<br>Vital Registration Data from Ministry of Planning_Single year<br>Family Health Survey (GFH) 1995_Indirect<br>WHO Vital Registration Data 2011 version_VR (Single year)	0<br>0<br>0<br>0<br>0<br>0<br>0
United Kingdom	WHO Good Vital Registration Data 2011 version_VR (Single year)	1	WHO Good Vital Registration Data 2011 version_VR (Single year)	1
United States of America	WHO Good Vital Registration Data 2011 version_VR (Single year)	1	WHO Good Vital Registration Data 2011 version_VR (Single year)	1
Uruguay	Census 1996_Indirect<br>Census 1985_Indirect<br>Census 1975_Indirect<br>TABLAS DE VIDA NACIONALES_Single year<br>WHO Good Vital Registration Data 2011 version_VR (Single year)	0<br>0<br>0<br>0<br>1	Census 1996_Indirect<br>Census 1985_Indirect<br>Census 1975_Indirect<br>TABLAS DE VIDA NACIONALES_Single year<br>WHO Good Vital Registration Data 2011 version_VR (Single year)<br>Vital Registration Data from Ministerio de Salud Publica 2010_VR (Single year)	0<br>0<br>0<br>0<br>1<br>0
Uzbekistan	Multiple Indicator Cluster Survey 2006_Indirect<br>Demographic and Health Survey 2002_Direct<br>Demographic and Health Survey 2002_Indirect<br>Multiple Indicator Cluster Survey 2000_Indirect<br>Demographic and Health Survey 1996_Direct<br>Demographic and Health Survey 1996_Indirect<br>WHO Vital Registration Data 2011 version_VR (Single year)	1<br>1<br>1<br>1<br>0<br>0<br>0	Multiple Indicator Cluster Survey 2006_Indirect<br>Demographic and Health Survey 2002_Direct<br>Demographic and Health Survey 2002_Indirect<br>Multiple Indicator Cluster Survey 2000_Indirect<br>Demographic and Health Survey 1996_Direct<br>Demographic and Health Survey 1996_Indirect<br>WHO Vital Registration Data 2011 version_VR (Single year)	0<br>0<br>0<br>0<br>0<br>0<br>0
Vanuatu	Multiple Cluster Indicator Survey 2007_Indirect<br>Census 1999_Indirect<br>Census 1989_Indirect<br>Census 1967_Indirect	1<br>1<br>1<br>1	Multiple Cluster Indicator Survey 2007_Indirect<br>Census 1999_Indirect<br>Census 1989_Indirect<br>Census 1967_Indirect	0<br>0<br>0<br>0

Venezuela	Census 2001_Indirect<br>Census 1990_Indirect<br>Census 1981_Indirect<br>World Fertility Survey 1977_Indirect<br>World Fertility Survey 1977_Direct<br>Vital Registration Data from Ministerio del Poder Popular para la Planificacion y Desarrollo_Single year<br>TABLAS DE VIDA NACIONALES _Single year<br>WHO Vital Registration Data 2011 version_VR (Single year)<br>Vital Registration data from Ministerio del Poder Popular para la Salud 2009_VR (Single year)	1<br>1<br>1<br>1<br>1<br>0<br><br>1<br>0<br>0	Census 2001_Indirect<br>Census 1990_Indirect<br>Census 1981_Indirect<br>World Fertility Survey 1977_Indirect<br>World Fertility Survey 1977_Direct<br>Vital Registration Data from Ministerio del Poder Popular para la Planificacion y<br>Desarrollo_Single year<br>Vital Registration_Single year<br>WHO Vital Registration_Single year<br>TABLAS DE VIDA NACIONALES _Single year<br>WHO Vital Registration Data 2011 version_VR (Single year)<br>Vital Registration data from Ministerio del Poder Popular para la Salud 2009_VR<br>(Single year)<br>Estadisticas Vitales_VR (Single year)	0<br>0<br>0<br>0<br>0<br>0<br>0<br>0<br>0<br>0<br>0<br>0<br>0<br>0
Vietnam	Population Change and Family Planning Survey 2008_Indirect<br>Population Change and Family Planning Survey 2007_Indirect<br>Multiple Indicator Cluster Survey 2006_Indirect<br>Population Change and Family Planning Survey 2006_Indirect<br>Demographic and Health Survey 2002_Direct<br>Demographic and Health Survey 2002_Indirect<br>Multiple Indicator Cluster Survey 2000_Indirect<br>Demographic and Health Survey 1997_Direct<br>Demographic and Health Survey 1997_Indirect<br>Intercensal Demographic Survey 1994_Indirect<br>Census 1989_Indirect<br>Demographic and Health Survey 1988_Direct<br>Demographic and Health Survey 1988_Indirect<br>VR from Health Statistics Yearbook MOH 2009_Single year	0<br>0<br>0<br>0<br>1<br>1<br>1<br>1<br>1<br>0<br>1<br>0<br>0<br>1	Population and Housing Census 2009_Single year<br>Population Change and Family Planning Survey 2008_Indirect<br>Population Change and Family Planning Survey 2008_ Single year<br>Population Change and Family Planning Survey 2007_Indirect<br>Population Change and Family Planning Survey 2007_ Single year<br>Multiple Indicator Cluster Survey 2006_Indirect<br>Population Change and Family Planning Survey 2006_Indirect<br>Population Change and Family Planning Survey 2006_ Single year<br>PCFPS 2005_Single year<br>PCFPS 2004_Single year<br>PCFPS 2003_Single year<br>Demographic and Health Survey 2002_Direct<br>Demographic and Health Survey 2002_Indirect<br>Multiple Indicator Cluster Survey 2000_Indirect<br>PHC 1999_Single year<br>Demographic and Health Survey 1997_Direct<br>Demographic and Health Survey 1997_Indirect<br>Intercensal Demographic Survey 1994_Indirect<br>Census 1989_Indirect<br>Demographic and Health Survey 1988_Direct<br>Demographic and Health Survey 1988_Indirect<br>VR from Health Statistics Yearbook MOH 2009_Single year	0<br>0<br>0<br>0<br>0<br>0<br>0<br>0<br>0<br>0<br>0<br>0<br>0<br>0<br>0<br>0<br>0<br>0<br>0<br>0<br>0<br>0
Yemen	Multiple Indicator Cluster Survey 2006_Direct<br>Multiple Indicator Cluster Survey 2006_Indirect<br>Census 2004_Indirect<br>Census 2004_Single year	1<br>1<br>1<br>1	Multiple Indicator Cluster Survey 2006_Direct<br>Multiple Indicator Cluster Survey 2006_Indirect<br>Census 2004_Indirect<br>Census 2004_Single year	0<br>0<br>0<br>0

	PAP 2003_Direct	1	PAP 2003_Direct	0
	Demographic and Health Survey 1997_Direct	1	Demographic and Health Survey 1997_Direct	0
	Demographic and Health Survey 1997_Indirect	1	Demographic and Health Survey 1997_Indirect	0
	Census 1994_Indirect	1	Census 1994_Indirect	0
	Demographic and Health Survey 1991_Direct	1	Demographic and Health Survey 1991_Direct	0
	Demographic and Health Survey 1991_Indirect	1	Demographic and Health Survey 1991_Indirect	0
	WFS-Individual survey 1979_Indirect	1	WFS-Individual survey 1979_Indirect	0
	WFS-Household survey 1979_Indirect	1	WFS-Household survey 1979_Indirect	0
Zambia	Demographic and Health Survey 2007_Direct	1	Demographic and Health Survey 2007_Direct	0
	Demographic and Health Survey 2007_Indirect	1	Demographic and Health Survey 2007_Indirect	0
	Demographic and Health Survey 2002_Direct	1	Demographic and Health Survey 2002_Direct	0
	Demographic and Health Survey 2002_Indirect	1	Demographic and Health Survey 2002_Indirect	0
	Census 2000_Indirect	1	Census 2000_Indirect	0
	Demographic and Health Survey 1996_Direct	1	Demographic and Health Survey 1996_Direct	0
	Demographic and Health Survey 1996_Indirect	1	Demographic and Health Survey 1996_Indirect	0
	Demographic and Health Survey 1992_Direct	1	Demographic and Health Survey 1992_Direct	0
	Demographic and Health Survey 1992_Indirect	1	Demographic and Health Survey 1992_Indirect	0
	Census 1980_Indirect	1	Census 1980_Indirect	0
	Sample Census of Population 1974_Indirect	1	Sample Census of Population 1974_Indirect	0
	Census 1969_Indirect	1	Census 1969_Indirect	0
Zimbabwe	Multiple Indicator Monitoring Survey 2009_Indirect	1	Multiple Indicator Monitoring Survey 2009_Indirect	0
	Multiple Indicator Monitoring Survey 2009_Direct	1	Demographic and Health Survey 2005_Direct	0
	Demographic and Health Survey 2005_Direct	0	Demographic and Health Survey 2005_Indirect	0
	Demographic and Health Survey 2005_Indirect	0	Census 2002_Indirect	0
	Census 2002_Indirect	1	Demographic and Health Survey 1999_Direct	0
	Demographic and Health Survey 1999_Direct	1	Demographic and Health Survey 1999_Indirect	0
	Demographic and Health Survey 1999_Indirect	1	Inter-censal Demographic Survey 1997_Indirect	0
	Inter-censal Demographic Survey 1997_Indirect	1	Demographic and Health Survey 1994_Direct	0
	Demographic and Health Survey 1994_Direct	1	Demographic and Health Survey 1994_Indirect	0
	Demographic and Health Survey 1994_Indirect	1	Census 1992_Indirect	0
	Census 1992_Indirect	1	Demographic and Health Survey 1988_Direct	0
	Demographic and Health Survey 1988_Direct	1	Demographic and Health Survey 1988_Indirect	0
	Demographic and Health Survey 1988_Indirect	1	Inter-censal Demographic Survey 1987_Indirect	0
	Inter-censal Demographic Survey 1987_Indirect	1	Reproductive Health Survey 1984_Indirect	0
	Reproductive Health Survey 1984_Indirect	1	Census 1982_Indirect	0
	Census 1982_Indirect	1	Census 1969_Indirect	0
	Census 1969_Indirect	1	WHO Vital Registration Data 2009 version_Single Year	0
	WHO Vital Registration Data 2009 version_Single Year	0		

Part B: Details of methods for each country

Country/Year	Country Category	Method to derive U5MR	Alpha value used for U5MR	Method to derive IMR	Alpha value used for IMR
Argentina	Countries with good vital registration data	Model Life Table CD East	N/A	Loess default combined VR	0.231
Australia	Countries with good vital registration data	Loess with default alpha	0.208	Loess with default alpha	0.208
Austria	Countries with good vital registration data	Loess with default alpha	0.227	Loess with default alpha	0.227
Belgium	Countries with good vital registration data	Loess with default alpha	0.223	Loess with default alpha	0.223
Bulgaria	Countries with good vital registration data	Loess with default alpha	0.266	Loess with default alpha	0.266
Canada	Countries with good vital registration data	Loess with default alpha	0.216	Loess with default alpha	0.216
Croatia	Countries with good vital registration data	Loess with default alpha	0.446	Loess with default alpha	0.446
Cyprus	Countries with good vital registration data	Loess with default alpha	0.417	Loess with default alpha	0.417
Czech Republic	Countries with good vital registration data	Loess with default alpha	0.446	Loess with default alpha	0.446
Denmark	Countries with good vital registration data	Loess with default alpha	0.212	Loess with default alpha	0.212
Dominica	Countries with good vital registration data	Loess with default alpha	0.595	Loess with default alpha	0.595
Estonia	Countries with good vital registration data	Loess with default alpha	0.431	Loess with default alpha	0.431
Finland	Countries with good vital registration data	Loess with default alpha	0.212	Loess with default alpha	0.212
France	Countries with good vital registration data	Loess with default alpha	0.208	Loess with default alpha	0.208
Germany	Countries with good vital registration data	Loess with default alpha	0.313	Loess with default alpha	0.313
Greece	Countries with good vital registration data	Loess with default alpha	0.231	Loess with default alpha	0.231
Hungary	Countries with good vital registration data	Loess with default alpha	0.227	Loess with default alpha	0.227
Iceland	Countries with good vital registration data	Loess with default alpha	0.424	Loess with default alpha	0.424
Ireland	Countries with good vital registration data	Loess with default alpha	0.208	Loess with default alpha	0.208
Israel	Countries with good vital registration data	Loess with default alpha	0.357	Loess with default alpha	0.357
Italy	Countries with good vital registration data	Loess with default alpha	0.216	Loess with default alpha	0.216
Japan	Countries with good vital registration data	Loess with default alpha	0.208	Loess with default alpha	0.208

Latvia	Countries with good vital registration data	Loess with default alpha	0.417	Loess with default alpha	0.417
Lithuania	Countries with good vital registration data	Loess with default alpha	0.431	Loess with default alpha	0.431
Luxembourg	Countries with good vital registration data	Loess with default alpha	0.556	Loess with default alpha	0.556
Malta	Countries with good vital registration data	Loess with default alpha	0.446	Loess with default alpha	0.446
Mauritius	Countries with good vital registration data	Loess with default alpha	0.240	Loess with default alpha	0.240
Montenegro	Countries with good vital registration data	Loess with default alpha	1.000	Loess with default alpha	1.000
Netherlands	Countries with good vital registration data	Loess with default alpha	0.208	Loess with default alpha	0.208
New Zealand	Countries with good vital registration data	Loess with default alpha	0.205	Loess with default alpha	0.205
Norway	Countries with good vital registration data	Loess with default alpha	0.212	Loess with default alpha	0.212
Poland	Countries with good vital registration data	Loess with default alpha	0.240	Loess with default alpha	0.240
Portugal	Countries with good vital registration data	Loess with default alpha	0.231	Loess with default alpha	0.231
Romania	Countries with good vital registration data	Loess with default alpha	0.298	Loess with default alpha	0.298
Saint Lucia	Countries with good vital registration data	Loess with default alpha	0.694	Loess with default alpha	0.694
Saint Vincent & the Grenadines	Countries with good vital registration data	Loess with default alpha	0.735	Loess with default alpha	0.735
San Marino	Countries with good vital registration data	Loess using moving average	1.136	Loess using moving average	1.136
Serbia	Countries with good vital registration data	Loess with default alpha	0.500	Loess with default alpha	0.500
Singapore	Countries with good vital registration data	Loess with default alpha	0.227	Loess with default alpha	0.227
Slovakia	Countries with good vital registration data	Loess with default alpha	0.431	Loess with default alpha	0.431
Slovenia	Countries with good vital registration data	Loess with default alpha	0.446	Loess with default alpha	0.446
Spain	Countries with good vital registration data	Loess with default alpha	0.212	Loess with default alpha	0.212
Sweden	Countries with good vital registration data	Loess with default alpha	0.212	Loess with default alpha	0.212
Switzerland	Countries with good vital registration data	Loess with default alpha	0.212	Loess with default alpha	0.212
United Kingdom	Countries with good vital registration data	Loess with default alpha	0.208	Loess with default alpha	0.208
United States of America	Countries with good vital registration data	Loess with default alpha	0.212	Loess with default alpha	0.212
Botswana	Country with high HIV prevalence	Loess with default alpha	0.714	Model Life Table CD West	N/A
Cameroon	Country with high HIV prevalence	Loess with default alpha	0.625	Model Life Table CD North	N/A

Central African Republic	Country with high HIV prevalence	Loess with default alpha	0.833	Model Life Table CD South	N/A
Cote dIvoire	Country with high HIV prevalence	Loess with default alpha	0.556	Model Life Table CD West	N/A
Gabon	Country with high HIV prevalence	Loess with default alpha	2.500	Model Life Table CD West	N/A
Kenya	Country with high HIV prevalence	Loess with default alpha	0.333	Model Life Table CD North	N/A
Lesotho	Country with high HIV prevalence	Loess with default alpha	0.455	Model Life Table CD East	N/A
Malawi	Country with high HIV prevalence	Loess with default alpha	0.385	Model Life Table CD North	N/A
Mozambique	Country with high HIV prevalence	Loess with default alpha	1.000	Model Life Table CD West	N/A
Namibia	Country with high HIV prevalence	Loess with default alpha	1.000	Model Life Table CD North	N/A
Rwanda	Country with high HIV prevalence	Loess with default alpha	0.417	Model Life Table CD North	N/A
South Africa	Country with high HIV prevalence	Loess with default alpha	1.000	Model Life Table CD West	N/A
Swaziland	Country with high HIV prevalence	Loess with default alpha	0.833	Model Life Table CD West	N/A
Uganda	Country with high HIV prevalence	Loess with default alpha	0.556	Model Life Table CD North	N/A
United Republic of Tanzania	Country with high HIV prevalence	Loess with default alpha	0.417	Model Life Table CD North	N/A
Zambia	Country with high HIV prevalence	Loess with default alpha	0.714	Model Life Table CD North	N/A
Zimbabwe	Country with high HIV prevalence	Loess with default alpha	0.455	Model Life Table CD North	N/A
Afghanistan	Other countries	Loess with changed alpha	0.625	Model Life Table CD West	N/A
Albania	Other countries	Loess with default alpha	1.250	Model Life Table CD East	N/A
Algeria	Other countries	Loess with default alpha	0.625	Model Life Table CD South	N/A
Angola	Other countries	Loess with default alpha	1.250	Model Life Table CD North	N/A
Armenia	Other countries	Loess with default alpha	1.667	Model Life Table CD East	N/A
Azerbaijan	Other countries	Loess with default alpha	1.667	Model Life Table CD East	N/A
Bahrain	Other countries	Loess default with VR	0.510	Model Life Table CD West	N/A
Bangladesh	Other countries	Loess with default alpha	0.263	Model Life Table CD West	N/A
Belize	Other countries	Loess with default alpha	1.250	Model Life Table CD West	N/A
Benin	Other countries	Loess with default alpha	1.000	Model Life Table CD North	N/A
Bhutan	Other countries	Loess with default alpha	1.000	Model Life Table CD West	N/A

Bolivia	Other countries	Loess with default alpha	0.556	Model Life Table UN General	N/A
Brazil	Other countries	Loess with default alpha	0.250	Model Life Table CD East	N/A
Burkina Faso	Other countries	Loess with default alpha	0.625	Use the equation derived from data of Sahel countries to derive IMR	N/A
Burundi	Other countries	Loess with default alpha	0.714	Model Life Table CD North	N/A
Cambodia	Other countries	Loess with changed alpha	0.500	Model Life Table CD South	N/A
Cape Verde	Other countries	Loess with default alpha	1.000	Model Life Table CD West	N/A
Chad	Other countries	Loess with default alpha	1.250	Use the equation derived from data of Sahel countries to derive IMR	N/A
China	Other countries	Loess with default alpha	0.714	Model Life Table CD West	N/A
Colombia	Other countries	Loess with default alpha	0.417	Loess with default alpha	0.417
Comoros	Other countries	Loess with default alpha	1.250	Model Life Table CD West	N/A
Congo	Other countries	Loess with default alpha	2.500	Model Life Table CD North	N/A
Cook Islands	Other countries	Loess with default alpha	1.000	Model Life Table CD West	N/A
Dem. People's Republic of Korea	Other countries	Adjusted		Adjusted	
Democratic Republic of the Congo	Other countries	Adjusted		Model Life Table CD South	N/A
Djibouti	Other countries	Loess with default alpha	1.667	Model Life Table CD East	N/A
Dominican Republic	Other countries	Loess with default alpha	0.455	Model Life Table CD West	N/A
Ecuador	Other countries	Loess with default alpha	0.455	Loess with default alpha	0.455
Egypt	Other countries	Loess with default alpha	0.417	Model Life Table CD West	N/A
El Salvador	Other countries	Loess with default alpha	0.385	Model Life Table CD West	N/A
Equatorial Guinea	Other countries	Loess with changed alpha	5.000	Loess with changed alpha	5
Eritrea	Other countries	Loess with changed alpha	1.250	Model Life Table CD North	N/A
Ethiopia	Other countries	Loess with default alpha	1.000	Model Life Table CD North	N/A
Fiji	Other countries	Loess with default alpha	1.250	Model Life Table CD West	N/A

Gambia The	Other countries	Loess with default alpha	1.000	Use the equation derived from data of Sahel countries to derive IMR	N/A
Georgia	Other countries	Loess with default alpha	1.667	Model Life Table CD East	N/A
Ghana	Other countries	Loess with default alpha	0.417	Model Life Table CD North	N/A
Guatemala	Other countries	Loess with default alpha	0.417	Loess with default alpha	0.385
Guinea	Other countries	Loess with default alpha	0.833	Model Life Table CD North	N/A
Guinea-Bissau	Other countries	Adjusted	1.667	Model Life Table CD North	N/A
Guyana	Other countries	Loess with default alpha	0.833	Model Life Table CD West	N/A
Haiti	Other countries	Loess with default alpha	0.714	Model Life Table CD West	N/A
Honduras	Other countries	Loess with default alpha	0.455	Model Life Table CD West	N/A
India	Other countries	Loess with default alpha	0.833	Model Life Table CD West	N/A
Indonesia	Other countries	Loess with default alpha	0.455	Model Life Table CD North	N/A
Iran	Other countries	Loess with default alpha	0.385	Model Life Table CD West	N/A
Iraq	Other countries	Loess with default alpha	0.556	Model Life Table CD West	N/A
Jamaica	Other countries	Loess with default alpha	1.250	Model Life Table CD West	N/A
Jordan	Other countries	Loess with default alpha	0.385	Loess with default alpha	0.385
Kazakhstan	Other countries	Loess default with VR trend	1.000	Model Life Table CD East	N/A
Kiribati	Other countries	Loess with default alpha	1.250	Model Life Table CD West	N/A
Kuwait	Other countries	Loess with default alpha	0.294	Loess with default alpha	0.294
Kyrgyzstan	Other countries	Loess default with VR trend	2.500	Model Life Table CD East	N/A
Lao PDR	Other countries	Model Life Table CD West		Loess with default alpha	0.714
Lebanon	Other countries	Loess with default alpha	1.000	Model Life Table CD West	N/A
Liberia	Other countries	Loess with changed alpha	0.500	Model Life Table CD West	N/A
Libya	Other countries	Loess with default alpha	1.250	Model Life Table CD North	N/A
Liechtenstein	Other countries	Provisional		Provisional	
Madagascar	Other countries	Loess with default alpha	0.833	Model Life Table CD North	N/A
Malaysia	Other countries	Loess with default alpha	1.250	Model Life Table CD West	N/A

Maldives	Other countries	Loess with default alpha	0.625	Loess with default alpha	0.625
Mali	Other countries	Loess with default alpha	1.250	Use the equation derived from data of Sahel countries to derive IMR	N/A
Marshall Islands	Other countries	Loess with default alpha	1.250	Model Life Table CD West	N/A
Mauritania	Other countries	Loess with default alpha	0.556	Use the equation derived from data of Sahel countries to derive IMR	N/A
Mexico	Other countries	Loess default with VR	0.455	Loess default with VR New	0.417
Micronesia (Federated States of)	Other countries	Loess with default alpha	2.500	Model Life Table CD West	N/A
Mongolia	Other countries	Loess with default alpha	0.714	Model Life Table CD West	N/A
Morocco	Other countries	Loess with default alpha	0.556	Model Life Table CD South	N/A
Myanmar	Other countries	Loess with default alpha	1.000	Model Life Table CD West	N/A
Nauru	Other countries	Adjusted	2.500	Model Life Table CD West	N/A
Nepal	Other countries	Loess with default alpha	0.833	Loess with default alpha	0.833
Nicaragua	Other countries	Loess with default alpha	0.500	Model Life Table CD West	N/A
Niger	Other countries	Loess with changed alpha	0.500	Use the equation derived from data of Sahel countries to derive IMR	N/A
Nigeria	Other countries	Loess with changed alpha	0.750	Model Life Table CD North	N/A
Occupied Palestinian Territory	Other countries	Loess with default alpha	0.625	Loess with default alpha	0.625
Oman	Other countries	Loess with default alpha	0.625	Loess with default alpha	0.625
Pakistan	Other countries	Loess with default alpha	0.333	Model Life Table CD East	N/A
Panama	Other countries	Loess with default alpha	0.714	Loess with default alpha	0.714
Papua New Guinea	Other countries	Loess with default alpha	1.000	Model Life Table CD West	N/A
Paraguay	Other countries	Loess with default alpha	0.500	Model Life Table CD West	N/A
Peru	Other countries	Loess with default alpha	0.294	Model Life Table UN Latin American	N/A
Philippines	Other countries	Loess with default alpha	0.714	Model Life Table CD North	N/A

Qatar	Other countries	Loess with default alpha	0.926	Loess with default alpha	0.926
Republic of Korea	Other countries	Loess with default alpha	0.397	Model Life Table CD West	N/A
Republic of Moldova	Other countries	Loess with default alpha	2.500	Model Life Table CD West	N/A
Russian Federation	Other countries	Loess default with adjusted VR	0.385	Loess default with adjusted VR	0.385
Samoa	Other countries	Loess with changed	0.625	Model Life Table CD West	N/A
Sao Tome & Principe	Other countries	Loess with default alpha	1.250	Model Life Table CD North	N/A
Saudi Arabia	Other countries	Model Life Table CD West	N/A	Loess with changed alpha	1
Senegal	Other countries	Loess with default alpha	0.417	Use the equation derived from data of Sahel countries to derive IMR	N/A
Seychelles	Other countries	Loess with default alpha	0.490	Loess with default alpha	0.49
Sierra Leone	Other countries	Loess with changed alpha	0.357	Model Life Table CD South	N/A
Solomon Islands	Other countries	Loess with default alpha	1.250	Model Life Table CD West	N/A
Somalia	Other countries	Adjusted		Model Life Table CD North	N/A
Sri Lanka	Other countries	Loess with default alpha	0.714	Model Life Table CD West	N/A
Sudan	Other countries	Loess with default alpha	0.625	Model Life Table CD North	N/A
Suriname	Other countries	Loess with default alpha	2.500	Model Life Table CD East	N/A
Syria	Other countries	Loess with default alpha	0.385	Model Life Table CD West	N/A
Tajikistan	Other countries	Loess with changed alpha	0.750	Model Life Table CD East	N/A
TFYR Macedonia	Other countries	Model Life Table CD East	N/A	Loess default with VR	0.446
Thailand	Other countries	Loess with default alpha	0.250	Model Life Table CD West	N/A
Timor Leste	Other countries	Loess with default alpha	1.000	Model Life Table CD East	N/A
Togo	Other countries	Loess with default alpha	1.000	Loess with default alpha	1.000
Tonga	Other countries	Loess with default alpha	1.250	Model Life Table CD West	N/A
Trinidad & Tobago	Other countries	Loess with default alpha	1.250	Model Life Table CD East	N/A
Tunisia	Other countries	Loess with default alpha	0.625	Model Life Table CD West	N/A
Turkey	Other countries	Loess with changed alpha	0.250	Loess with changed alpha	0.250

Turkmenistan	Other countries	Loess with changed alpha	2.500	Model Life Table CD East	N/A
Tuvalu	Other countries	Loess with default alpha	1.667	Model Life Table CD West	N/A
Ukraine	Other countries	Loess Changed alpha with VR	0.833	Model Life Table CD West	N/A
United Arab Emirates	Other countries	Loess with default alpha	0.833	Model Life Table CD West	N/A
Uzbekistan	Other countries	Loess with default alpha	1.667	Model Life Table CD East	N/A
Vanuatu	Other countries	Loess with default alpha	1.250	Model Life Table CD West	N/A
Venezuela	Other countries	Loess with default alpha	1.000	Model Life Table CD West	N/A
Vietnam	Other countries	Loess with default alpha	1.000	Model Life Table CD North	N/A
Yemen	Other countries	Loess with default alpha	0.625	Model Life Table CD West	N/A
Andorra	Other countries	Loess with default alpha	0.625	Loess with default alpha	0.625
Antigua & Barbuda	Other countries	Loess with default alpha	1.471	Loess with default alpha	1.471
Bahamas	Other countries (with only vital registration data)	Loess with default alpha	0.676	Loess with default alpha	0.676
Barbados	Other countries (with only vital registration data)	Loess with default alpha	0.472	Loess with default alpha	0.472
Belarus	Other countries (with only vital registration data)	Loess with default alpha	0.417	Loess with default alpha	0.417
Bosnia & Herzegovina	Other countries (with only vital registration data)	Loess with default alpha	0.658	Loess with default alpha	0.658
Brunei	Other countries (with only vital registration data)	Loess with default alpha	0.962	Loess with default alpha	0.962
Chile	Other countries (with only vital registration data)	Loess default with VR	0.250	Loess default with VR	0.25
Costa Rica	Other countries (with only vital registration data)	Loess default with VR	0.227	Loess default with VR	0.227
Cuba	Other countries (with only vital registration data)	Loess with default alpha	0.278	Loess with default alpha	0.278
Grenada	Other countries (with only vital registration data)	Loess with default alpha	1.087	Loess with default alpha	1.087
Monaco	Other countries (with only vital registration data)	Loess with default alpha	0.625	Loess with default alpha	0.625
Niue	Other countries (with only vital registration data)	Loess default based on moving average	0.568	Model Life Table CD West	N/A
Palau	Other countries (with only vital registration data)	Loess with default alpha	1.190	Loess with default alpha	1.19
Saint Kitts & Nevis	Other countries (with only vital registration data)	Loess with default alpha	0.714	Loess with default alpha	0.715
Uruguay	Other countries (with only vital registration data)	Loess with default alpha	0.250	Loess with default alpha	0.25
