# Supplementary material for: Child Mortality Estimation: Accelerated Progress in Reducing Global Child Mortality, 1990–2010
Source: PLoS Med. 2012 Aug 28;9(8):e1001303. doi: 10.1371/journal.pmed.1001303 (PMC3429379; doi:10.1371/journal.pmed.1001303)
Supplement: Text S1 — Details of loess regression in countries with generalized HIV/AIDS epidemics. (PDF) [file pmed.1001303.s002.pdf]

Text S1 Details of LOESS regression in countries with generalized HIV/AIDS epidemic

Countries with high HIV prevalence are defined by a prevalence rate exceeding 5% at any point in the epidemic period. Additional adjustment was applied to 17 countries with high HIV prevalence.

Estimates of child mortality for populations lacking accurate registration of births and deaths are almost entirely derived from reports of mothers about the survival of their children. The most common approach is the full birth history (used in all Demographic and Health Surveys and some Multiple Indicator Cluster Surveys), whereby each woman aged 15 to 49 at the time of the survey is asked for the date of birth and, if the child has died, age at death of each live-born child she has had. Child mortality is then estimated from the information on births and deaths for periods up to 25 years before the survey. To be valid estimates of population-level child mortality, the births must be representative of all births in the population for the entire period. It is unlikely that this condition is ever perfectly met: births to mothers who have died or migrated out of the population will not be reported, and for periods long before the survey births to older mothers will not be represented because the mothers will have been age 50 or over at the time of the survey. It is normally assumed that any bias introduced by lack of representativeness is small, but this will not be the case in populations severely affected by HIV where HIV+ children will be more likely to die than other children, and will also be less likely to be reported since their mothers will have been more likely to die also. Child mortality estimates will thus be biased downwards. The magnitude of the bias will depend on the extent to which the elevated under-five mortality of HIV+ children is not reported because of the deaths of their mothers. Precise estimates of the bias would require a great deal of information about the HIV epidemic, such as the distribution of births to HIV+ women by the duration of their infection, vertical transmission rates, survival times of both mothers and children from the time of the birth, and much else besides. Such information is not typically available, so we have adopted a number of simplifications.

We start with the latest information of a national population and its HIV epidemic from UNAIDS. UNAIDS typically provides the annual number of births from 1980 onwards, and the HIV prevalence among pregnant women aged 15 to 49. For each year, the births are divided into three components: births to HIV- mothers, assumed to be all HIV-; HIV- births to HIV+ mothers; and HIV+ births to HIV+ mothers (no distinction is made between children infected at or before birth and those infected after birth). For births in each year, deaths under age 5 in the subsequent five years are calculated for each component. For the HIV- births, risks of dying are obtained from a model life table (Coale-Demeny “West” family) with a U5MR approximating a best estimate as to U5MR in the HIV- population. HIV- children are assumed to have the same risk regardless of the HIV status of the mother. For HIV+ births, a mortality schedule derived from cohort studies, with a probability of dying by age 5 of 62.5% is used; it is assumed that ARV treatment has had no effect on these risks for the relevant period.

The next step is to estimate how many of these births and under-five deaths will go unreported at a particular survey because of deaths of the mothers. We first assume that HIV- women have no mortality, so all their births and under-five deaths are reported. We then assume that births to HIV+ women occur on average to women after 4 years of infection (4 years was chosen because it allows for a reduction of fertility among women who are HIV+ of approximately 25%).

A survival curve from first infection, again derived from cohort studies, with a median survival time of about 9.5 years, was used to create a survival curve from 4 years after infection, and the probabilities of surviving from a particular year to the year of a given survey (assumed to be at the end of a year) were used to calculate the proportion of the births and child deaths (whether HIV- or HIV+) of HIV+ mothers reported by the survey.

For any time period before a survey, the true and the "reported" births and under-five deaths are summed, and the estimated bias is calculated as the ratio of the reported under-five deaths divided by the reported births to the corresponding value for the true numbers. Survey estimates of under-five mortality are then adjusted by dividing by the bias estimates for each time period.

To capture the extraordinary rapid changes in child mortality driven by HIV/AIDS over the epidemic period in the high HIV/AIDS epidemic countries, the Loess regression models described in section 3 above were fitted to data points for under-five mortality from all other causes than HIV/AIDS. By default, the α value for each country was determined as 5/(number of independent data series in the country). UNAIDS estimates of HIV/AIDS under-five mortality then were added to estimates from the regression model to generate the final estimates. The steps for under-five mortality rate estimation in countries with high HIV/AIDS prevalence are as follows:

a. Exclude individual data points from SBH indirect data series from earlier than 1990 if age group of mothers is less than 25.
b. Exclude individual data points from SBH indirect data series from 1990 onwards if age group of mothers is less than 25 or more than 39 years.
c. Evaluate whether SBH indirect data series from 1990 onwards for age group of mothers 25-39 years are to be included:
    - Fit a regression curve with indirect data series with survey year 1990 onwards excluded.
    - Include indirect data series with survey year 1990 onwards if two or more of the survey estimated U5MR for age groups 25-39 are greater than regression model estimates for the same years.
d. Subtract UNAIDS estimates of HIV/AIDS under-five mortality from data points in the epidemic period. Outside the epidemic period child mortality from HIV/AIDS is minor and so step 4 results in a data set assumed to hold under-five mortality from all other causes than HIV/AIDS.
e. Fit Loess regression model to all data points for under-five mortality from all other causes than HIV/AIDS.
f. Extrapolate regression curve to 2010.
g. Add UNAIDS estimates of HIV/AIDS under-five mortality to regression estimates in the epidemic period.
