# Supplementary material for: Child Mortality Estimation: Accelerated Progress in Reducing Global Child Mortality, 1990–2010
Source: PLoS Med. 2012 Aug 28;9(8):e1001303. doi: 10.1371/journal.pmed.1001303 (PMC3429379; doi:10.1371/journal.pmed.1001303)
Supplement: Text S2 — Calculating numbers of infant and under-five deaths. (PDF) [file pmed.1001303.s003.pdf]

Text S2 Calculating numbers of infant and under-5 deaths

To calculate the absolute number of deaths among infants and children under five in a given year and country, an abridged life table approach is used.

Terminology:

$_nq_x$ is the probability of dying between age x and age x+n
D_0 is the number of deaths for age < 1 year
D_1 is the number of deaths for age group 1-4 years
P_0 is the mid-year population for age < 1 year
P_1 is the mid-year population for age group 1-4 years
$M_0 = D_0/P_0$, death rate for age <1,
$M_1 = D_1/P_1$, death rate for age group 1-4 years,

The central death rates of age groups 0 and 1-4 years are used, computed back from the probability of dying $_5q_0$ and $_1q_0$, to which population is applied. First, $_1q_4$ is derived from $_1q_0$ and $_5q_0$ as follows: $_4q_1 = (_5q_0 - {_1q_0})/(1-{_1q_0})$. Then, for each age group 0 and 1-4, the central death rate M_0 and M_1 is computed as follows:

$$M_0 = {_1q_0}/[1-(1-a)*{_1q_0}]$$

$$M_1 = 4*{_4q_1} / 4*[1-(1-0.4)*{_4q_1}],$$

where a is the fraction of year lived by an infant

= 0.1 for low mortality country and a= 0.3 for high mortality country.

Finally, country population estimates from the *World Population Prospects: the 2010 revision* produced by UN DESA/Population Division are used to the death rates to obtain the number of deaths. For greater accuracy, the number of deaths is calculated separately for infants and children age 1-4 years.
